# Which Physical Therapy Intervention Is Most Effective in Reducing Secondary Lymphedema Associated with Breast Cancer? A Systematic Review and Network Meta-Analysis

**DOI:** 10.3390/jcm14196762

**Published:** 2025-09-24

**Authors:** Raúl Alberto Aguilera-Eguía, Pamela Serón, Ruvistay Gutiérrez-Arias, Brenda Herrera-Serna, Víctor Pérez-Galdavini, Gloria Inostroza-Reyes, Cristian Yáñez-Baeza, Héctor Fuentes-Barría, Hellen Belmar Arriagada, Jaqueline Inostroza-Quiroz, Mariana Melo-Lonconao, Miguel Alarcón-Rivera, Mario Muñoz-Bustos, Mónica Pinzón-Bernal, Patricia López-Soto, Ángel Roco-Videla, Lisse Angarita-Dávila, Xavier Bonfill, Carlos Zaror

**Affiliations:** 1 Departamento de Salud Pública, Facultad de Medicina, Universidad Católica de la Santísima Concepción, Concepción 4090541, Chile; raguilerae@ucsc.cl (R.A.A.-E.); hellen.belmar.a@gmail.com (H.B.A.); mariana.melo@uss.cl (M.M.-L.); 2 Doctorado en Metodología de la Investigación Biomédica y Salud Pública, Universidad Autónoma de Barcelona, 08193 Barcelona, Spain; jacqueline.inostroza@ufrontera.cl (J.I.-Q.);; 3 Departamento de Ciencias de la Rehabilitación, Universidad de La Frontera, Temuco 4811230, Chile; pamela.seron@ufrontera.cl; 4 Centro de Excelencia CIGES, Facultad de Medicina, Universidad de La Frontera, Temuco 4811230, Chile; 5 Departamento de Apoyo en Rehabilitación Cardiopulmonar Integral, Instituto Nacional del Tórax, Santiago 7500921, Chile; ruvistay.gutierrez@gmail.com; 6 Exercise and Rehabilitation Sciences Institute, Faculty of Rehabilitation Sciences, Universidad Andres Bello, Santiago 7591538, Chile; 7 Programa de Odontología, Institución Universitaria Visión de las Américas, Pereira 660003, Colombia; brenda.herrera@uam.edu.co; 8 Departamento de Ciencias Clínicas y Preclínicas, Facultad de Medicina, Universidad Católica de la Santísima Concepción, Concepción 4090541, Chile; victorperez@ucsc.cl (V.P.-G.); ginostroza@ucsc.cl (G.I.-R.); cyanez@ucsc.cl (C.Y.-B.); 9 Vicerrectoría de Investigación e Innovación, Universidad Arturo Prat, Iquique 1100000, Chile; hefuentes_@unap.cl; 10 Escuela de Odontología, Facultad de Odontología, Universidad Andres Bello, Concepción 3349001, Chile; 11 Escuela de Ciencias del Deporte y Actividad Física, Facultad de Salud, Universidad Santo Tomás, Talca 3460000, Chile; mrivera3@santotomas.cl; 12 Facultad de Medicina, Universidad Católica del Maule, Talca 3480112, Chile; 13 Departamento de Kinesiología, Facultad de Medicina, Universidad de Concepción, Concepción 4070386, Chile; 14 Departamento de Movimiento Humano, Universidad Autónoma de Manizales, Manizales 170001, Colombia; myamile@autonoma.edu.co; 15 Departamento de Salud Oral, Facultad de Salud, Universidad Autónoma de Manizales, Manizales 170001, Colombia; 16 Dirección de Desarrollo y Postgrados, Universidad Autónoma de Chile, Santiago 7500912, Chile; angel.roco@uautonoma.cl; 17 Escuela de Nutrición y Dietética, Facultad de Medicina, Universidad Andres Bello, Concepción 3349001, Chile; 18 Clinical Epidemiology and Public Health, Biomedical Research Institut Sant Pau, 08025 Barcelona, Spain; 19 Department of Pediatric Dentistry and Orthodontics, Faculty of Dentistry, Universidad de La Frontera, Temuco 4811230, Chile; 20 Center for Research in Epidemiology, Economics and Oral Public Health (CIEESPO), Faculty of Dentistry, Universidad de La Frontera, Temuco 4811230, Chile

**Keywords:** breast cancer lymphedema, breast cancer treatment-related lymphedema, physical therapy modalities, physical therapy

## Abstract

**Background**: Breast cancer-related lymphedema (BCRL) is a common complication that impairs function and quality of life (QoL). The comparative effectiveness of physical therapy interventions (PTIs) remains unclear. This systematic review and network meta-analysis (NMA) was conducted to identify the most effective PTIs for BCRL management. **Methods**: A systematic search of Medline/PubMed, LILACS, CENTRAL, PEDro, and CINAHL was conducted up to July 2024. Eligible studies were randomized controlled trials (RCTs) involving women with BCRL, evaluating PTIs delivered alone or in combination. Primary outcomes were lymphedema volume, volume reduction, percentage reduction, QoL, and pain. Secondary outcomes included range of motion (ROM), grip strength, and adverse events. A frequentist NMA was performed, and certainty of evidence (CoE) was assessed using the GRADE approach. **Results**: Eighty-three RCTs were identified, of which twenty-six (1203 participants) were included in the NMA, assessing 23 PTIs. Based on moderate CoE, yoga was among the most effective interventions for improving QoL within 6 months compared to usual standard care (USC). The multimodal approach, with or without a home exercise program, showed intermediate benefits for external rotation and may also improve shoulder abduction (low to moderate CoE). No intervention demonstrated clear superiority over USC for other outcomes. Adverse events were reported with kinesiotaping and compression measures. **Conclusions**: The evidence supports yoga and multimodal programs as potential short-term strategies for improving QoL and shoulder mobility in women with BCRL. However, the predominance of low-to-very-low CoE underscores the need for individualized clinical decisions and future high-quality RCTs with standardized comparators, larger samples, and longer follow-up. The consistent use of standardized comparators will be crucial in improving network connectivity and enabling more robust and comprehensive comparisons across multiple interventions.

## 1. Introduction

Recent epidemiology shows that breast cancer was the most frequently diagnosed cancer in 2022, with approximately 2.5 million new cases worldwide [1]. Lymphedema secondary to breast cancer (BCRL) is one of the most underestimated and debilitating complications of the treatment for breast cancer [2]. According to the 2023 Consensus Document of the International Society of Lymphology (ISL), lymphedema is defined as an external and/or internal manifestation of lymphatic system insufficiency and impaired lymph transport, recognized as a disease by the International Classification of Diseases (ICD) of the World Health Organization [3]. BCRL occurs due to the interruption of lymphatic fluid and other factors, such as total mastectomy, axillary dissection, positive lymph nodes, radiotherapy, taxane use, and obesity [3,4]. The incidence of BCRL varies between 3% and 65%, depending on the type of intervention received and the follow-up time [3,5]. Prior studies have shown that the incidence of BCRL is greater in people subjected to a dissection of the axillary lymphatic glands (33.3%) compared to those who received a sentinel node biopsy (3.4%) at 5 years post-treatment [6]. Clinically, patients refer to a sensation of heaviness or rigidity in the extremity, movement limitation, pain, and, in severe cases, hardening and fibrosis, which affects functioning and psychological health, diminishing their quality of life (QoL) [7,8]. The physical therapy interventions (PTIs) for BCRL include diverse interventions, such as complex decongestive therapy, manual lymphatic draining, low-level laser therapy, shock waves, pneumatic pumps, kinesiotaping, resistance training, the multimodal approach, aerobics, water-based training, yoga, Pilates, and combinations of these [9]. In 2020, the APTA practical clinical guideline focused solely on reducing lymphedema volume, excluding studies on other important outcomes, such as QoL and pain management. The guideline further restricted its application by excluding studies published in languages other than English and those outside the specified time frame. This study addresses these limitations by broadening the focus to evaluate a more complete range of interventions, including volume reduction, QoL, and pain in patients with BCRL. The restrictions on language and publication period were addressed by including studies in English, Spanish, and Portuguese and by extending the search until July 2024. A systematic NMA enabled direct and indirect comparisons, identifying the most effective interventions and strengthening the basis for clinical decision-making [10]. Although several systematic reviews (SRs) have evaluated the effectiveness of different physical therapy interventions (PTIs) for reducing BCRL [10,11,12,13,14,15], to our knowledge, no previous SR has comprehensively compared the relative effectiveness of these interventions or assessed the probability of success of those not considered in prior RCTs.

The research question for this systematic review is as follows: Are there physical therapy interventions, or combinations of these, that are more effective than others in reducing lymphedema, improving quality of life, alleviating pain, and enhancing joint range of motion and muscular strength in patients with breast cancer-related lymphedema (BCRL)? Guided by this question, the objective of the review was to determine which physical therapy interventions, alone or in combination, are most effective in reducing lymphedema, improving quality of life, alleviating pain, and increasing joint range of motion and muscular strength in patients with BCRL.

## 2. Materials and Methods

### 2.1. Protocol and Registration

This SR was conducted following the recommendations of the Cochrane Handbook for Systematic Reviews of Interventions [16]. This SR and network meta-analysis (NMA) was reported in accordance with the Preferred Reporting Items for Systematic Reviews and Meta-Analyses (PRISMA) checklist for NMAs [17] (Supplement S1). The protocol was registered in PROSPERO (CRD42022323541) and published in an open-access journal [18].

### 2.2. Eligibility Criteria

RCTs in English, Spanish, or Portuguese that involved women older than 15 years with BCRL were included. Studies were considered eligible if they assessed PTIs, delivered either alone or in combination, and we compared them either to each other or against one of the following control conditions: usual standard care (USC), education about BCRL, or no additional intervention.

The following PTIs were evaluated: complex decongestive therapy, manual lymphatic drainage, low-level laser therapy, pneumatic pumps, kinesiotaping, high-, moderate-, and low-intensity resistance exercises (both supervised and unsupervised), endurance training and water-based endurance training, yoga, Pilates, shockwave therapy, and any combination of these interventions.

The primary outcomes of interest were lymphedema volume, volume reduction, percent reduction, QoL, and pain intensity (see Supplement S2). Additionally, secondary outcomes were considered, including range of motion (ROM) assessed with goniometry or another validated method, grip strength measured with dynamometry or another validated method, and adverse events related to the PTIs, such as increased lymphedema and pain (see Supplement S3).

### 2.3. Treatment Nodes

For the analysis of PTIs in BCRL, all eligible interventions were classified as treatment nodes and a comparator node, labeled as USC. The classification was based on the nature and fundamental principles of each intervention. Studies were assigned to nodes regardless of specific characteristics, such as modality, duration, intensity, or frequency. When an intervention included more than one treatment type, a separate node was created for each component to maintain clarity and analytical precision. This structure enabled a coherent, comparative, and systematic evaluation of PTIs for BCRL. The comparator node, labeled as USC, served as the reference category in the network meta-analysis and included three types of comparators commonly used in clinical trials: (A) usual standard care (USC): defined in each study as routine care (e.g., advice on exercise, self-care strategies, use of compression, or clinical surveillance); (B) education-only interventions: provision of information about BCRL, skincare, and safety precautions, without supervised exercise or manual therapy; and (C) no additional intervention: observation or wait-list groups without active treatment. A detailed description of the structure and definitions of all treatment and control nodes is provided in Supplement S4.

### 2.4. Information and Search Sources

#### Search Strategy

The systematic database search covered publications from inception until July 2024. Specifically, the search started in 1966 for MEDLINE, 1974 for EMBASE, 1982 for LILACS, 2008 for the Cochrane Central Register of Controlled Trials, 1999 for PEDro, and 1984 for CINAHL. The details of the search strategy are described in Supplement S5. Additionally, a search of the European grey literature database (http://www.opengrey.eu; accessed on 25 July 2024) was performed. The reference lists of the included studies and previous systematic reviews were also examined. Furthermore, registers of RCTs (www.registroensayosclinicos.org; accessed on 25 July 2024, https://clinicaltrials.gov; accessed on 25 July 2024, and https://www.who.int/clinical-trials-registry-platform; accessed on 25 July 2024), public access policies (https://publicaccess.nih.gov; accessed on 25 July 2024), and preprint servers (https://www.medrxiv.org; accessed 25 on July 2024 and https://www.biorxiv.org; accessed on 25 July 2024) were examined.

### 2.5. Selection Process

Following training exercises and calibration, pairs of reviewers independently reviewed all titles, summaries, and full texts of the studies identified as potentially eligible through the Rayyan Intelligent Systematic Review software (www.rayyan.ai). A third reviewer resolved discrepancies.

### 2.6. Data Compiling and Item Processing

For each study that met the eligibility criteria, six pairs of researchers performed the data extraction independently, using a standardized form previously validated by a pilot process. The spreadsheet included the following sections: study identification, study design/setting, study population and participant demographics, baseline characteristics, details of the intervention and control conditions, outcome data of interest, and follow-up times. A third reviewer intervened to resolve any discrepancies.

### 2.7. Risk of Bias and Certainty in the Evaluation of Evidence

The risk of bias was independently evaluated using the Cochrane Risk of Bias Tool (ROB 2.0) [19,20]. ROB 2.0 evaluates the following domains: bias arising from the randomization process, bias due to deviations from intended interventions, bias due to missing outcome data, bias in outcome measurement, and bias in the selection of reported results. The study-level risk of bias was classified as low, with some concerns, or high. Discrepancies were resolved through the mediation of a third reviewer.

### 2.8. Data Synthesis

A frequentist NMA was conducted using multivariate meta-analysis in Stata 18 software (Stata Corp LP, College Station, TX, USA) [20]. Analyses were primarily performed using a random-effects model, anticipating clinical and methodological heterogeneity across studies. For comparisons with very few studies, a common-effect (fixed-effect) model was applied, as between-study variance (τ^2^) could not be reliably estimated under these conditions. Network plots were created to illustrate the network geometry, with interventions organized into treatment nodes according to the pre-established selection criteria. Usual standard care (USC) was used as the reference comparator for all interventions. Heterogeneity was assessed by visual inspection of forest plots and quantified with the I^2^ statistic, acknowledging the uncertainty of this measure in analyses with few studies. These considerations were explicitly incorporated into the evaluation of the certainty of evidence (CoE) using the GRADE approach. Network consistency was evaluated through the design-by-treatment model and by applying the node-splitting technique to compare direct and indirect evidence for each comparison. When the NMA was not feasible due to data insufficiency or lack of network connectivity among studies, pairwise meta-analyses were conducted using a random-effects model, with between-study variance estimated through the restricted maximum likelihood method (REML). The results were presented narratively when neither NMA nor pairwise meta-analysis could be performed. For continuous outcomes, the effects of interventions were estimated using the mean difference (MD) with 95% confidence intervals (CIs) or the standardized mean difference (SMD) when studies used different measurement scales. Funnel plots were planned to assess publication bias when more than 10 studies were available for a given outcome. Attempts were made to obtain missing data and details of unreported outcomes by contacting the original study authors. No additional statistical methods were applied, and no further imputation was performed for missing data.

### 2.9. Additional Analysis

A subgroup analysis was planned according to different follow-up periods and QoL tools, along with a sensitivity analysis excluding studies with an overall high risk of bias. During the review process, it was observed that USC was not always the most common intervention. For this reason, a sensitivity analysis was performed using complex decongestive therapy as the principal comparator, given that it was the most frequently used intervention in most of the outcomes analyzed.

### 2.10. Certainty of Evidence and Conclusions of This Report

The certainty of evidence (CoE) was evaluated using the GRADE approach for the NMA [21]. The GRADE domains included risk of bias, inconsistency, indirectness, publication bias, imprecision, intransitivity, and incoherence [21,22,23,24,25]. Each domain for each result and comparison was independently assessed by two methodologists. Any discrepancies were resolved through consensus. A minimally contextualized approach was used, with a null effect as the threshold to assess imprecision [24]. Interventions were classified from the most to the least effective by considering the estimates of effect and the certainty of the evidence. The results are displayed graphically to facilitate interpretation [24,26].

## 3. Results

### 3.1. Study Selection

**Figure 1:**
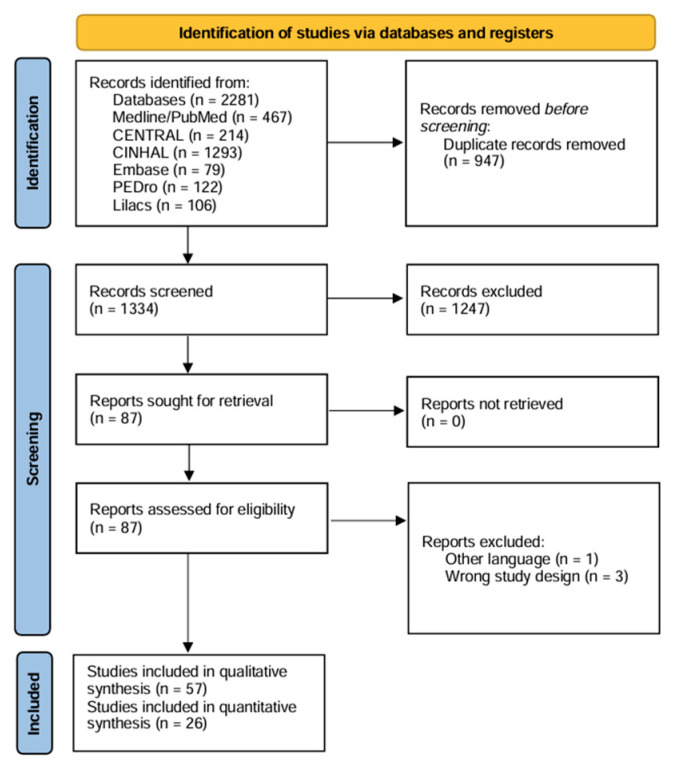
Flowchart of the systematic literature review.

The search strategy identified 2260 citations, of which 83 were included (Figure 1). Of these, 26 were included in the NMA [27,28,29,30,31,32,33,34,35,36,37,38,39,40,41,42,43,44,45,46,47,48,49,50,51,52], while the other 57 could not be integrated because they were not connected to the network [53,54,55,56,57,58,59,60,61,62,63,64,65,66,67,68,69,70,71,72,73,74,75,76,77,78,79,80,81,82,83,84,85,86,87,88,89,90,91,92,93,94,95,96,97,98,99,100,101,102,103,104,105,106,107,108,109] (Supplement S6). The characteristics of the excluded studies [110,111,112,113] are detailed in Supplement S7.

### 3.2. Study Characteristics

An exhaustive overview is provided in Table 1, which includes the characteristics of the studies included in the NMA, published between 2004 and 2021. Most studies were conducted in Türkiye, followed by Italy, Iran, and Canada. The RCTs enrolled between 10 and 112 participants per study, with a mean age of 56.15 ± 3.9 years. The interventions evaluated were as follows: complete decongestive therapy, 11 studies (*n* = 340); usual care (USC), 11 studies (*n* = 202); low-level laser therapy, 5 studies (*n* = 93); compression bandaging plus self-lymphatic drainage, 1 study (*n* = 20); compression bandaging, 3 studies (*n* = 75); yoga, 3 studies (*n* = 44); kinesiology taping, 2 studies (*n* = 38); and the multimodal approach, 2 studies (*n* = 47). The specific interventions reported in the individual studies included complete decongestive therapy/without manual lymphatic drainage (*n* = 29); complete decongestive therapy plus Linfadren (*n* = 25); compression therapy (*n* = 46); modified complete decongestive therapy plus intermittent pneumatic compression (*n* = 56); complete decongestive therapy plus continuous passive motion (*n* = 16); pneumatic compression therapy (*n* = 25); self-administered complete decongestive therapy (*n* = 20); MLD massage plus multi-layered compression bandaging (*n* = 25); the multimodal approach plus a home exercise program (*n* = 13); Pilates (*n* = 30); a bandage (*n* = 21); manual lymphatic drainage/Flexitouch (*n* = 10); a compression garment plus a daily self-administered massage (*n* = 10); aqua lymphatic therapy (*n*= 10); and a home land-based exercise program alone (*n* = 8). The studies not included in the NMA are described in Supplement S8.

### 3.3. Risk of Bias Within Studies

Supplement S9 provides details on the risk of bias of included studies in the NMA for each outcome. The most concerning domains were related to bias arising from the randomization process due to a lack of detail about the process and allocation concealment, as well as bias in measuring the outcome because the outcome assessors were not blinded. Supplement S10 presents information on the risk of bias in the studies that could not be included in the NMA.

### 3.4. Network Geometry

Supplement S11 presents the network plots for the primary and secondary outcomes, with the number of interventions varying from 2 to 12 across outcomes. Most networks included direct evidence for diverse comparisons, but several relied on indirect evidence or a single study when the USC was not included.

### 3.5. Effects of Interventions

**Figure 2:**
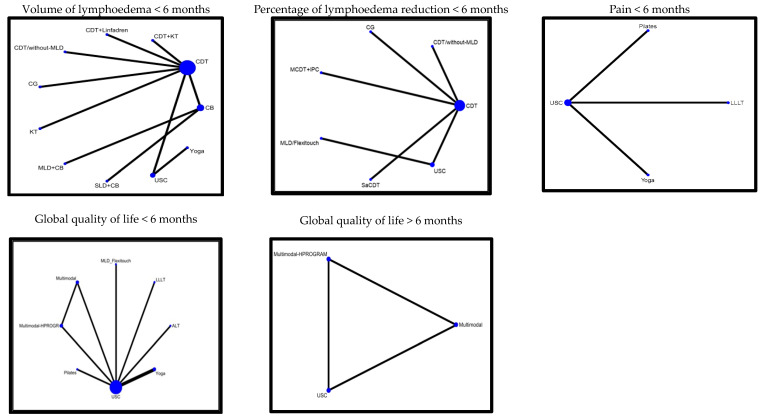
Summary of effects on primary outcomes versus usual care.

**Figure 3:**
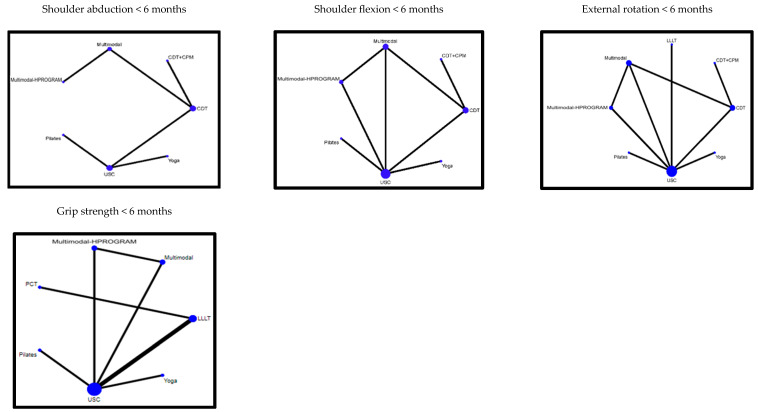
Summary of effects on secondary outcomes versus usual care.

An NMA was conducted for the following primary outcomes: lymphedema volume < 6 months, percent reduction < 6 months, global QoL < 6 months and >6 months, and pain < 6 months (Figure 2). Regarding the secondary outcomes, the NMA included assessments of ROM (abduction, flexion, and external shoulder rotation < 6 months) and grip strength < 6 months (Figure 3). Table 2 provides a summary of the effects compared to the USC, while Supplement S12 reports the estimation of the absolute effect and the certainty of evidence for all the comparisons. The studies not connected to the NMA are described in Supplement S13, where their effect estimates and corresponding certainty of evidence are reported.

#### 3.5.1. Primary Outcomes

Lymphedema volume < 6 months: Ten RCTs [28,29,30,32,33,34,35,39,41,44] were included in the NMA. The effect of all the interventions was uncertain due to the very low certainty of evidence (see Table 2).

Percent reduction < 6 months: Six RCTs [27,28,32,34,37,42] with 387 participants were analyzed in the NMA. Manual lymphatic drainage/Flexitouch was among the least effective interventions compared to USC (MD: −2.0; 95% CI: −7.71 to 3.71; moderate CoE). For the other interventions, the effect was uncertain due to the very low certainty of evidence (see Table 2).

Quality of life < 6 months: Seven RCTs [36,37,38,44,47,50,52] with 203 participants were included in the NMA. Yoga was among the most effective interventions compared to USC (SMD: 1.40; 95% CI: 0.62 to 2.18; moderate CoE). Meanwhile, the multimodal approach plus a home exercise program (SMD: 0.63; 95% CI: −0.23 to 1.49; low CoE), Pilates (SMD: 0.01; 95% CI: −0.48 to 0.52; low CoE), and aqua lymphatic therapy (SMD = −0.60; 95% CI: −1.56 to 0.35; low CoE) may be among the least effective interventions to reduce QoL < 6 months. The effect was uncertain for the rest of the interventions because the CoE was very low (see Table 2).

Pain < 6 months: Three RCTs [43,47,50] with 95 participants were analyzed in the NMA, but the interventions showed an uncertain effect due to the very low CoE.

#### 3.5.2. Secondary Outcomes

ROM shoulder abduction < 6 months: Six RCTs [45,48,49,50,51,52] with 259 participants were included in the NMA. The results show that the multimodal approach may be among the most effective interventions when compared to USC (MD = 11.87; 95% CI: 6.62 to 17.13; low CoE). Interventions such as yoga (MD = −16.74; 95% CI: −28.95 to −4.52) and Pilates (MD = 3.66; 95% CI: −2.81 to 10.15) may be among the least effective interventions compared to USC for this outcome, presenting a low CoE. For the rest of the interventions, the effect was uncertain due to the very low CoE (see Table 2).

ROM shoulder flexion < 6 months: Six RCTs [45,48,49,50,51,52] with 262 participants were analyzed in the NMA. The results show that Pilates may be among the least effective interventions compared to USC (MD = 1.66; 95% CI: −0.80 to 4.14), with a low CoE. The effect of the rest of the interventions was uncertain due to the very low CoE (see Table 2).

ROM external rotation < 6 months: Seven RCTs [45,46,48,49,50,51,52] with 309 participants were analyzed in the NMA. The interventions that showed an intermediate benefit over USC were the multimodal approach (MD = 29.33; 95% CI: 25.64 to 33.03; moderate CoE) and the multimodal approach plus a home exercise program (MD = 27.81; 95% CI: 19.90 to 35.73; moderate CoE). Interventions such as yoga (MD = −9.93; 95% CI: −17.57 to −2.30) and Pilates (MD = 3.0; 95% CI: −1.03 to 7.03) may be among the least effective interventions compared to USC, with a low CoE. For the rest of the interventions, the effect was uncertain due to the very low CoE (see Table 2).

Grip strength < 6 months: Six RCTs [36,40,46,49,50,52] with 235 participants were included in the NMA. The interventions that may be among the least effective interventions compared to USC include yoga (MD = 3.17; 95% CI: −2.97 to 9.31), the multimodal approach (MD = 2.20; 95% CI: −8.48 to 12.88), the multimodal approach plus a home exercise program (MD = 5.00; 95% CI: −6.27 to 16.27), and Pilates (MD = −2.1; 95% CI: −5.02 to 0.82), all with a low CoE. For the rest of the interventions, the effect was uncertain due to the very low CoE (see Table 2).

Adverse effects: Seven studies reported adverse effects. Basoglu (2021) [30] reported an allergic reaction to kinesiology taping that led to the treatment’s discontinuation. Pajero-Otero (2019) [58] stated that 20% of the participants experienced peeling skin with kinesiology taping but not with compression bandages. Pujol-Blaya (2019) [80] documented several adverse effects in the group of adjustable compression: paresthesia (*n* = 3), paresthesia and pain (*n* = 1), pruritus (*n* = 3), removal of the device due to discomfort (*n* = 3), skin problems (*n* = 6), and mobility problems (*n* = 1). In the multi-layered bandages group, there were reports of profuse sweating (*n* = 1), paresthesia (*n* = 1), pain (*n* = 3), pruritus (*n* = 4), and skin problems (*n* = 5). Belmonte (2012) [92] registered a case of erysipelas and an intolerance to the electrode. De Vrieze (2002) [95] reported 20 episodes of erysipelas without significant differences between groups. Torres-Lacomba (2020) [103] reported skin discomfort and irritation that led to the withdrawal of the bandaging in some participants. Szuba (2002) [53] found good tolerance to the addition of an intermittent pneumatic compression pump to manual lymphatic drainage, although a patient experienced a headache and a moderate increase in arterial blood pressure. NMA was not possible for the rest of the primary and secondary outcomes. Supplement S13 shows a pairwise meta-analysis effect estimate and its corresponding certainty of evidence for those outcomes.

### 3.6. Results of Additional Analysis

It was impossible to analyze subgroups using different follow-up periods and QoL questionnaires due to the limited number of studies. A sensitivity analysis was carried out using complete decongestive therapy as the principal comparator in the results where USC did not dominate, given that complete decongestive therapy was the most utilized intervention in the outcomes evaluated (lymphedema volume < 6 months and volume reduction < 6 months). For more details on the NMA graphs, consult Supplement S14. The estimations of the absolute effect and certainty of evidence are found in Supplement S15, and the summary of the compared effect is provided in Supplement S16.

Lymphedema volume < 6 months: Nine RCTs [28,29,30,32,34,35,39,41,44] with 431 participants were included in the NMA. The results show that the combination of complete decongestive therapy + Linfadren was among the most effective interventions (MD: −321.00; 95% CI: −379.64 to −262.35; moderate CoE). USC and the other interventions presented uncertain effects due to the very low CoE (see Supplement S16).

Percent reduction < 6 months: Six RCTs [27,28,32,34,37,42] with 380 participants were included in the NMA. The interventions may be among the least effective interventions when compared to complete decongestive therapy with a compression garment (MD = −1.00; 95% CI: −6.21 to 4.21), USC (MD = −3.09; 95% CI: −8.13 to 1.93), modified complex decongestive therapy + intermittent pneumatic compression pump (MD = 9.4; 95% CI: −4.11 to 22.91), and manual lymphatic drainage/Flexitouch (MD = −5.09; 95% CI: −12.71 to 2.51), all with a low CoE. Self-administered complex decongestive therapy was among the least effective interventions compared to complete decongestive therapy (MD = 6.00; 95% CI: −2.44 to 14.44; moderate CoE). The other interventions presented uncertain effects due to a very low CoE (see Supplement S16).

## 4. Discussion

This SR with NMA included 80 studies, of which only 26, with a total of 1.203 participants, were used to draw conclusions from the NMA. Based on moderate-certainty evidence (CoE), yoga was identified as one of the most effective interventions for improving QoL within 6 months compared to USC (SMD: 1.40; 95% CI: 0.62 to 2.18). Similarly, the multimodal approach and multimodal approach plus a home exercise program showed intermediate benefits in improving external rotation within 6 months (MD: 29.33 and 95% CI: 25.64 to 33.03; MD: 27.81 and 95% CI: 19.90 to 35.73, respectively). For shoulder abduction, the multimodal approach appeared to be among the most effective interventions compared to USC (MD: 11.87; 95% CI: 6.62 to 17.13; low CoE). However, there was no compelling evidence indicating that any PTI outperformed USC for other outcomes. Adverse events were documented with kinesiotaping and compression measures.

The PTI’s heterogeneity was evident, as demonstrated by numerous studies that could not be included in the NMA. This reflects the great diversity of treatments that physiotherapists employ and how these interventions are applied to patients with similar clinical characteristics. This diversity complicated the classification of interventions, making the comparative analysis difficult and highlighting the need for more consensus about the best therapeutic strategy for managing BCRL. Treatments include manual lymphatic drainage, devices such as Flexitouch, exercise programs at home, yoga, Pilates, and aqua lymphatic therapy, each with different action mechanisms, durations, intensities, and modalities. This heterogeneity hindered the analysis and interpretation of results, underlining the urgent need to standardize the PTIs for BCRL. The absence of such standardization hampered the execution of the NMA, and it also makes it challenging to implement evidence-based practices in clinical settings, which can lead to variations in the quality of care patients receive. To overcome this, it is fundamental that future studies include a common comparator, such as USC. This would allow a direct comparison of different interventions, thus facilitating the identification of more effective strategies and improving the consistency of clinical care.

The CoE in this NMA varied by intervention and outcomes. Moderate-certainty evidence was identified for yoga in the improvement of QoL and for manual lymphatic drainage/Flexitouch in the reduction in lymphedema volume, both < 6 months. However, many other interventions, especially concerning lymphedema volume and grip strength, presented low certainty, attributed to the risk of bias in primary studies and to the pooled estimates due to the limited number and size of RCTs included. USC was initially used as the common comparator in the NMA due to its frequent application in clinical studies, serving as a standard for comparing the effectiveness of different PTIs. It was later observed that USC was not always the most used intervention, depending on the outcome analyzed. For example, in the cases of volume reduction < 6 months and percent reduction < 6 months, both USC and complete decongestive therapy were frequently applied in 11 studies each. This highlights the need to select the most appropriate comparator according to the outcome of interest to ensure relevant clinical comparisons. A sensitivity analysis showed that the CoE varied with the comparator used. This indicates that comparator selection is crucial, and using an inadequate common comparator can lead to erroneous conclusions [114,115]. Future NMAs should therefore include a sensitivity analysis with the most frequently used intervention for each outcome.

### 4.1. Implications for Practice

The findings of this NMA suggest that yoga and multimodal programs, including home exercise components, may be considered effective short-term strategies for improving QoL and shoulder mobility in patients with BCRL. However, the overall low-to-very-low certainty of evidence (CoE) across most outcomes indicates that treatment decisions should not rely solely on these results, but rather should be integrated with clinical expertise and patient preferences. In daily practice, intervention choice should also take into account feasibility, cost, and the potential for adverse events, particularly with kinesiotaping and compression measures. Specifically, allergic reactions, irritation, and skin peeling have been reported with kinesiotaping, which may limit its use in patients with dermatosis or ongoing skin treatments. Likewise, compression measures, while effective for reducing limb volume, can cause discomfort, paresthesia, or reduced adherence due to skin problems or restricted mobility. Although these events are generally non-serious, they may compromise treatment continuity; therefore, patient education, close monitoring, and individualized and shared decision-making are recommended to balance potential benefits against risks. Until more robust evidence becomes available, clinicians are encouraged to adopt an individualized approach, prioritizing interventions that combine safety, accessibility, and patient adherence.

### 4.2. Strengths and Limitations of This Study

The main strength of our study is that it is the first to use an NMA to evaluate the effectiveness of different PTIs in treating BCRL, allowing a simultaneous comparison of multiple interventions and a complete comprehension of their effectiveness. While the lack of similar studies limits the possibilities of comparisons to prior research, it also highlights our work’s novelty and added value, establishing a solid base for future research in this area. Previous studies, such as SRs and meta-analyses [10,11,12,14,15,116,117,118,119,120], have reported inconsistent results about the effectiveness of PTIs for BCRL, with variable findings for manual lymphatic drainage, low-level laser therapy, exercise, yoga, and aquatic therapy. These discrepancies are mainly explained by methodological heterogeneity and the low certainty of the evidence. In contrast, our NMA integrates both direct and indirect comparisons, providing a more solid and coherent synthesis to support clinical decision-making. This study followed the guidelines of the Cochrane Handbook for Systematic Reviews of Interventions and those of PRISMA-NMA for its report, guaranteeing the trustworthiness, reproducibility, and transparency of the results. Adherence to these methodologies allows us to present robust and grounded findings that are useful for guiding clinical practice and future research. Applying the GRADE methodology in our analysis is a key strength as it classifies interventions by their effectiveness and CoE, thus guaranteeing transparent and trustworthy recommendations for clinical practice.

One of the main limitations was the large number of studies that could not be connected to the NMA due to the diversity of PTIs and the lack of a standard comparator, limiting our findings’ breadth and applicability. Furthermore, due to the lack of direct comparative studies, the dependence on indirect evidence in many comparisons could have introduced uncertainty into the estimations of effects. Indirect evidence, although useful, offers less certainty than direct evidence, especially when the interventions and clinical contexts vary. The use of USC as a common comparator could have introduced bias, as it was not always the most frequent intervention for the analyzed results, despite the sensitivity analysis. The variability in comparators could also have influenced our conclusions, highlighting the need for more refined criteria to select comparators in future NMAs. Another potential limitation is the restriction of included studies to English, Spanish, and Portuguese, which may have led to the exclusion of relevant evidence published in other languages.

## 5. Conclusions

Yoga and multimodal programs, such as the multimodal approach plus a home exercise program, showed short-term improvements in QoL and external shoulder rotation, supported by a moderate CoE. Regarding lymphedema outcomes, most interventions did not demonstrate compelling superiority over USC in the short term, with the exception of specific combinations, such as complete decongestive therapy plus Linfadren, in limited analyses; the overall certainty was low to very low. For pain, the effects were generally uncertain due to a very low CoE. For grip strength, no intervention demonstrated clear superiority over USC, with a predominantly low-to-very-low CoE.

Given these limitations, management decisions should be guided by clinical expertise and patient preferences while also considering cost, feasibility, and potential adverse events. Future RCTs should employ standardized protocols, include an explicit reference comparator (e.g., USC), recruit larger samples, and extend the follow-up beyond 6 months to assess medium- and long-term outcomes and the durability of benefits. Importantly, the consistent use of standardized comparators in future trials is crucial to ensure better network connectivity. This will allow for more robust and precise estimates of treatment effects and facilitate comprehensive comparisons across multiple interventions.

## Figures and Tables

**Table 1:** Characteristics of included studies.

Author, Year, and Reference	Country	Study Design	N (Female)	BC Stage	Treatment	Type of Surgery	BCRL Stage	Intervention	Sample Size	Age (y) Overall, Mean/Range (SD/SE)	Follow-Up
Ligabue, 2019 [27]	Italy	RCT	41	NR	RT; CT	MT; QDT	NR	-G 1: Self-administered complex decongestive therapy -G 2: Complex decongestive therapy	-G 1: 20 -G 2: 21	56.8 (8.8)	6 weeks
Bergmann, 2014 [28]	Brazil	RCT	57	NR	RT; CT	MT; CE	NR	-G 1: Complete decongestive therapy -G 2: Complete decongestive therapy/without manual lymphatic drainage	-G 1: 28 -G 2: 29	62.8 (10)	6 weeks
Cacchio, 2018 [29]	Italy	RCT	50	NR	RT; CT	MT; QDT	NR	-G 1: Complex decongestive therapy plus Linfadren -G 2: Complex decongestive therapy	-G 1: 25 -G 2: 25	56.2 (2.9) 56.7 (2.5)	3 months
Basoglu, 2021 [30]	Türkiye	RCT	36	NR	NR	BCS; MRM	II	-G 1: Complete decongestive therapy -G 2: Kinesiology taping	-G 1: 19 -G 2: 17	53.5 (5.6)	1 month
Lau, 2009 [31]	China	RCT	21	NR	RT; CT	SM-AC; MRM-AC	NR	-G 1: Low-level laser therapy -G 2: Usual care	-G 1: 11 -G 2: 10	51.1 (8.6)	4 weeks
Sen, 2021 [32]	Türkiye	RCT	54	NR	NR	RM; MM	II; III	-G 1: Complex decongestive therapy -G 2: Usual care	-G 1: 27 -G 2: 27	56.8 (13.7)	4 weeks
McNeely, 2004 [33]	Canada	RCT	50	I; II; III	CT;	RM; MM	NR	-G 1: Manual lymphatic drainage massage plus multi-layered compression bandaging -G 2: Compression bandaging	-G 1: 25 -G 2: 25	58 (13)	4 weeks
Dayes, 2013 [34]	Canada	RCT	103	NR	CT; RT	MT; BCS	NR	-G 1: Complex decongestive therapy -G 2: Compression therapy	-G 1: 57 -G 2: 46	Range: 36 to 86 years	13 months
Bahtiyarca, 2019 [35]	Türkiye	RCT	40	I; II; III	RT; CT; HT	BCS + ALND; MRM + ALND	I; II	-G 1: Compression bandaging plus self-lymphatic drainage -G 2: Compression bandaging	-G 1: 20 -G 2: 20	58.4 (11.69)	6 months
Storz, 2016 [36]	Germany	RCT	40		RT; CT; HT	MRM; BCS	NR	-G 1: Low-level laser therapy -G 2: Usual care	-G 1: 20 -G 2: 20	60.2 (10.1)	3 weeks
Wilburn, 2006 [37]	USA	RCT	10	I; II; III	RT	NR	NR	-G 1: Flexitouch™ -G 2: Compression garment plus daily self-administered massage	-G 1: 10 -G 2: 10	Range: 54 to 78 years	42 days
Letelier, 2014 [38]	Israel	RCT	25	NR	RT; CT	LP; MT	NR	-G 1: Aqua lymphatic therapy -G 2: Home land-based exercise program alone	-G 1: 10 -G 2: 8	55 (6.7)	12 weeks
Tsai, 2009 [39]	Republic of China	RCT	42	NR	RT; CT	RM; MRM; MT	NR	-G 1: Taping -G 2: Bandage	-G 1: 21 -G 2: 21	Range: 36 to 75 years	3 months
Kozanoglu, 2009 [40]	Türkiye	RCT	50	NR	NR	NR	NR	-G 1: Pneumatic compression therapy -G 2: Low-level laser therapy	-G 1: 25 -G 2: 25	48.1 (10.3)	12 months
Gradaslski, 2015 [41]	Poland	RCT	60	NR	NR	NR	II	-G 1: Compression bandaging -G 2: Complex decongestive therapy	-G 1: 30 -G 2: 30	61.7 (9.2)	6 months
Haghighat, 2010 [42]	Iran	RCT	112	I; IIa; IIb	RT; CT	MT	NR	-G 1: Complex decongestive therapy -G 2: Modified complex decongestive therapy plus intermittent pneumatic compression	-G 1: 56 -G 2: 56	53.4 (11.4)	3 weeks
Baxter, 2018 [43]	New Zealand	RCT	16	NR	QT; RT; TH	NR	NR	-G 1: Low-level laser therapy -G 2: Usual care	-G 1: 8 -G 2: 8	61.1 (11)	12 weeks
Pasyar, 2019 [44]	Iran	RCT	40	NR	RT; CT	SND	0; I; II; III	-G 1: Yoga -G 2: Usual care	-G 1: 20 -G 2: 20	51.7 (11.4)	8 weeks
Didem, 2005 [45]	Türkiye	RCT	53	NR	HT + CT+ RT; HT + RT; CT + RT	RM; MRM; LP	NR	-G 1: Complex decongestive therapy -G 2: Usual care	-G 1: 27 -G 2: 26	Range: 31 to 76 years	4 weeks
Omar, 2011 [46]	Egypt	RCT	58	II; III	RT; CT; HT	RM + ALND; MRM + P-ALND; LP	NR	-G 1: Low-level laser therapy -G 2: Usual care	-G 1: 29 -G 2: 29	54.1 (3.5)	16 weeks
Loudon, 2014 [47]	Australia	RCT	23	DCIS; I; II; III	RT; CT	LP; MT	I	-G 1: Yoga -G 2: Usual Care	-G 1: 9 -G 2: 10	57.6 ± 10.5	12 weeks
Park, 2016 [48]	Republic of Korea	RCT	69		CT; RT; HT	SND	I; II; III	-G 1: Multimodal -G 2: Complex decongestive therapy	-G 1: 35 -G 2: 34	53.6 (5.7)	4 weeks
Loudon, 2016 [49]	Australia	RCT	28	DCIS; I; II; III	CT; RT	LP; MT	I	-G 1: Yoga -G 2: Usual care	-G 1: 15 -G 2: 13	60.5 (3.6)	8 weeks
Sener, 2017 [50]	Türkiye	RCT	60	NR	NR	NR	NR	-G 1: Pilates -G 2: Usual care	-G 1: 30 -G 2: 30	53.6 (12.5)	8 weeks
Kizil, 2018 [51]	Türkiye	RCT	32	NR	RT; CT	MRM; ALND	NR	-G 1: Complex decongestive therapy plus continuous passive motion -G 2: Complex decongestive therapy	-G 1: 16 -G 2: 16	Range: 35 to 75 years	2 weeks
Portela, 2008 [52]	Puerto Rico	RCT	44	I; II; III; IV	NR	LP; PM; BLM	NR	-G 1: Multimodal -G 2: Multimodal plus home exercise program -G 2: Usual care	-G 1: 12 -G 2: 13 -G 3: 9	53.5 (16.7)	26 weeks

Abbreviations: BCS: breast-conserving surgery; ALND: axillary lymph node dissection; MRM: modified radical mastectomy; MT: mastectomy; PM: partial mastectomy; BLM: bilateral mastectomy; MM: modified mastectomy; CE: conservative; QDT: quadrantectomy; RM: radical mastectomy; LP: lumpectomy; P-ALND: partial axillary lymph node dissection; SM-AC: simple mastectomy with axillary clearance; MRM-AC: modified radical mastectomy with axillary clearance; SND: sentinel node dissection; RT: radiotherapy; CT: chemotherapy; HT: hormone therapy; RCT: randomized clinical trial; NR: not reported.

**Table 2:** Summary of effects compared to usual standard care.

Intervention.	Volume of Lymphedema <6 Months MD (95% CI)	Percentage of Reduction in Lymphedema <6 Months MD (95% CI)	Global Quality of Life <6 Months SMD (95% CI)	Global Quality of Life >6 Months SMD (95% CI)	Pain <6 Months SMD (95% CI)	Abduction * <6 Months MD (95% CI)	Active Abduction * <6 Months MD (95% CI)	Flexion * <6 Months MD (95% CI)	Active Flexion * MD (95% CI)	Internal Rotation * <6 Months MD (95% CI)	External Rotation * <6 Months MD (95% CI)	Pressure Strength <6 Months MD (95% CI)
Complete decongestive therapy + Linfadren	**−232.31** **(−357.74 to −106.88)**											
Yoga	−10.5 (−325.45 to 304.45)		**1.40 ** **(0.62 to 2.18)**		0.13 (−0.76 to 1.04)	**−16.74** **(−28.95 to −4.5)**		−8.94 (−22.05 to 4.17)		−4.88 (−19.65 to 9.87)	**−9.93** **(−17.57 to −2.3)**	3.17 (−2.97 a 9.31)
Complete decongestive therapy	88.68 (−22.18 to 199.56)	3.10 (−1.93 to 8.13)				−0.40 (−4.50 to 3.70)		−0.30 (−5.13 to 4.52)			−1.18 (−4.46 to 2.09)	
Compression garment	69.68 (−103.14 to 242.52)	2.09 (−5.31 to 9.51)										
Complete decongestive therapy + Kinesiotaping	131.48 (−83.02 to 346.00)											
Kinesiotaping	113.98 (−7.72 to 235.70)											
Complete decongestive therapy/without manual lymphatic drainage	145.85 (−52.91 to 344.63)	9.12 (−1.01 to 19.27)										
Manual lymphatic drainage + Compression garment	294.55 (−22.11 to 611.23)											
Simple lymphatic drainage/self-lymphatic drainage + Compression garment (CG)	**284.55 ** **(89.92 to 479.18**)											
Compression bandaging	**285.55 ** **(91.06 to 480.05)**											
Modified complex decongestive therapy + Intermittent pneumatic compression pump		**12.49 ** **(1.92 to 26.92)**										
Manual lymphatic drainage/Flexitouch		−2.0 (−7.71 to 3.71)	0.05 (−0.82 to 0.93)									
Self-administered complex decongestive therapy		9.09 (−0.86 to 19.06)										
Complete decongestive therapy + Continuous passive motion						8.65 (−13.9 to 31.25)		8.48 (−12.2 to 29.16)			0.10 (−7.86 to 8.07)	
Multimodal			0.18 (−0.68 to 1.05)	18.2 (−5.25 to 41.65)		**11.87 ** **(6.62 to 17.13)**		3.16 (−2.14 to 8.46)			**29.33** **(25.64 to 33.03)**	2.20 (−8.48 to 12.88)
Multimodal training + Home exercise program			0.63 (−0.23 to 1.49)	13.2 (−12.3 to 38.71)		18.47 (−8.59 to 45.55)		8.50 (−0.55 to 17.56)			**27.81** **(19.90 to 35.73**)	5.00 (−6.27 to 16.27)
Pilates			0.01 (−0.48 to 0.52)		−0.16 (−0.67 to 0.33)	3.66 (−2.81 to 10.15)		1.66 (−0.80 to 4.14)			3.0 (−1.03 to 7.03)	−2.1 (−5.02 to 0.82)
Low-level laser therapy			0.17 (−0.62 to 0.96)		0.0 (−0.97 to 0.97)		11.3 (7.85 to 14.74)		**14.09** **(10.00 to 18.19)**		3.0 (−1.03 to 7.03)	2.07 (−0.15 to 4.31)
Pneumatic compression therapy												3.07 (−0.46 to 6.62)
Aqua lymphatic therapy			−0.60 (−1.56 to 0.35)									

* = ROM shoulder.

Among the Most EffectiveIntermediate BenefitAmong the Least EffectiveHigh- or Moderate-Certainty EvidenceBetter than usual care and some alternatives Better than usual care but not better than any of the alternativesNot better than usual careLow-Certainty EvidenceMay be better than usual care and some alternativesMay be better than usual care but not better than any alternativeMay not be better than usual careVery-Low-Certainty EvidenceUncertainty about whether the intervention is better or worse than usual careNo Evidence

Numbers in the colored cells are the estimated mean difference with their 95% confidence interval for each intervention compared to usual standard care; empty cells indicate that there was no evidence for the specific intervention. The bold text represents statistical significance.

## Data Availability

The raw data supporting the conclusions of this article will be made available by the authors on request.

## Supplementary Materials

The following supporting information can be downloaded at: https://www.mdpi.com/article/10.3390/jcm14196762/s1. Supplement S1: PRISMA NMA checklist; Supplement S2: Volumetric definitions and formulae; Supplement S3: NMA inclusion and exclusion criteria; Supplement S4: Classification and description of treatment nodes; Supplement S5: Search strategy used in each database; Supplement S6: Studies not connected to the network meta-analysis; Supplement S7: Characteristics of excluded studies; Supplement S8: Characteristics of studies not connected to the network meta-analysis; Supplement S9: Risk of bias of the included studies; Supplement S10: Risk of bias of studies not included in the NMA; Supplement S11: Network meta-analysis plots; Supplement S12: Absolute effect estimates and certainty of evidence; Supplement S13: Estimators of effect and certainty of evidence for studies not connected to the NMA, by outcome; Supplement S14: Network meta-analysis plots; Supplement S15: Absolute effect estimates and certainty of evidence; Supplement S16: Summary of effects compared to CDT.
