# Supplementary material for: Which Physical Therapy Intervention Is Most Effective in Reducing Secondary Lymphedema Associated with Breast Cancer? A Systematic Review and Network Meta-Analysis
Source: J Clin Med. 2025 Sep 24;14(19):6762. doi: 10.3390/jcm14196762 (PMC12524383; doi:10.3390/jcm14196762)
Supplement: Supplementary file 1 [file jcm-14-06762-s001.zip › jcm-3817399-supplementary.pdf]

Supplemental material

Which physical therapy intervention is most effective in reducing secondary lymphoedema associated with breast cancer? A systematic review and network meta-analysis.

Table of contents

Which physical therapy intervention is most effective in reducing secondary lymphoedema associated with breast cancer? A systematic review and network meta-analysis. 1

Supplementary 3

Supplement S1: PRISMA NMA checklist of items to include when reporting a systematic review involving a network meta-analysis. 3

Supplement S2: Volumetric definitions and formulae. 7

Supplement S3: NMA inclusion and exclusion criteria 9

Supplement S4: Classification and description of treatment nodes. 11

Supplement S5: Search strategy used in each database 14

Supplement S6: Studies not connected to the network meta-analysis. 23

Supplement S7: Characteristics of excluded studies. 26

Supplement S8: Characteristics of studies not connected to the network meta-analysis. 27

Supplement S9: Risk of bias of the included studies. 32

Supplement S10: Risk of bias of studies not included in the NMA. 36

Supplement S11: Network meta-analysis plots. 43

Supplement S12: Absolute effect estimates and certainty of evidence. 45

Supplement S13: Estimators of effect and certainty of evidence for studies not connected to the NMA, by outcome. 48

Supplement S14: Network meta-analysis plots. 56

Supplement S15. Absolute effect estimates and certainty of evidence. 57

Supplement S16. Summary of effects compared to CDT. 58

Supplementary

Supplement S1: PRISMA NMA Checklist of Items to Include When Reporting a Systematic Review Involving a Network Meta-Analysis.

Section/Topic	Item #	Checklist Item	Reported on Page #
**TITLE**			
Title	1	Identify the report as a systematic review *incorporating a network meta-analysis (or related form of meta-analysis).*	*1*
**ABSTRACT**			
Structured summary	2	Provide a structured summary that includes the following, as applicable:<br>**Background:** main objectives<br>**Methods:** data sources; study eligibility criteria, participants, and interventions; study appraisal; and *synthesis methods, such as network meta-analysis.*<br>**Results:** number of studies and participants identified and summary estimates with corresponding confidence/credible intervals; *treatment rankings may also be discussed. Authors may choose to summarize pairwise comparisons against a chosen treatment included in their analyses for brevity.*<br>**Discussion/Conclusions:** limitations, conclusions, and implications of findings.<br>**Other:** primary source of funding; systematic review registration number with registry name.	*3*
**INTRODUCTION**			
Rationale	3	Describe the rationale for the review in the context of what is already known, *including why a network meta-analysis has been conducted.*	*4*
Objectives	4	Provide an explicit statement of questions being addressed, with reference to participants, interventions, comparisons, outcomes, and study design (PICOS).	*4*
**METHODS**			
Protocol and registration	5	Indicate whether a review protocol exists and if and where it can be accessed (e.g., Web address); if available, provide the registration information, including the registration number.	*4*
Eligibility criteria	6	Specify the study characteristics (e.g., PICOS and length of follow-up) and report characteristics (e.g., years considered, language, and publication status) used as criteria for eligibility, giving the rationale. *Clearly*	*5*

		describe eligible treatments included in the treatment network, and note whether any have been clustered or merged into the same node (with justification).	
Information sources	7	Describe all information sources (e.g., databases with dates of coverage, contact with study authors to identify additional studies) in the search and date last searched.	**5**
Search	8	Present a full electronic search strategy for at least one database, including any limits used, such that it could be repeated.	*5*
Study selection	9	State the process for selecting studies (i.e., screening, eligibility, inclusion in systematic review, and, if applicable, inclusion in the meta-analysis).	5
Data collection process	10	Describe the method of data extraction from reports (e.g., piloted forms, independently, and in duplicate) and any processes for obtaining and confirming data from investigators.	6
Data items	11	List and define all variables for which data were sought (e.g., PICOS and funding sources) and any assumptions and simplifications made.	7
**Geometry of the network**	**S1**	Describe methods used to explore the geometry of the treatment network under study and potential biases related to it. This should include how the evidence base has been graphically summarized for presentation and what characteristics were compiled and used to describe the evidence base to readers.	
Risk of bias within individual studies	12	Describe methods used for assessing the risk of bias of individual studies (including specification of whether this was performed at the study or outcome level) and how this information is to be used in any data synthesis.	7
Summary measures	13	State the principal summary measures (e.g., risk ratio and difference in means). *Also, describe the use of additional summary measures assessed, such as treatment rankings and surface under the cumulative ranking curve (SUCRA) values, as well as modified approaches used to present summary findings from meta-analyses.*	7
Planned methods of analysis	14	Describe the methods of handling data and combining results of studies for each network meta-analysis. This should include, but not be limited to, the following:<br>• *Handling of multi-arm trials;*<br>• *Selection of variance structure;*<br>• *Selection of prior distributions in Bayesian analyses;*<br>• *Assessment of model fit.*	7
**Assessment of inconsistency**	**S2**	Describe the statistical methods used to evaluate the agreement of direct and indirect evidence in the treatment network(s) studied. Describe efforts taken to address its presence when found.	7
Risk of bias across studies	15	Specify any assessment of risk of bias that may affect the cumulative evidence (e.g., publication bias and selective reporting within studies).	**7**

Additional analyses	16	Describe methods of additional analyses, if performed, indicating which ones were pre-specified. This may include, but not be limited to, the following:<br>• Sensitivity or subgroup analyses;<br>• Meta-regression analyses;<br>• *Alternative formulations of the treatment network;*<br>• *Use of alternative prior distributions for Bayesian analyses (if applicable).*	*7*
**RESULTS†**			
Study selection	17	Give the number of studies screened, assessed for eligibility, and included in the review, with reasons for exclusions at each stage, ideally with a flow diagram.	8
**Presentation of network structure**	**S3**	Provide a network graph of the included studies to enable visualization of the geometry of the treatment network.	***8***
**Summary of network geometry**	**S4**	Provide a brief overview of the characteristics of the treatment network. This may include a commentary on the abundance of trials and randomized patients for the different interventions and pairwise comparisons in the network, gaps of evidence in the treatment network, and potential biases reflected by the network structure.	***8-9***
Study characteristics	18	For each study, present characteristics for which data were extracted (e.g., study size, PICOS, and follow-up period) and provide the citations.	8-9
Risk of bias within studies	19	Present data on the risk of bias of each study and, if available, any outcome-level assessment.	9
Results of individual studies	20	For all outcomes considered (benefits or harms), present the following for each study: 1) simple summary data for each intervention group and 2) effect estimates and confidence intervals. *Modified approaches may be needed to deal with information from larger networks.*	9
Synthesis of results	21	Present the results of each meta-analysis conducted, including confidence/credible intervals. *In larger networks, authors may focus on comparisons versus a particular comparator (e.g., placebo or standard care), with full findings presented in an appendix. League tables and forest plots may be considered to summarize pairwise comparisons.* If additional summary measures were explored (such as treatment rankings), these should also be presented.	***9-10***
**Exploration for inconsistency**	**S5**	Describe results from investigations of inconsistency. This may include information such as measures of model fit to compare consistency and inconsistency models, *p*-values from statistical tests, or a summary of inconsistency estimates from different parts of the	***9-13***

		treatment network.	
Risk of bias across studies	22	Present the results of any assessment of risk of bias across studies for the evidence base being studied.	Supl 9
Results of additional analyses	23	Give results of additional analyses, if performed (e.g., sensitivity or subgroup analyses, meta-regression analyses*, alternative network geometries studied, alternative choice of prior distributions for Bayesian analyses,* and so forth).	***13-14***
**DISCUSSION**			
Summary of evidence	24	Summarize the main findings, including the strength of evidence for each main outcome; consider their relevance to key groups (e.g., healthcare providers, users, and policymakers).	14
Limitations	25	Discuss the limitations at the study and outcome levels (e.g., risk of bias) and at the review level (e.g., incomplete retrieval of identified research and reporting bias). *Comment on the validity of the assumptions, such as transitivity and consistency. Comment on any concerns regarding the network geometry (e.g., avoidance of certain comparisons).*	15
Conclusions	26	Provide a general interpretation of the results in the context of other evidence and the implications for future research.	16
**FUNDING**			
Funding	27	Describe the sources of funding for the systematic review and other support (e.g., supply of data), as well as the role of funders in the systematic review. This should also include information regarding whether funding has been received from manufacturers of treatments in the network and/or whether some of the authors are content experts with professional conflicts of interest that could affect the use of treatments in the network.	

PICOS = population, intervention, comparators, outcomes, and study design.
* The text in italics indicates wording specific to the reporting of network meta-analyses that has been added for guidance based on the PRISMA statement.
† Authors may wish to plan for the use of appendices to present all the relevant information in full detail for items in this section.

Supplement S2: Volumetric Definitions and Formulae.

**Volumetric Outcome**	**Definition / Explanation**	**Formula**	**Other terms for this outcome in the included studies**
Lymphedema Volume	The excess volume in the limb. The volume is measured in milliliters (mL). It is called ‘lymphedema volume’ because it is the amount of the limb volume that is attributed to lymphedema. Lymphedema volume is NOT the total volume of the limb. To calculate the lymphedema volume, you have to subtract the affected limb from the unaffected limb. In this review, we are interested in the lymphedema volume (or excess volume) that remains in the limb after treatment.	Post-treatment total volume of the affected arm minus post-treatment total volume of the unaffected arm.	**Lymphedema volume**<br><br>has also been called absolute lymphedema volume, post-intervention volume (McNeely 2004), and excess limb volume (Williams 2002).
Volume Reduction	An estimate of how much the limb has been reduced (in ml), presumably from the treatment.	Lymphedema volume at baseline minus the lymphedema volume after treatment.<br><br>OR	**Volume reduction**<br><br>has also been called mean lymphedema volume reduction (Johansson 1998; Johansson 1999) and mean change

		Excess volume before treatment minus the excess volume after treatment.	lymphedema volume (McNeely 2004).
Percent Reduction	The decrease in excess volume relative to the amount of excess volume at baseline. Both the lymphedema volume and the volume reduction are considered absolute values, not relative values. However, when absolute values are used, a person with a large excess limb volume might get a 2% reduction, but the amount can look large because the beginning volume was large. By contrast, a person with a small beginning volume can get a 30% reduction, and it can look small in absolute terms. Thus, it is valuable to think about lymphedema outcomes in a different way, i.e., by looking at the percent change because it is a relative value.	Difference Test A – Difference Test B x 100<br>Difference Test A<br><br>Where the difference is the affected arm volume minus the unaffected arm volume (McNeely 2004).<br><br>Another way to think of percent reduction is this formula:<br><br>Excess volume at baseline – Excess volume post-treatment___________________________________ x 100<br>Excess volume at baseline	**Percent Reduction**<br><br>has also been called percentage lymphedema reduction (Johansson 1998; Johansson 1999), percent change, percent reduction in lymphedema volume (McNeely 2004), and percentage change in excess limb volume (Sitzia 2002).

From: Ezzo J, Manheimer E, McNeely ML, Howell DM, Weiss R, Johansson KI, Bao T, Bily L, Tuppo CM, Williams AF, Karadibak D. Manual lymphatic drainage for lymphedema following breast cancer treatment. Cochrane Database of Systematic Reviews 2015, Issue 5. Art. No.: CD003475. DOI: 10.1002/14651858.CD003475.

Supplement S3: NMA inclusion and exclusion criteria

Study design

- Randomized clinical studies.
- Crossover study

Participants

- Women over the age of 15 with lymphoedema secondary to breast cancer.

Intervention

- Complete decongestive therapy.
- Manual lymphatic drainage.
- Low-level laser therapy.
- Pneumatic pumps.
- Kinesiotaping.
- High-intensity endurance training.
- Moderate-intensity endurance training.
- Low-intensity endurance training.
- Supervised endurance training.
- Unsupervised endurance training.
- Supervised aerobic training.
- Unsupervised aerobic training.
- Endurance training plus aerobic training.
- Endurance training plus water aerobics training.
- Endurance training + aerobic training + stretching.
- Yoga.
- Pilates.
- Shock waves.
- Any combination of these physical therapy interventions.

Comparator

- Normal care
- No exercise/No treatment
- Education

Results of interest

- Reduction in lymphedema volume (lymphedema volume, volume reduction, and percent reduction)
- Quality of life (e.g., European Organisation for Research and Treatment of Cancer Quality of Life Questionnaire (EORTC QLQ-C30) and EORTC QLQ-BR23 questionnaires).
- Pain reduction (e.g., numerical rating scale (NRS) and visual analogue scale (VAS))

- Adverse event from the physical therapy intervention, such as an increase in lymphoedema and pain.
- Range of movement (evaluated with goniometry or another validated method).
- Muscular strength (evaluated with dynamometry or another validated method).

Supplement S4: Classification and Description of Treatment Nodes.

**Classification and Description of Treatment Nodes for BCRL.**	
**Type**	**Definition**
**Intervention**	
Yoga	Exercise training following traditional yoga principles with a physical component (1).
Pilates	Exercise training following traditional Pilates principles, such as centering, concentration, control, precision, flow, and breathing (2)(3).
Aqua lymphatic therapy (ALT)	This approach is based on the recovery exercise principles of Casley Smith (4). The sessions are carried out in a shallow pool with lukewarm water, with a depth of 1.2 to 1.4 meters and a water temperature between 31°C and 33 °C (between 88° F and 91,5° F). The main aim is to maintain or improve the reduction in volume achieved in the intensive treatment phase (5).
Low-level laser therapy (LLLT)	LLLT is a non-invasive form of phototherapy that utilizes wavelengths of light between 650 and 1000 nm to deliver low doses of irradiance to the target tissue (6)(7)(8).
Kinesiotaping (KT)	This therapeutic technique consists of the application of neurofunctional elastic bandages to deal with orthopedic dysfunctions and has been adapted in clinical practice to be used in lymphatic system dysfunctions. Its purpose is to reduce pain or abnormal sensations by providing support for muscular movement, alleviating congestion of lymphatic liquid or subcutaneous hemorrhages, and correcting joint misalignment (9).
Multimodal training	Two or more of the specific types of exercise training mentioned above (not deemed multimodal if only part of warm-up or cool-down) (10).
Multimodal training + Home exercise program (H-EXC-PROGRAM)	Multimodal training (11) in combination with an exercise program done at home.
Complete decongestive therapy (CDT)	Therapeutic technique considered as "the gold standard" for lymphoedema treatment that combines manual lymphatic drainage (DLM), application of compression bandages, myolymphokinetic exercises, skincare, and precautions in everyday activities (12).
Complete decongestive therapy + Kinesiotaping (CDT + KT)	Complete decongestive therapy (CDT) in combination with kinesiotaping (KT).
Complete decongestive therapy + Linfadren (CDT + Linfadren)	Complete decongestive therapy (CDT) in combination with Linfadren.

CDT/without MLD	Complete decongestive therapy (CDT), excluding the component of manual lymphatic drainage (MLD).
Self-administered complex decongestive therapy (SaCDT)	CDT self-administered by the patients, in which they are taught manual lymphatic self-drainage, self-bandaging, breathing exercises, mobilization exercises, muscle-reinforcing exercises, and management of muscle cramps. The physiotherapist teaches the women until they can manage their whole treatment autonomously at home.
Modified complex decongestive therapy (MCDT) + Intermittent pneumatic compression pump (IPC) (MCDT+ IPC).	Complete decongestive therapy (CDT) self-administered by the patients. During this process, patients are taught to carry out manual lymphatic self-drainage, self-bandaging, breathing exercises, mobilization, muscle reinforcement, and muscle cramp management. The physiotherapist provides instructions and guides the women until they acquire the capacity to carry out all the treatment autonomously at home.
Complete decongestive therapy (CDT) + Continuous passive motion (CPM) (CDT + CPM)	CDT in combination with continuous passive motion (CPM). CPM is a specialized device that moves a joint softly and continuously through a range of predefined movements. It is mainly used after surgery or injury to promote healing, prevent joint stiffness, and improve mobility(13).
Manual lymphatic drainage (MLD)	A therapeutic technique that consists of a type of very light cutaneous massage designed to improve lymphatic flow and reabsorption without increasing capillary filtration. This technique follows the methods developed by Földi, Leduc, and Vodder (14)(15).
Manual lymphatic drainage (MLD) + Compression garment (CG) (MLD + CG)	Manual lymphatic drainage in combination with a compression garment (CG).
Compression garment (CG)	A compression bandage is mainly used in the treatment of lymphoedema, whether it is used individually or as part of a therapeutic regime that includes other types of compression. Its main aim is to reduce the accumulation of excess interstitial liquid. Some recommendations for its use include daily use, nighttime use, or when carrying out risky activities, sports, or air travel (16).
Simple lymphatic drainage/self-lymphatic drainage (SLD) + Compression garment (CG)<br><br>(SLD + CG)	MLD self-administered by the patients in combination with a compression garment (CG).
Manual lymphatic drainage /Flexitouch (MLD/Flexitouch)	A system that uses gentle pressure and applies a gentle, dynamic, and variable pressure through the use of garments of inflatable elastic fabric with multiple chambers. The mechanism of action of the Flexitouch device reproduces the techniques employed during MLD (17).

Pneumatic compression therapy (PCT)	A therapeutic technique that employs devices to generate an increase in pressure, thus promoting the flow of liquid from the interstitial space towards the vascular space. These devices can be uni-compartmental or multi-compartmental and generate a pressure gradient that goes from distal to proximal constantly or intermittently (16).
Compression bandaging (CB)	The low-pressure elastic compression bandage is a therapeutic method recommended for use both during activity and rest. This type of elastic bandage is applied by wrapping the extremity in an ascending manner, from the hand to the end of the extremity, with a gradual reduction in pressure (18).
**Comparator**	
Usual care	This technique considers the following:<br>-Instruction in physical exercises to enhance the lymph flow, education in skin care, and safety precautions (normal care).<br>-Educational information and recommendations about lymphoedema.<br>- No intervention provided.

Supplement S5: Search Strategy used in each database.

Search strategy used on Medline:

1 "Breast Cancer Lymphedema"[Mesh]
2 "Breast Cancer Lymphedema"
3 Breast Cancer Treatment-Related Lymphedema
4 or/1-3
5 "Low-Level Light Therapy"[Mesh])
6 "Low-Level Light Therapy"
7 LLLT
8 "Manual Lymphatic Drainage"[Mesh]
9 "Manual Lymphatic Drainage"
10 Intermittent Pneumatic Compression Pump
11 Pneumatic Compression Pump
12 Pneumatic Compression
13 Complete Decongestive Therapy
14 Complex Decongestive Therapy
15 "Athletic Tape"[Mesh]
16 "Athletic Tape"
17 Kinesiotaping
18 Linfotaping
19 "Yoga"[Mesh]
20 "Yoga"
21 "Exercise Movement Techniques"[Mesh]
22 Pilates Training
23 "Extracorporeal Shockwave Therapy"[Mesh]
24 "Extracorporeal Shockwave Therapy"
25 "Exercise Therapy"[Mesh]
26 "Exercise Therapy"
27 "Resistance Training"[Mesh]

28 "Resistance Training"

29 Strength Training

30 Weight-Lifting Exercise Program

31 Weight-Lifting Exercise

32 Aerobic Training

33 Endurance Training

34 "Aquatic Therapy"[Mesh]

35 "Aquatic Therapy"

36 or/5-35

37 Clinical [Title/Abstract]

38 Trial [Title/Abstract]

39 Clinical Trials as Topic [Mesh Terms]

40 Clinical Trial [Publication Type]

41 Random*[Title/Abstract]

42 Random Allocation [Mesh Terms]

43 Therapeutic Use [Mesh Subheading]

44 or/37-43

45 and/4,36, 44

Search strategy used on Lilacs:

1 linfedema del cáncer de mama

2 linfedema posmastectomía

3 linfedema relacionado con el tratamiento del cáncer de mama

4 or/1-3

5 terapia por luz de baja intensidad

6 bioestimulación por láser

7 lllt

8 drenaje línfático manual

9 masaje de drenaje linfático

10 aparatos de compresión neumática intermitente

11 media de compresión neumática

12 terapia descongestiva completa

13 cinta atlética

14 kinesio tape

15 vendaje neuromuscular

16 técnicas de ejercicio con movimiento

17 método pilates

18 ejercicio físico

19 terapia por ejercicio

20 tratamiento con ondas de choque extracorpóreas

21 tratamiento con ondas de choque

22 entrenamiento de fuerza

23 programa de fortalecimiento levantando peso

24 musculación

25 entrenamiento aeróbico

26 entrenamiento de resistencia

27 (balneoterapia)

28 or/5-26

29 and/4,28

30 Filtro: type_of_study:("clinical_trials")

Search strategy used on Medline CINHAL:

1 "Breast cancer lymphedema"

2 "Breast cancer-related lymphedema"

3 "Breast cancer treatment-related lymphedema"

4 or/1-3

5 "Complete decongestive therapy"

6 "Manual lymphatic drainage"

7 "Low-level laser therapy"

8 "Low-level light therapy"

9 "Pneumatic pumps"

10 "Pneumatic compression pumps"

11 Kinesiotaping

12 Linfotaping

13 "High-intensity endurance training"

14 "Moderate-intensity endurance training"

15 "Low-intensity endurance training"

16 "Supervised endurance training"

17 "Unsupervised endurance training"

18 "Supervised aerobic training"

19 "Unsupervised aerobic training"

20 “Weight-lifting exercise”

21 Pilates

22 Yoga

23 “Exercise movement techniques”

24 "Shock waves"

25 "High-energy shock waves"

26"Endurance training"

27 "Aerobic training"

28 "Endurance training"

29 "Water aerobics training"

30"Endurance training"

31 "Aerobic training"<br><br>32 Stretching<br><br>33 or/5-32<br><br>34 “Randomized clinical trial”<br><br>35 and/4,33,34

Search strategy used on Embase:

1 'breast cancer lymphedema'/exp<br><br>2 'breast cancer treatment-related lymphedema'<br><br>3 or/1,2<br><br>4 'low-level light therapy'/exp<br><br>5 lllt:ab,ti<br><br>6 'manual lymphatic drainage'/exp<br><br>7 'intermittent pneumatic compression pump'<br><br>8 'complete decongestive therapy'/exp<br><br>9 'athletic tape'/exp<br><br>10 'kinesiotaping'/exp<br><br>11 linfotaping<br><br>12 'yoga'/exp<br><br>13 'exercise movement techniques'/exp<br><br>14 'pilates training'<br><br>15 'extracorporeal shockwave therapy'/exp<br><br>16 'resistance training'/exp<br><br>17 'weight-lifting exercise'/exp<br><br>18 'aerobic training'/exp<br><br>19 'endurance training'/exp<br><br>20 'exercise therapy'/exp

21 'aquatic therapy'/exp

22 OR/4-21

23 and/3,22

24 Filter: [randomized controlled trial]/lim

Search strategy used on PEDro:

Advanced Search:

Abstract and title: 'breast cancer lymphedema'

Therapy: -

Problem: -

Body part: upper arm, shoulder, or shoulder girdle

Subdiscipline: -

Topic: -

Method: clinical trial

Autor/Association: -

Title only: -

Source: -

Published since: -

New records added since: -

Score at least: -

When searching: Match all search terms (AND)

Search strategy used on CENTRAL:

Search #1

1 "Breast cancer lymphedema"

2 "Breast cancer-related lymphedema"

3 "Breast cancer treatment-related lymphedema"

4 or/1-3

5 "Pneumatic pumps" OR "Pneumatic compression pumps"

6 "Low-level light therapy"

7 "Low-level laser therapy"

8 "Complete decongestive therapy"

9 "Manual lymphatic drainage"

10 or/5-9

11 Randomized clinical trial

12 and/4,10,11

Search #2

1 "Breast cancer lymphedema"

2 "Breast cancer-related lymphedema"

3 "Breast cancer treatment-related lymphedema"

4 or/1-3

5 Kinesiotaping

6 Linfotaping

7 “Weight-lifting”

8 or/5-7

10 and/4,8,9

Search #3

1 "Breast cancer lymphedema"

2 "Breast cancer-related lymphedema"

3 "Breast cancer treatment-related lymphedema"

4 or/1-3

5 "High-intensity endurance training"

6 "Moderate-intensity endurance training"

7 "Low-intensity endurance training"

8 "Supervised endurance training"

9 "Unsupervised endurance training"

10 "Supervised aerobic training"

11 "Unsupervised aerobic training"

12 or/5-11

13 Randomized clinical trial

14 and/4,12,13

Search #4

1 "Breast cancer lymphedema"

2 "Breast cancer-related lymphedema"

3 "Breast cancer treatment-related lymphedema"

4 or/1-3

5 Pilates

6 Yoga

7 "Shock waves"

8 "High-energy shock waves"

9 “Exercise movement techniques”

10 or/5-9

11 Randomized clinical trial

12 and/4,10,11

Search #5

1 "Breast cancer lymphedema"

2 "Breast cancer-related lymphedema"

3 "Breast cancer treatment-related lymphedema"

4 or/1-3

5 "Endurance training"

6 "Aerobic training"

7 "Water aerobics training"

8 Stretching

9 and/5,6

10 and/5,7

11 and/5,6,8

12 or/9,10,11

13 Randomized clinical trial

14 and/12,13

Supplement S6: Studies not connected to the Network Meta-Analysis.

RESULTS

Study selection

Fifty-seven studies could not be integrated because they were not connected to the main network

References:

1. Szuba A, Achalu R, Rockson SG. Decongestive lymphatic therapy for patients with breast carcinoma-associated lymphedema: A randomized, prospective study of a role for adjunctive intermittent pneumatic compression. Cancer. 2002 Dec 1;95(11):2260–7.

2. Cormie P, Pumpa K, Galvão DA, Turner E, Spry N, Saunders C, et al. Is it safe and efficacious for women with lymphedema secondary to breast cancer to lift heavy weights during exercise: A randomised controlled trial. Journal of Cancer Survivorship. 2013 Sep;7(3):413–24.

3. Pop TB, Karczmarek-Borowska B, Tymczak M, Hałas I, Banaś J. The influence of Kinesiology Taping on the reduction of lymphoedema among women after mastectomy - Preliminary study. Wspolczesna Onkologia. 2014;18(2):124–9.

4. Uzkeser H, Karatay S. Intermittent pneumatic compression pump in upper extremity impairments of breast cancer-related lymphedema. Turk J Med Sci. 2013;43(1):99–103.

5. Taradaj J, Halski T, Rosinczuk J, Dymarek R, Laurowski A, Smykla A. The influence of Kinesiology Taping on the volume of lymphoedema and manual dexterity of the upper limb in women after breast cancer treatment. Eur J Cancer Care (Engl). 2016 Jul 1;25(4):647–60.

6. Pajero Otero V, García Delgado E, Martín Cortijo C, Romay Barrero HM, de Carlos Iriarte E, Avendaño-Coy J. Kinesio taping versus compression garments for treating breast cancer–related lymphedema: a randomized, cross-over, controlled trial. Clin Rehabil. 2019 Dec 1;33(12):1887–97.

7. Johansson K, Lie E, Ekdahl C, Lindfeldt J. A RANDOMIZED STUDY COMPARING MANUAL LYMPH DRAINAGE WITH SEQUENTIAL PNEUMATIC COMPRESSION FOR TREATMENT OF POSTOPERATIVE ARM LYMPHEDEMA. Vol. 56, Lymphology. 1998.

8. Omar MTA, Gwada RFM, Omar GSM, EL-Sabagh RM, Mersal AEAE. Low-Intensity Resistance Training and Compression Garment in the Management of Breast Cancer–Related Lymphedema: Single-Blinded Randomized Controlled Trial. Journal of Cancer Education. 2020 Dec 1;35(6):1101–10.

9. Ozsoy-Unubol T, Sanal-Toprak C, Bahar-Ozdemir Y, Akyuz G. EFFICACY OF KINESIO TAPING IN EARLY STAGE BREAST CANCER ASSOCIATED LYMPHEDEMA: A RANDOMIZED SINGLE BLINDED STUDY. Vol. 52, Turkey Lymphology. 2019.

10. Sanal-Toprak C, Ozsoy-Unubol T, Bahar-Ozdemir Y, Akyuz G. THE EFFICACY OF INTERMITTENT PNEUMATIC COMPRESSION ASA SUBSTITUTE FOR MANUAL LYMPHATIC DRAINAGE IN COMPLETE DECONGESTIVE THERAPY IN THE TREATMENT OF BREAST CANCER RELATED LYMPHEDEMA. Lymphology . 2019;52:82–91.

11. Irdesel J, Kahraman Celiktas S. Effectiveness of exercise and compression garments in the treatment of breast cancer related lymphedema. [Turkish]. Turkiye Fiziksel Tip ve Rehabilitasyon Dergisi. 2007;53 (1)(February):16–21.

12. Mcclure RJ, Day R, Brufsky AM, Mcclure MK. Randomized Controlled Trial of the Breast Cancer Recovery Program for Women With Breast Cancer-Related Lymphedema. The American Journal of Occupational Therapy. 2010;64:59–72.

13. Odynets T, Briskin Y, Yefremova A, Goncharenko I. The effectiveness of two individualized physical interventions on the upper limb condition after radical mastectomy. Physiotherapy Quarterly. 2019;27(1):12–7.

14. Johansson K, Hayes S, Speck RM, Schmitz KH. Water-based exercise for patients with chronic arm lymphedema: A randomized controlled pilot trial. Am J Phys Med Rehabil. 2013;92(4):312–9.

15. Ali KM, Gammal ER El, Eladl HM. Effect of Aqua Therapy Exercises on Postmastectomy Lymphedema: A Prospective Randomized Controlled Trial. Ann Rehabil Med. 2021;45(2):131–40.

16. Selcuk Yilmaz S. The randomized controlled study of low-level laser therapy, kinesio-taping and manual lymphatic drainage in patients with stage II breast cancer related lymphedema. 2022;

17. Jeffs E, Wiseman T. Randomised controlled trial to determine the benefit of daily home-based exercise in addition to self-care in the management of breast cancer-related lymphoedema: A feasibility study. Supportive Care in Cancer. 2013 Apr;21(4):1013–23.

18. Basha MA, Aboelnour NH, Alsharidah AS, Kamel FAH. Effect of exercise mode on physical function and quality of life in breast cancer–related lymphedema: a randomized trial. Supportive Care in Cancer. 2022 Mar 1;30(3):2101–10.

19. Ha KJ, Lee SY, Lee H, Choi SJ. Synergistic effects of proprioceptive neuromuscular facilitation and manual lymphatic drainage in patients with mastectomy-related lymphedema. Front Physiol. 2017 Nov 28;8(NOV).

20. Carati CJ, Anderson SN, Gannon BJ, Piller NB. Treatment of postmastectomy lymphedema with low-level laser therapy: A double blind, placebo-controlled trial. Cancer. 2003 Sep 15;98(6):1114–22.

21. Cormie P, Singh B, Hayes S, Peake JM, Galvão DA, Taaffe DR, et al. Acute Inflammatory Response to Low-, Moderate-, and High-Load Resistance Exercise in Women with Breast Cancer-Related Lymphedema. Integr Cancer Ther. 2016 Sep 1;15(3):308–17.

22. Do JH, Kim W, Cho YK, Lee J, Song EJ, Chun YM, et al. EFFECTS OF RESISTANCE EXERCISES AND COMPLEX DECONGESTIVE THERAPY ON ARM FUNCTION AND MUSCULAR STRENGTH IN BREAST CANCER RELATED LYMPHEDEMA. Vol. 184, Lymphology. 2015.

23. Tantawy SA, Abdelbasset WK, Nambi G, Kamel DM. Comparative Study Between the Effects of Kinesio Taping and Pressure Garment on Secondary Upper Extremity Lymphedema and Quality of Life Following Mastectomy: A Randomized Controlled Trial. Integr Cancer Ther. 2019 May 1;18.

24. Lee KW, Kim SB, Lee JH, Kim YS. Effects of Extracorporeal Shockwave Therapy on Improvements in Lymphedema, Quality of Life, and Fibrous Tissue in Breast Cancer-Related Lymphedema. Ann Rehabil Med. 2020 Oct 1;44(5):386–92.

25. Buragadda S, alhuSaini adel, rao melam ganeSwara, arora niSha. Effect of complete decongestive therapy and a home program for patients with post mastectomy lymphedema. J Phys Ther Sci. 2015;27:2743–8.

26. Pekyavaş NÖ, Tunay VB, Akbayrak T, Kaya S, Karataş M. Complex decongestive therapy and taping for patients with postmastectomy lymphedema: A randomized controlled study. European Journal of Oncology Nursing. 2014 Dec 1;18(6):585–90.

27. Damstra RJ, Partsch H. Compression therapy in breast cancer-related lymphedema: A randomized, controlled comparative study of relation between volume and interface pressure changes. J Vasc Surg. 2009 May;49(5):1256–63.

28. Pujol-Blaya V, Salinas-Huertas S, Catasús ML, Pascual T, Belmonte R. Effectiveness of a precast adjustable compression system compared to multilayered compression bandages in the treatment of breast cancer–related lymphoedema: a randomized, single-blind clinical trial. Clin Rehabil. 2019 Apr 1;33(4):631–41.

29. Kaviani A, Fateh M, Yousefi-Nooraie R, Alinagi-Zadeh MR, Ataie-Fashtami L. Low-level laser therapy in management of postmastectomy lymphedema. Lasers Med Sci. 2006 Jul;21(2):90–4.

30. Kilmartin L, Denham T, Fu MR, Yu G, Kuo TT, Axelrod D, et al. Complementary low-level laser therapy for breast cancer-related lymphedema: a pilot, double-blind, randomized, placebo-controlled study. Lasers Med Sci. 2020 Feb 1;35(1):95–105.

31. Muñoz-Alcaraz MN, Jiménez-Vílchez AJ, Santamaría-Peláez M, Pérula-De Torres LA, Olmo-Carmona MV, Muñoz-García MT, et al. Activity-Oriented Antiedema Proprioceptive Therapy (TAPA) for Shoulder Mobility Improvement in Women with Upper Limb Lymphedema Secondary to Breast Cancer: A Multicenter Controlled Clinical Trial. J Clin Med. 2022 Apr 1;11(8).

32. Ridner SH, Poage-Hooper E, Kanar C, Doersam JK, Bond SM, Dietrich MS. A pilot randomized trial evaluating low-level laser therapy as an alternative treatment to manual lymphatic drainage for breast cancer-related lymphedema. Oncol Nurs Forum. 2013 Jul;40(4):383–93.

33. Kilbreath SL, Ward LC, Davis GM, Degnim AC, Hackett DA, Skinner TL, et al. Reduction of breast lymphoedema secondary to breast cancer: a randomised controlled exercise trial. Breast Cancer Res Treat. 2020 Nov 1;184(2):459–67.

34. King M, Deveaux A, White H, Rayson D. Compression garments versus compression bandaging in decongestive lymphatic therapy for breast cancer-related lymphedema: A randomized controlled trial. Supportive Care in Cancer. 2012 May;20(5):1031–6.

35. Tambour M, Holt M, Speyer A, Christensen R, Gram B. Manual lymphatic drainage adds no further volume reduction to Complete Decongestive Therapy on breast cancer-related lymphoedema: a multicentre, randomised, single-blind trial. Br J Cancer. 2018 Nov 13;119(10):1215–22.

36. Uzkeser H, Karatay S, Erdemci B, Koc M, Senel K. Efficacy of manual lymphatic drainage and intermittent pneumatic compression pump use in the treatment of lymphedema after mastectomy: a randomized controlled trial. Breast Cancer. 2015 May 1;22(3):300–7.

37. Jahr S, Schoppe B, Reisshauer A. Effect of treatment with low-intensity and extremely low-frequency electrostatic fields (deep oscillation®) on breast tissue and pain in patients with secondary breast lymphoedema. J Rehabil Med. 2008 Sep;40(8):645–50.

38. A.F. WILLIAMS, A. VADGAMA, P.J. FRANKS, P.S. MORTIMER. A randomized controlled crossover study of manual lymphatic drainage therapy in women with breast cancer-related lymphoedema. Eur J Cancer Care (Engl). 2002;11:254–61.

39. Tastaban E, Soyder A, Aydin E, Sendur OF, Turan Y, Ture M, et al. Role of intermittent pneumatic compression in the treatment of breast cancer–related lymphoedema: a randomized controlled trial. Clin Rehabil. 2020 Feb 1;34(2):220–8.

40. Belmonte R, Tejero M, Ferrer M, Muniesa JM, Duarte E, Cunillera O, et al. Efficacy of low-frequency low-intensity electrotherapy in the treatment of breast cancer-related lymphoedema: A cross-over randomized trial. Clin Rehabil. 2012 Jul;26(7):607–18.

41. Yaman A, Borman P, Inanli A, Kul F, Karahan S. The efficacy of different bandaging methods in patients with breast cancer-related lymphedema: A prospective, randomized study. Vol. 67, Turkish Journal of Physical Medicine and Rehabilitation. Turkish Society of Physical Medicine and Rehabilitation; 2021. p. 155–66.

42. McKenzie DC, Kalda AL. Effect of upper extremity exercise on secondary lymphedema in breast cancer patients: A pilot study. Journal of Clinical Oncology. 2003 Feb 1;21(3):463–6.

43. De Vrieze T, Gebruers N, Nevelsteen I, Fieuws S, Thomis S, De Groef A, et al. Manual lymphatic drainage with or without fluoroscopy guidance did not substantially improve the effect of decongestive lymphatic therapy in people with breast cancer-related lymphoedema (EFforT-BCRL trial): a multicentre randomised trial. J Physiother. 2022 Apr 1;68(2):110–22.

44. Buchan J, Janda M, Box R, Schmitz K, Hayes S. A Randomized Trial on the Effect of Exercise Mode on Breast Cancer-Related Lymphedema. Med Sci Sports Exerc. 2016 Oct 1;48(10):1866–74.

45. Kim DS, Sim YJ, Jeong HJ, Kim GC. Effect of active resistive exercise on breast cancerrelated lymphedema: A randomized controlled trial. Arch Phys Med Rehabil. 2010 Dec;91(12):1844–8.

46. Tidhar D, Katz-Leurer M. Aqua lymphatic therapy in women who suffer from breast cancer treatment-related lymphedema: A randomized controlled study. Supportive Care in Cancer. 2010;18(3):383–92.

47. McNeely ML, Dolgoy ND, Rafn BS, Ghosh S, Ospina PA, Al Onazi MM, et al. Nighttime compression supports improved self-management of breast cancer–related lymphedema: A multicenter randomized controlled trial. Cancer. 2022 Feb 1;128(3):587–96.

48. Fife CE, Davey S, Maus EA, Guilliod R, Mayrovitz HN. A randomized controlled trial comparing two types of pneumatic compression for breast cancer-related lymphedema treatment in the home. Supportive Care in Cancer. 2012 Dec;20(12):3279–86.

49. Malicka I, Rosseger A, Hanuszkiewicz J, Woźniewski M. Kinesiology Taping reduces lymphedema of the upper extremity in women after breast cancer treatment: A pilot study. Przeglad Menopauzalny. 2014;13(4):221–6.

50. Deacon R, de Noronha M, Shanley L, Young K. Does the speed of aquatic therapy exercise alter arm volume in women with breast cancer related lymphoedema? A cross-over randomized controlled trial. Braz J Phys Ther. 2019 Mar 1;23(2):140–7.

51. Torres-Lacomba M, Navarro-Brazález B, Prieto-Gómez V, Ferrandez JC, Bouchet JY, Romay-Barrero H. Effectiveness of four types of bandages and kinesio-tape for treating breast-cancer-related lymphoedema: a randomized, single-blind, clinical trial. Clin Rehabil. 2020 Sep 1;34(9):1230–41.

52. Godoy M de FG, Pereira MR, Oliani AH, de Godoy JMP. Synergic effect of compression therapy and controlled active exercises using a facilitating device in the treatment of arm lymphedema. Int J Med Sci. 2012 May 28;9(4):280–4.

53. Kasseroller RG, Brenner E. A prospective randomised study of alginate-drenched low stretch bandages as an alternative to conventional lymphologic compression bandaging. Supportive Care in Cancer. 2010 Mar;18(3):343–50.

54. Lampinen R, Lee JQ, Leano J, Miaskowski C, Mastick J, Brinker L, et al. Treatment of Breast Cancer–Related Lymphedema Using Negative Pressure Massage: A Pilot Randomized Controlled Trial. Arch Phys Med Rehabil. 2021 Aug 1;102(8):1465-1472.e2.

55. Ridner SH, Murphy B, Deng J, Kidd N, Galford E, Bonner C, et al. A randomized clinical trial comparing advanced pneumatic truncal, chest, and arm treatment to arm treatment only in self-care of arm lymphedema. Breast Cancer Res Treat. 2012 Jan;131(1):147–58.

56. Abdelhalim NM, Samhan AF. Comparison of extracorporeal shock waves therapy versus intermittent pneumatic compression therapy in breast cancer-related lymphedema. Int J Cancer Res. 2018;14(2):77–85.

57. Dhar A, Srivastava A, Pandey RM, Shrestha P, Villet S, Gogia AR. Safety and Efficacy of a Mobiderm Compression Bandage During Intensive Phase of Decongestive Therapy in Patients with Breast Cancer-Related Lymphedema: A Randomized Controlled Trial. Lymphat Res Biol. 2023 Feb 1;21(1):52–9.

. Supplement S7: Characteristics of excluded studies.

Study [ref]	Design	Country	N	Reasons for exclusion
Bertinchamp (72)	Randomized controlled trial	Switzerland	43	Wrong language
Angooti (73)	Quasi-experimental	Iran	36	Wrong study design
Moattari (74)	Non-randomized controlled trial	Iran	21	Wrong study design
Sezgin (75)	Non-randomized controlled trial	Turkey	37	Wrong study design

References:

72. Bertinchamp U, Brossard V, Faouzi M. Comparison between the effectiveness of manual lymph drainage versus Kinesio Taping® on postoperative edema after total knee replacement surgery: A randomized controlled trial. Kinesitherapie. 2021 Jun 1;21(234):22–35.

73. Angooti Oshnari L, Hosseini SA, Haghighat S, Zadeh SH. The effect of complete decongestive therapy on edema volume reduction and pain inwomen with post breast surgery lymph edema. Int J Cancer Manag. 2016 Apr 1;9(2).

74. Moattari M, Jaafari B, Talei A, Piroozi S, Tahmasebi S, Zakeri Z. The Effect of Combined Decongestive Therapy and Pneumatic Compression Pump on Lymphedema Indicators in Patients with Breast Cancer Related Lymphedema. Vol. 14, Iranian Red Crescent Medical Journal Iran Red Crescent Med J. 2012.

75. D SO, Dalyan M, S UD, Duzlu U, CS P, BF K. Complex Decongestive Therapy Enhances Upper Limb Functions in Patients with Breast Cancer-Related Lymphedema. Lymphat Res Biol [Internet]. 2018;16(5):446–52. Available from: https://pubmed.ncbi.nlm.nih.gov/29356592/

Supplement S8: Characteristics of Studies not Connected to the Network Meta-Analysis.

Author, Year	Country	Study Design	N (female)	Stage BC	Treatment	Type of Surgery	BCRL Stage	Intervention	Sample Size	Age (y) Overall, Mean/Range (SD/SE)	Follow-up
Szuba 2002 (19)	USA	RCT	23	NR	RT	ALND	NR	-G1: Intermittent pneumatic compression (30 minutes at 40–50 mmHg)<br>-G2: Decongestive therapy without adjunctive intermittent pneumatic compression.	-G1: 12<br>-G2: 11	66.9 (47–81)	6 months
Cormie 2013 (20)	Australia	RCT	62	I, II, III	RT; CT; HT	MT	NR	-G1: High-load resistance exercise (75-85% 1RM).<br>-G2: Low-load resistance exercise (55-65% 1RM).<br>-G3: Usual care.	-G1: 22<br>-G2: 21<br>-G3: 19	57 (10)	3 months
Pop 2014 (21)	Poland	RCT	44	NR	RT	MT	I; II	-G1: Kinesiology taping.<br>-G2: Traditional taping.	-G1: 22<br>-G2: 22	63	21 days
Uzkeser 2013 (22)	Turkey		25	NR	RT; QT	NR	NR	-G1: Pneumatic compression group.<br>-G2: Pneumatic compression group without intermittent pneumatic compression.	-G1: 12<br>-G2: 13	55 (37-75)	1 month
Taradaj 2016 (24)	Poland	RCT	70	NR	QT; RT; TH.	NR	II; III	-G1: Kinesiology taping + Manual lymphatic drainage.<br>-G2: Quasi-kinesiology taping + Manual lymphatic drainage.<br>-G3: Pneumatic + Manual lymphatic drainage + Multilayer bandaging.	-G1: 22<br>-G2: 23<br>-G3: 25	61.9 (5.1)	1 month
Pajero-Otero 2019 (25)	Spain	RCS	30	NR	RT	LP; MT	II; III	-G1: Kinesiotaping.<br>-G2: Compression garments.	-G1: 30<br>-G2: 30	68.4 (7.1)	1 month
Johansson 1998 (26)	Sweden	RCT	24	NR	RT	PM; MT	NR	-G1: Manual lymphatic drainage.<br>-G2: Sequential pneumatic compression.	-G1: 12<br>-G2: 12	57.5 (47.5 – 69.5)	6 months
Omar 2019 (27)	Egypt	RCT	60	NR	RT; QT; TH.	LP + ALND; MRM; SLNB + ALND.	I; II; III	-G1: Low-intensity resistance exercises.<br>-G2: Exercises and compression garment.	-G1: 30<br>-G2: 30	53.2 (2.99)	4 months
Ozsoy-Unubol 2019 (28)	Turkey	RCT	35	NR	RT; QT	MRM; BCS.	I	-G1: Kinesiotaping.<br>-G2: Compression garment.	-G1: 16<br>-G2: 19	52.5 (7.49)	3 months
Sanal-Toprak 2019 (29)	Turkey	RCT	46	NR	RT; QT	MRM; BCS	II; III	-G1: Intermitent pneumatic compression + Compression bandage.<br>-G2: Manual lymphatic drainage + Compression bandage.	-G1: 22<br>-G2: 24	57.2 (10.30)	3 months
Irdesel 2007 (76)	Turkey	RCT	19	NR	RT	LP + ALND; MRM.	NR	-G1: Exercise.<br>-G2: Exercise + Compression garment.	-G1: 9<br>-G2: 10	51.1 (8.1)	6 months
McClure 2010 (31)	USA	RCT	21	NR	RT; QT	MT; LP	I; II	-G1: Breast Cancer Recovery Program.<br>-G2: Control group.	-G1: 10<br>-G2: 11	58.4 (2.9)	3 months

Odynets 2019 (32)	Ukraine	RCT	68	I; II	NR	MT	I; II; III.	-G1: Water-based exercise.<br>-G2: Pilates.	-G1: 34<br>-G2: 34	57.7 (2.16)	3 months
Johansson 2013 (33)	Australia	RCT	29	NR	RT	NR	I; II; III	-G1: Water-based Exercise.<br>-G2: Control group (continuous exercises).	-G1: 15<br>-G2: 14	63 (56 – 74)	2 months
Ali 2021 (77)	Egypt	RCT	50	I; II	RT; QT	MRM;<br>PM;<br>ALND	I; II	-G1: Aqua therapy resistance exercise program.<br>-G2: Land-based exercise therapy.	-G1: 25<br>-G2: 25	50.6 (9.15)	2 months
Yilmaz 2022 (34)	Turkey	RCT	48	I; II; III	NR	NR	II	-G1: Manual lymphatic drainage.<br>-G2: Kinesiotaping.<br>-G3: Low-level laser therapy.	-G1: 18<br>-G2: 15<br>-G3: 15	54.8 (12.1)	3 months
Jeffs 2013 (35)	England	RCT	23	NR	QT; RT; TH	MT;<br>ALND	NR	-G1: Home-based exercise program and self-care.<br>-G2: Standard lymphoedema self-care.	-G1: 11<br>-G2: 12	65.3 (56.7)	26 weeks
Basha 2022 (36)	Egypt	RCT	60	NR	RT; QT; TH	NR	I; II; III	-G1: Xbox Kinect<br>-G2: Resistance exercise.	-G1: 30<br>-G2: 30	50.5 (7.48)	2 months
Ha 2017 (37)	South Korea	RCT	55	NR	NR	NR	NR	-G1: Proprioceptive neuromuscular facilitation.<br>-G2: Manual lymphatic drainage.<br>-G3: Proprioceptive neuromuscular facilitation + Manual lymphatic drainage.	-G1: 17<br>-G2: 20<br>-G3: 18	52.7 (0.88)	4 months
Carati 2003 (38)	Australia	RCS	61	NR	RT; QT	PM + SM-AC; RM + SM-AC	NR	-G1: Low-level laser therapy.<br>-G2: Low-level laser therapy sham.	-G1: 26<br>-G2: 39	63.5 (35 – 87)	3 months
Cormie 2016 (39)	Australia	RCS	21	I; II; III; IV	RT; QT; HT	ALND	NR	-G1: Low-load resistance exercise.<br>-G2: Moderate-load resistance exercise.<br>-G3: High-load resistance exercise.	-G1: 20<br>-G2: 19<br>-G3: 21	62 (10)	24 hours
Do 2015 (78)	South Korea	RCT	44	I; IIa; IIb; III.	QT; TH	MT; LP	NR	-G1: Complex decongestive therapy.<br>-G2: Complex decongestive therapy + Moderate-intensity resistance exercise program.	-G1: 22<br>-G2: 22	49.7 (10.4)	2 months
Tantawy 2019 (41)	Egypt	RCT	66	NR	QT; RT.	BCS +<br>ALND	I; II	-G1: Kinesiotaping.<br>-G2: Pressure garment.	-G1: 33<br>-G2: 33	54.7 (4.16)	3 weeks
Lee 2020 (42)	Korea	RCT	30	NR	QT; RT.	NR	II	-G1: Extracorporeal shockwave therapy.<br>-G2: Complex physical therapy.	-G1: 15<br>-G2: 15	52.7 (10.9)	3 weeks
Buragadda 2015 (43)	India	RCT	60	I; II	RT; QT	NR	NR	-G1: Conventional treatment.<br>-G2: Complete decongestive therapy.	-G1: 30<br>-G2: 30	56.3 (3.5)	6 weeks
Pekyavas 2014 (44)	Turkey	RCT	45	NR	RT; QT	MRM +<br>ALND;<br>PM: MT	II; III	-G1: Bandage.<br>-G2: Bandage + Kinesiotape.<br>-G3: Kinesiotape.	-G1: 15<br>-G2: 15<br>-G3: 15	49.6 (10.5)	1 month
Damstra 2009 (45)	The Netherlands	RCT	36	NR	RT	PM +<br>ALND;<br>MT +<br>ALND	NR	-G1: Bandages with low interface pressure (20-30 mm Hg).<br>-G2: Bandages exerting high interface pressure (44-58 mm Hg).	-G1: 18<br>-G2: 18	61 (45 – 84)	24 hours
Puyol-Blaya 2019 (46)	Spain	RCT	48	NR	NR	NR	NR	-G1: Precast adjustable compression.<br>-G2: Multilayer compression bandages.	-G1: 24<br>-G2: 24	59.4 (12)	3 months
Kaviani 2006 (47)	Iran	RCT	8	NR	RT	MRM	NR	-G1: Low-level laser therapy.<br>-G2: Low-level laser therapy sham.	-G1: 4<br>-G2: 4	51.2 (12.5)	22 weeks

Kilmartin 2019 (48)	USA	RCT	22	I; II; III	RT; QT	MT; LP	NR	-G1: Low-level laser therapy.<br>-G2: Low-level laser therapy sham.	-G1: 11<br>-G2: 11	61.6 (10.6)	12 months
Muñoz-Alcaraz 2022 (49)	Spain	RCT	63	I; II	NR	MT; BCS	NR	-G1: Activity-oriented antiedema proprioceptive therapy.<br>-G2: Control group	-G1: 31<br>-G2: 32	59.3 (9.6)	3 weeks
Ridner 2013 (50)	USA	RCT	46	NR	RT; QT	NR	I; II; III	-G1: Low-level laser therapy.<br>-G2: Manual lymphatic drainage.<br>-G3: Manual lymphatic drainage + Low-level laser therapy.	-G1: 15<br>-G2: 16<br>-G3: 15	66.6 (10.4)	1 week
Kilbreath 2021 (51)	USA	RCT	89	NR	RT; QT; TH.	NR	NR	-G1: Exercise.<br>-G2: Control.	-G1:<br>-G2:	59.5 (8.0)	3 months
King 2012 (52)	Turkey	RCT	21	NR	RT	MRM; MT; ALND	NR	-G1: Compression glove and sleeve.<br>-G2: Compression bandages.	-G1: 10<br>-G2: 11	61 (44 – 76)	3 months
Tambour 2018 (79)	Denmark	RCT	77	NR	NR	NR	I; II	-G1: Manual lymphatic drainage.<br>-G2: Treatment without manual lymphatic drainage.	-G1: 39<br>-G2: 38	61.5 (11.1)	1 month
Uzkeser 2013 (80)	Turkey	RCT	31	NR	RT; QT	NR	NR	-G1: Complex decongestive physical therapy.<br>-G2: Complex decongestive physical therapy + Intermittent pneumatic compression pump.	-G1: 15<br>-G2: 16	56 (37 – 75)	3 weeks
Jahr 2008 (53)	Germany	RCT	21	NR	RT	NR	NR	-G1: Low-intensity + Extremely-low-frequency electrostatic fields (Deep Oscillation®) + Manual lymphatic drainage.<br>-G2: Manual lymphatic drainage.	-G1: 11<br>-G2: 10	59.2 (41 – 71)	2 months
William 2002 (54)	England	RCS	31	NR	RT; TH.	MT	NR	-G1: Manual lymphatic drainage.<br>-G2: Simple lymphatic drainage.	-G1: 15<br>-G2: 16	60 (2.4)	3 months
Tastaban 2019 (55)	Turkey	RCT	76	NR	NR	NR	I; II	-G1: Complex decongestive therapy.<br>-G2: Complex decongestive treatment + Intermittent pneumatic compression.	-G1: 38<br>-G2: 38	54 (43 – 58)	1 month
Belmonte 2012 (56)	Spain	RCS	36	NR	RT; QT	BCS; MT; SLNB; ALND	NR	-G1: Low-frequency low-intensity electrotherapy + Manual lymphatic drainage.<br>-G2: Manual lymphatic drainage + Low-frequency low-intensity electrotherapy.	-G1: 19<br>-G2: 17	67.8 (11.3)	2 months
Yaman 2021 (57)	Turkey	RCT	60	I; II; III	QT; RT; TH	MRM; LP	NR	-G1: Complex decongestive therapy + Short-stretch multilayer bandaging.<br>-G2: Complex decongestive therapy + 3M Coban bandage.	-G1: 30<br>-G2: 30	54.9 (9.6)	2 months
McKenzie 2003 (58)	Canada	Pilot RCT	14	I; II	NR	NR	NR	-G1: Exercise program.<br>-G2: Control group.	-G1: 7<br>-G2: 7	56.6 (9)	2 months
Vrieze 2022 (59)	Belgium	RCT	194	I; II; III; IV	RT; QT; TH	MTM; BCS	I; II; III	-G1: Fluoroscopy-guided manual lymphatic drainage.<br>-G2: Manual lymphatic drainage.<br>-G3: Placebo manual lymphatic drainage.	-G1: 65<br>-G2: 64<br>-G3: 65	61 (10)	6 months
Buchan 2016 (81)	Australia	RCT	41	NR	RT; QT; TH.	ALND	I; II	-G1: Aerobic-based exercise.<br>-G2: Resistance-based exercise.	-G1: 20<br>-G2: 21	56 (52.8 – 59.2)	3 months

Kim 2010 (60)	Republic of Korea	RCT	40	NR	NR	NR	NR	-G1: Active resistance exercise.<br>-G2: Nonactive resistance exercise.	-G1: 20<br>-G2: 20	51 (10.5)	2 months
Tidhar 2010 (61)	Israel	RCT	28	NR	QT; RT; TH.	LP; MT	NR	-G1: Aqua lymphatic therapy.<br>-G2: Self-care.	-G1: 16<br>-G2: 32	56 (10)	3 months
McNeeely 2022 (62)	Canada	RCT	120	I; II; III	RT; QT; TH.	BCS; MT; ALND	NR	-G1: Daytime compression garment alone.<br>-G2: Daytime compression garment plus nighttime compression bandaging.<br>-G3: Daytime compression garment plus the use of a nighttime compression system garment.	-G1: 39<br>-G2: 44<br>-G3: 37	61 (11)	12 months
Fife 2012 (63)	USA	RCT	36	NR	RT; QT	MT; LP	NR	-G1: Advanced pneumatic compression devices.<br>-G2: Standard pneumatic compression devices.	-G1: 18<br>-G2: 18	60.3 (12)	3 months
Malicka 2014 (82)	Poland	Pilot RCT	28	NR	RT; QT; TH	MT; BCS	I	-G1: Kinesiology taping.<br>-G2: Control group did not receive any antiedema treatments.	-G1: 14<br>-G2: 14	59.8 (6)	1 month
Deacon 2019 (64)	Australia	RCS	18	NR	NR	MT; SLNB	I; II	-G1: Conventional aquatic exercise.<br>-G2: Low-speed aquatic exercise.	-G1: 9<br>-G2: 9	67.3 (52 – 81)	1 hour post-interventio n.
Torres-Lacomba 2020 (65)	Spain	RCT	150	NR	RT; QT; TH.	MM; QDT; LP	I; II	-G1: Multilayer.<br>-G2: Simplified multilayer.<br>-G3: Cohesive.<br>-G4: Adhesive.<br>-G5: Kinesiotaping.	-G1: 30<br>-G2: 30<br>-G3: 30<br>-G4: 30<br>-G5: 30	58.4 (11.4)	3 weeks
Godoy 2012 (66)	Brazil	RCT	20	NR	RT; QT	NR	NR	-G1: Exercising with compression.<br>-G2: Exercising without compression.	-G1: 20<br>-G1: 20	63.3	1 hour
Kasseroller 2010 (67)	Australia	RCT	61	NR	NR	MM; LP	II; III	-G1: Conventional low-stretch compressive bandaging.<br>-G2: Alginate semi-rigid bandage.	-G1: 30<br>-G2: 31	57.4 (9)	22 days
Muñoz-Alcaraz 2022b (49)	Spain	RCT	63	I; II	NR	MT; BCS	NR	-G1: Complex decongestive physical therapy.<br>-G2: Activity-oriented proprioceptive antiedema therapy.	-G1: 32<br>-G2: 31	59.3 (10)	2 weeks
Lampinen 2021 (68)	USA	Pilot RCT	28	NR	RT; QT	MT; BCS	NR	-G1: Negative-pressure massage treatment.<br>-G2: Manual lymphatic drainage.	-G1: 15<br>-G2: 13	62.3 (13)	6 weeks
Ridner 2012 (69)	USA	RCT	42	0/I; II; III; IV	RT; QT.	MT; LP	II	-G1: Truncal/chest/arm compression.<br>-G2: Arm compression only.	-G1: 21<br>-G2: 21	53.8 (9)	1 month
Mohamed 2018 (70)	Egypt	RCT	43	NR	RT; QT.	MT; LP	NR	-G1: Extracorporeal shockwave therapy.<br>-G2: Intermittent pneumatic compression therapy.	-G1: 21<br>-G2: 22	49 (7)	4 weeks
Dhar 2023 (71)	India	RCT	50	NR	RT; QT.	RM + ALND	NR	-G1: Conventional multilayer bandages.<br>-G2: Mobilizing bandaging by using Mobiderm.	-G1: 25<br>-G2: 25	55 (11.1)	15 days

Abbreviations – BCS: Breast-conserving surgery; ALND: Axillar lymph node dissection; MRM: Modified radical mastectomy; MT: Mastectomy; PM: Partial mastectomy; BLM: Bilateral mastectomy; MM: Modified mastectomy; CE: Conservative; QDT: Quadrantectomy; RM: Radical mastectomy; LP: Lumpectomy; P-ALND: Partial axillary lymph node dissection; SM-AC: Simple mastectomy with axillary clearance; MRM-AC: Modified radical mastectomy with axillary clearance; UBS: Unilateral breast surgery; SLNB: Sentinel lymph node biopsy;

SND: Sentinel node dissection; RT: Radiotherapy; CT: Chemotherapy; HT: Hormone therapy; RCT: Randomized clinical trial; RCS: Randomized crossover study; NR: Not reported.

Supplement S9: Risk of Bias of the included studies.

a) Volume of lymphoedema < 6 months:

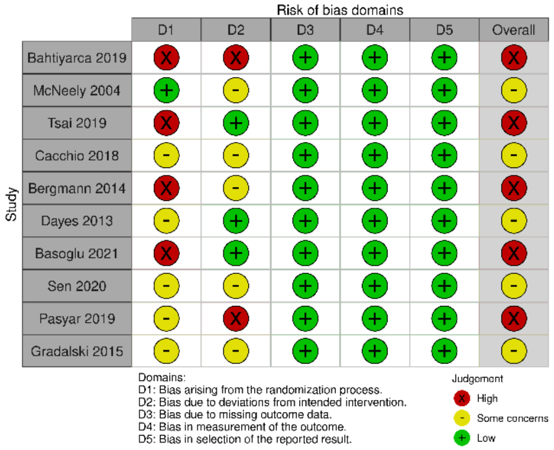

b) Percentage of lymphoedema reduction < 6 months:

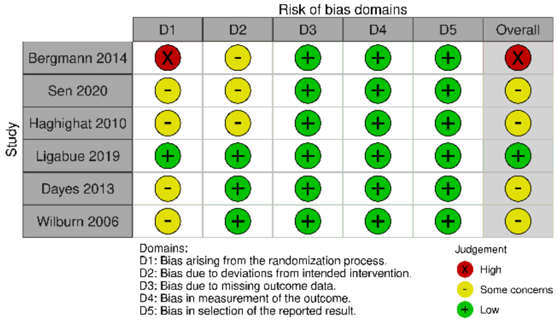

c) Global quality of life < 6 months:

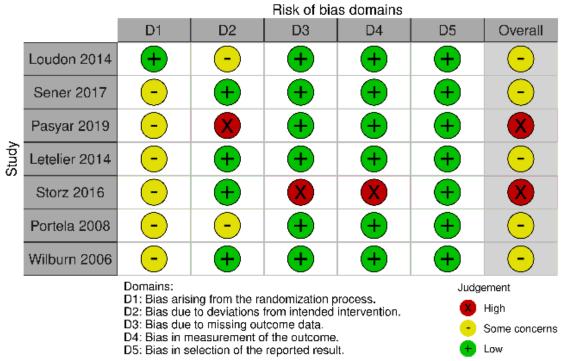

d) Global quality of life > 6 months:

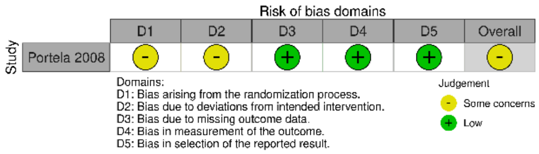

e) Pain < 6 months:

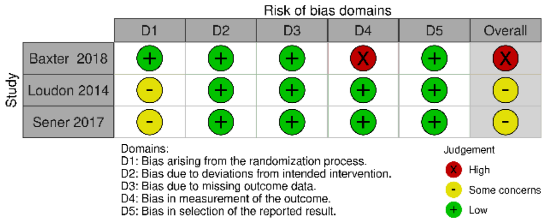

f) Shoulder abduction < 6 months:

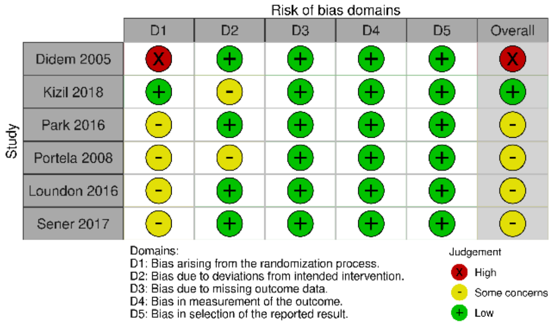

g) Active abduction < 6 months:

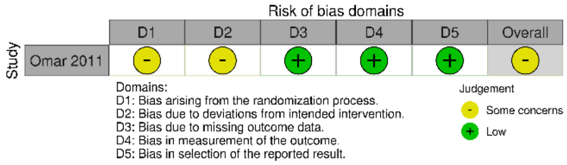

h) Shoulder extension < 6 months:

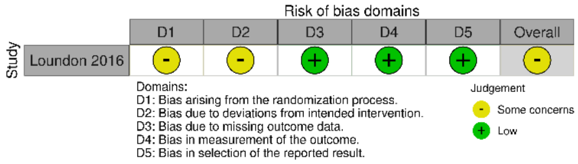

i) **Shoulder flexion < 6 months:**

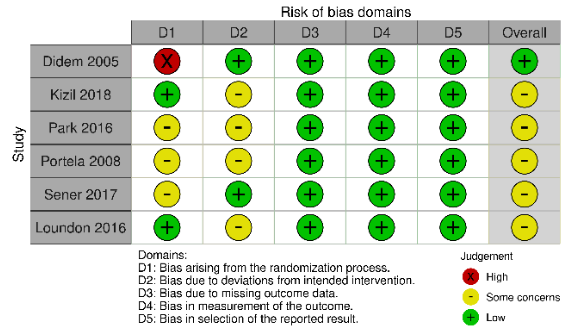

j) **Active shoulder flexion < 6 months:**

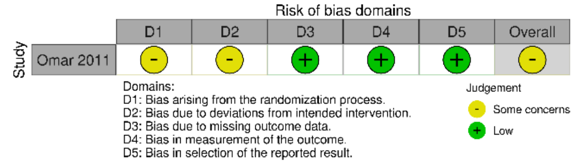

k) **Internal shoulder rotation < 6 months:**

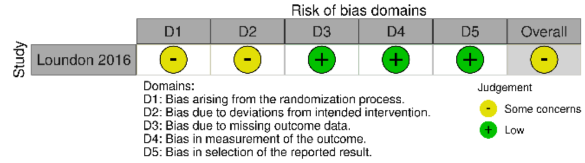

l) **External shoulder rotation < 6 months:**

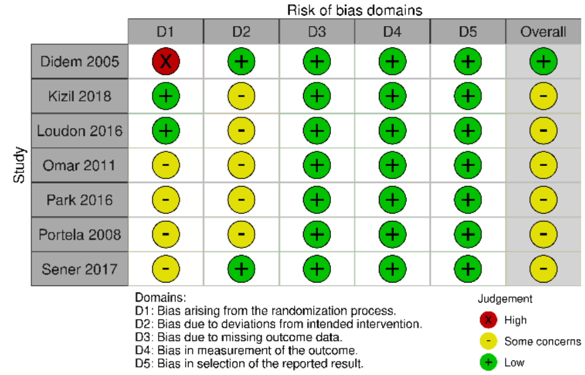

m) **Grip strength < 6 months:**

Risk of bias domains

Study	D1	D2	D3	D4	D5	Overall
Loudon 2016	+	-	+	+	+	-
Sener 2017	-	+	+	+	+	-
Omar 2011	-	-	+	+	+	-
Portela 2008	-	-	+	+	+	-
Storz 2016	-	+	X	X	+	X
Kozanoglu 2019	-	+	+	+	+	-

Domains:
D1: Bias arising from the randomization process.
D2: Bias due to deviations from intended intervention.
D3: Bias due to missing outcome data.
D4: Bias in measurement of the outcome.
D5: Bias in selection of the reported result.

Judgement
X High
- Some concerns
+ Low

n) Adverse effects:

	D1	D2	D3	D4	D5	Overall
Basoglu 2021	-	+	+	+	+	-
Pajero-Otero 2019	+	+	+	+	+	+
Blaya 2019	+	+	+	+	+	+
Belmonte 2012	+	+	+	+	+	+
De Vrieze 2002	+	+	+	+	+	+
Lacomba 2020	+	!	+	+	+	!
Szuba 2002	!	+	+	-	+	-

Domains:
D1: Randomisation process.
D2: Deviations from the intended interventions.
D3: Missing outcome data
D4: Measurement of the outcome
D5: Selection of the reported result

Judgement
+ Low risk
+ Some concerns
- High risk

Supplement S10: Risk of bias of studies not included in the NMA.

a) Volume of lymphoedema < 6 months:

	D1	D2	D3	D4	D5	Overall
Fife 2012	!	+	+	-	+	-
Malicka 2014	!	-	+	+	+	-
Dhar 2023	-	!	+	+	+	-
McNeely 2022	-	!	+	+	+	-
Lampinen 2021	!	+	+	-	+	-
Tambour 2018	+	+	+	+	+	+
Johansson 1998	-	-	+	+	+	-
Lacomba 2020	+	!	+	+	+	!
Jeffs 2013	+	+	+	+	+	+
Storz 2017	!	+	+	+	-	-
Williams 2002	-	!	+	+	+	-
Belmonte 2012	+	+	+	+	+	+
Deacon 2019	!	+	+	+	+	!
Mestre 2017	-	!	+	+	+	-

Domains:
D1: Randomisation process.
D2: Deviations from the intended interventions.
D3: Missing outcome data
D4: Measurement of the outcome
D5: Selection of the reported result

Judgement
+ Low risk
+ Some concerns
- High risk

b) Volume of lymphoedema > 6 months:

	D1	D2	D3	D4	D5	Overall
Bahtiyarca 2019	!	-	+	+	+	-
Gradaslski 2015	!	!	+	+	+	!
McNeeely 2022	-	!	+	+	+	-
Tambour 2018	+	+	+	+	+	+
Jeffs 2013	+	+	+	+	+	+

Domains:
D1: Randomisation process.
D2: Deviations from the intended interventions.
D3: Missing outcome data
D4: Measurement of the outcome
D5: Selection of the reported result

Judgement
+ Low risk
+ Some concerns
- High risk

c) Volume reduction < 6 months:

	D1	D2	D3	D4	D5	Overall
Pekyavas 2014	!	!	+	+	!	!
Dayes 2013	!	+	+	+	+	!
Godoy 2012	!	!	+	+	+	!
Kasseroller 2009	!	!	+	+	+	!
King 2012	!	!	+	+	+	!
McNeely 2004	+	-	-	+	+	-
Damstra 2009	+	+	+	+	+	+
Lau 2009	-	!	+	+	+	-
Wilburn 2006	!	+	+	+	+	!

Domains:
D1: Randomisation process.
D2: Deviations from the intended interventions.
D3: Missing outcome data
D4: Measurement of the outcome
D5: Selection of the reported result

Judgement
+ Low risk
+ Some concerns
- High risk

d) Percentage of reduction < 6 months:

	D1	D2	D3	D4	D5	Overall
Fife 2012	!	+	+	-	+	-
Jeffs 2013	+	+	+	+	+	+
Lacomba 2020	+	!	+	+	+	!
McNeely 2004	+	-	-	+	+	-
Szuba 2002	!	+	+	-	+	-
McNeely 2022	-	!	+	+	+	-
Yaman 2021	!	+	+	+	+	!
Tastaban 2019	!	+	+	!	+	!

Domains:	Judgement	
D1: Randomisation process.	+	Low risk
D2: Deviations from the intended interventions.	+	Some concerns
D3: Missing outcome data	-	High risk
D4: Measurement of the outcome		
D5: Selection of the reported result		

e) Percentage of reduction > 6 months:

	D1	D2	D3	D4	D5	Overall
Jeffs 2013	+	+	+	+	+	+
Ligabue 2019	+	+	+	+	+	+

Domains:	Judgement	
D1: Randomisation process.	+	Low risk
D2: Deviations from the intended interventions.	+	Some concerns
D3: Missing outcome data	-	High risk
D4: Measurement of the outcome		
D5: Selection of the reported result		

f) Quality of life < 6 months:

	D1	D2	D3	D4	D5	Overall
Belmonte 2012	+	+	+	+	+	+
Yaman 2021	!	+	+	+	+	!
Kizil 2018	+	!	+	+	+	!
Yilmaz 2022	!	-	-	-	+	-
Buchan 2016	!	+	+	!	+	!
De Vrieze 2002	+	+	+	+	+	+
Ridner 2013	!	+	+	!	+	!
Basoglu 2021	!	+	+	+	+	!
Tantawy 2019	+	+	+	+	+	+
Do 2015	!	+	+	!	+	!
Mckenzie 2003	!	+	+	-	+	-
Tidhar 2010	+	+	+	!	+	!
McClure 2010	+	!	-	+	+	-
Alcaraz 2022	+	+	+	+	+	+
Tsai 2009	-	+	+	+	+	-
Williams 2002	-	!	+	+	+	-
Jeffs 2013	+	+	+	+	+	+

Domains:	Judgement	
D1: Randomisation process.	+	Low risk
D2: Deviations from the intended interventions.	+	Some concerns
D3: Missing outcome data	-	High risk
D4: Measurement of the outcome		
D5: Selection of the reported result		

g) Quality of life > 6 months:

	D1	D2	D3	D4	D5	Overall
Portela 2008	!	!	+	+	+	!
De Vrieze 2002	+	+	+	+	+	+

Domains:	Judgement	
D1: Randomisation process.	+	Low risk
D2: Deviations from the intended interventions.	+	Some concerns
D3: Missing outcome data	-	High risk
D4: Measurement of the outcome		
D5: Selection of the reported result		

h) Pain < 6 months:

	D1	D2	D3	D4	D5	Overall
Pekyavas 2014	!	!	+	+	!	!
Tambour 2018	+	+	+	+	+	+
Ali 2021	+	+	+	+	+	+
Kozanoglu 2009	+	!	+	-	+	-
Ozsoy 2019	+	+	+	+	+	+
Park 2016	!	+	+	+	!	!
Lee 2020	!	!	+	-	+	-
Letellier 2014	!	+	+	+	+	!
Bergam 2014	-	!	+	+	+	-
Buragadda 2015	!	+	+	+	+	!
Ligabue 2019	+	+	+	+	+	+
Basha 2022	+	+	+	+	+	+
Cormie 2016	!	+	+	-	+	-
Jahr 2008	!	+	+	!	+	!
Tantawy 2019	+	+	+	+	+	+
Uzkeser 2013	!	+	+	+	+	!
Uzkeser 2013a	!	+	+	+	+	!
Irdesel 2007	!	!	+	!	-	-
Ha 2017	!	!	+	!	+	!
Toprak 2019	!	+	+	+	+	!
Pujol-Blaya 2019	+	+	+	+	+	+
Tastaban 2019	!	+	+	+	+	!
Blaya 2019	+	+	+	+	+	+

Domains:	Judgement	
D1: Randomisation process.	+	Low risk
D2: Deviations from the intended interventions.	+	Some concerns
D3: Missing outcome data	-	High risk
D4: Measurement of the outcome		
D5: Selection of the reported result		

i) Pain > 6 months:

	D1	D2	D3	D4	D5	Overall
Kozanoglu 2009	+	!	+	-	+	-
Ligabue 2019	+	+	+	+	+	+
Tambour 2018	+	+	+	+	+	+

Domains:	Judgement	
D1: Randomisation process.	+	Low risk
D2: Deviations from the intended interventions.	+	Some concerns
D3: Missing outcome data	-	High risk
D4: Measurement of the outcome		
D5: Selection of the reported result		

j) Joint range Wrist extension < 6 months:

	D1	D2	D3	D4	D5	Overall
Pajero-Otero 2019	+	+	+	+	+	+
Pop 2014	-	!	+	+	+	-
Uzkeser 2013	!	+	+	+	+	!
Cormie 2013	!	+	+	!	+	!

Domains:	Judgement	
D1: Randomisation process.	+	Low risk
D2: Deviations from the intended interventions.	+	Some concerns
D3: Missing outcome data	-	High risk
D4: Measurement of the outcome		
D5: Selection of the reported result		

k) Joint range Wrist flexion > 6 months:

	D1	D2	D3	D4	D5	Overall
Pop 2014	-	!	+	+	+	-
Uzkeser 2013	!	+	+	+	+	!

Domains:	Judgement	
D1: Randomisation process.	+	Low risk
D2: Deviations from the intended interventions.	+	Some concerns
D3: Missing outcome data	-	High risk
D4: Measurement of the outcome		
D5: Selection of the reported result		

l) Joint range Elbow extension < 6 months:

	D1	D2	D3	D4	D5	Overall
Cormie 2013	!	+	+	!	+	!

Domains:	Judgement	
D1: Randomisation process.	+	Low risk
D2: Deviations from the intended interventions.	+	Some concerns
D3: Missing outcome data	-	High risk
D4: Measurement of the outcome		
D5: Selection of the reported result		

m) Joint range Elbow flexion < 6 months:

	D1	D2	D3	D4	D5	Overall
Cormie 2013	!	+	+	!	+	!
Taradaj 2015	!	+	+	+	+	!
Uzkeser 2013	!	+	+	+	+	!
Pop 2014	-	!	+	+	+	-
Johansson 1998	-	-	+	+	+	-

Domains:	Judgement	
D1: Randomisation process.	+	Low risk
D2: Deviations from the intended interventions.	+	Some concerns
D3: Missing outcome data	-	High risk
D4: Measurement of the outcome		
D5: Selection of the reported result		

n) Joint range shoulder abduction < 6 months:

	D1	D2	D3	D4	D5	Overall
Omar 2019	+	!	+	+	+	!
Ozsoy 2019	+	+	+	+	+	+
Taradaj 2015	!	+	+	+	+	!
Toprak 2019	!	+	+	+	+	!
Odynets 2019	!	+	+	+	+	!
Uzkeser 2013	!	+	+	+	+	!
Cormie 2013	!	+	+	!	+	!
Johansson 2013	!	!	+	+	+	!

Domains:
D1: Randomisation process.
D2: Deviations from the intended interventions.
D3: Missing outcome data
D4: Measurement of the outcome
D5: Selection of the reported result

Judgement
+ Low risk
+ Some concerns
- High risk

o) Joint range shoulder abduction < 6 months:

	D1	D2	D3	D4	D5	Overall
Ozsoy 2019	+	+	+	+	+	+
Park 2016	!	+	+	+	+	!
Toprak 2019	!	+	+	+	+	!
Jeffs 2013	+	+	+	+	+	+

Domains:
D1: Randomisation process.
D2: Deviations from the intended interventions.
D3: Missing outcome data
D4: Measurement of the outcome
D5: Selection of the reported result

Judgement
+ Low risk
+ Some concerns
- High risk

p) Joint range shoulder extension < 6 months:

	D1	D2	D3	D4	D5	Overall
Cormie 2013	!	+	+	!	+	!
Loudon 2016	+	!	+	+	+	!
Unibol-Ozsoy 2019	+	+	+	+	+	+
Park 2016	!	+	+	+	+	!
Toprak 2019	!	+	+	+	+	!
Odynets 2019	!	+	+	+	+	!
Jeffs 2013	+	+	+	+	+	+
Pop 2014	-	!	+	+	+	-

Domains:
D1: Randomisation process.
D2: Deviations from the intended interventions.
D3: Missing outcome data
D4: Measurement of the outcome
D5: Selection of the reported result

Judgement
+ Low risk
+ Some concerns
- High risk

q) Joint range shoulder flexion < 6 months:

	D1	D2	D3	D4	D5	Overall
Cormie 2013	!	+	+	!	+	!
Basha 2022	+	+	+	+	+	+
Unibol-Ozsoy 2019	+	+	+	+	+	+
Taradaj 2015	!	+	+	+	+	!
Odynets 2019	!	+	+	+	+	!
Johansson 2013	!	!	+	+	+	!
Uzkeser 2013	!	+	+	+	+	!
Pajero-Otero 2019	+	+	+	+	+	+
Pop 2014	-	!	+	+	+	-
Ha 2017	!	!	+	!	+	!
Johansson 1998	-	-	+	+	+	-
Carati 2003	!	+	+	+	+	!

Domains:	Judgement	
D1: Randomisation process.	+	Low risk
D2: Deviations from the intended interventions.	+	Some concerns
D3: Missing outcome data	-	High risk
D4: Measurement of the outcome		
D5: Selection of the reported result		

r) Joint range External shoulder rotation < 6 months:

	D1	D2	D3	D4	D5	Overall
Basha 2022	+	+	+	+	+	+
Unibol-Ozsoy 2019	+	+	+	+	+	+
Toprak 2019	!	+	+	+	+	!
Uzkeser 2013	!	+	+	+	+	!
Odynets 2019	!	+	+	+	+	!
Johansson 2013	!	!	+	+	+	!
Johansson 1998	-	-	+	+	+	-

Domains:	Judgement	
D1: Randomisation process.	+	Low risk
D2: Deviations from the intended interventions.	+	Some concerns
D3: Missing outcome data	-	High risk
D4: Measurement of the outcome		
D5: Selection of the reported result		

s) Joint range internal shoulder rotation < 6 months:

	D1	D2	D3	D4	D5	Overall
Unibol-Ozsoy 2019	+	+	+	+	+	+
Toprak 2019	!	+	+	+	+	!
Uzkeser 2013	!	+	+	+	+	!
Loudon 2016	+	!	+	+	+	!
Odynets 2019	!	+	+	+	+	!
Johansson 1998	-	-	+	+	+	-

Domains:	Judgement	
D1: Randomisation process.	+	Low risk
D2: Deviations from the intended interventions.	+	Some concerns
D3: Missing outcome data	-	High risk
D4: Measurement of the outcome		
D5: Selection of the reported result		

t) Grip strength < 6 months:

	D1	D2	D3	D4	D5	Overall
Cormie 2013	!	+	+	!	+	!
Basha 2022	+	+	+	+	+	+
Letellier 2014	!	+	+	+	+	!
Tantawy 2019	+	+	+	+	+	+
Mohamed 2018	+	+	+	+	+	+
Park 2016	!	+	+	+	+	!
Basoglu 2021	!	+	+	+	+	!
Tastaban 2019	!	+	+	+	+	!
Pop 2014	-	!	+	+	+	-
Taradaj 2015	!	+	+	+	+	!
Johansson 1998	-	-	+	+	+	-

Domains:	Judgement	
D1: Randomisation process.	+	Low risk
D2: Deviations from the intended interventions.	+	Some concerns
D3: Missing outcome data	-	High risk
D4: Measurement of the outcome		

u) Grip strength > 6 months:

	D1	D2	D3	D4	D5	Overall
Basoglu 2021	-	+	+	+	+	-
kozanoglu 2009	+	!	+	+	+	!

Domains:	Judgement	
D1: Randomisation process.	+	Low risk
D2: Deviations from the intended interventions.	+	Some concerns
D3: Missing outcome data	-	High risk
D4: Measurement of the outcome		
D5: Selection of the reported result		

Supplement S11: Network meta-analysis plots.

Primary Results:

Figure 2: Summary of effects compared to usual care

Volume of lymphoedema < 6 months

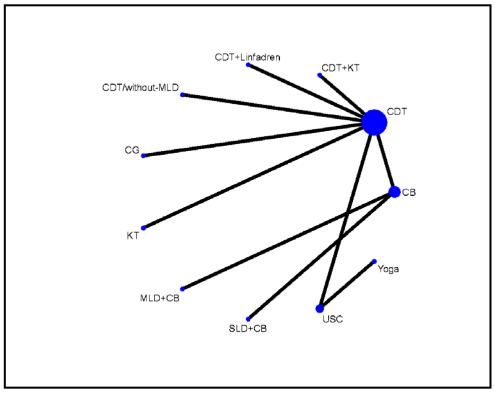

Percentage of lymphoedema reduction < 6 months

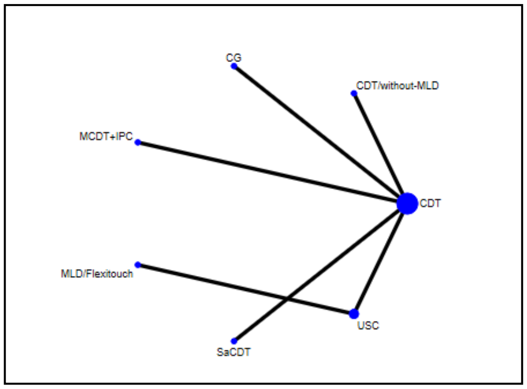

Pain < 6 months

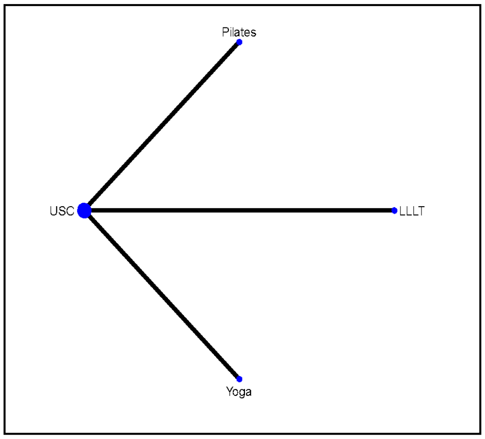

Global quality of life < 6 months

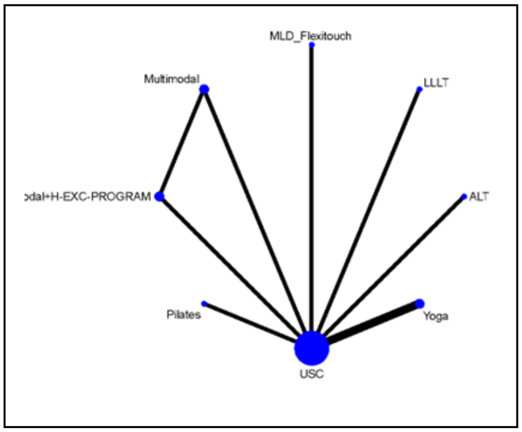

Global quality of life > 6 months

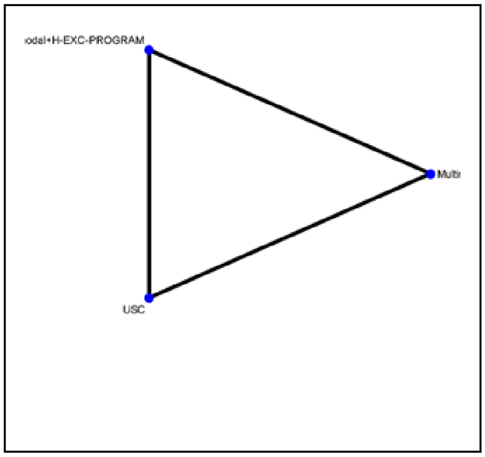

Secondary results:

Shoulder abduction < 6 months

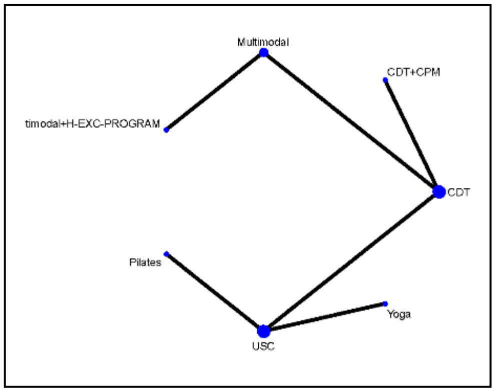

Shoulder flexion < 6 months

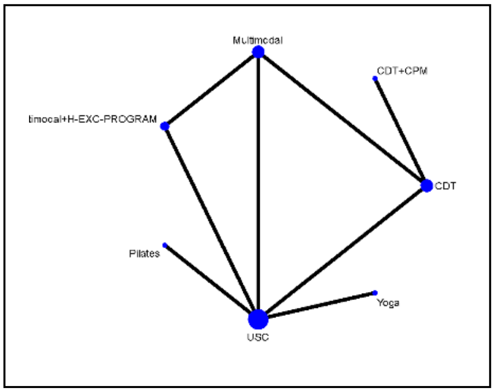

External rotation < 6 months

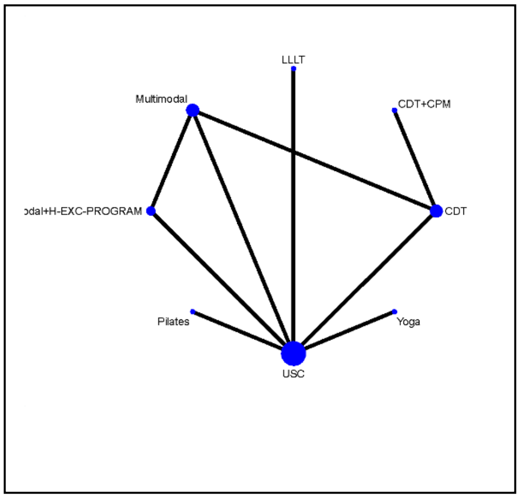

Grip strength < 6 months

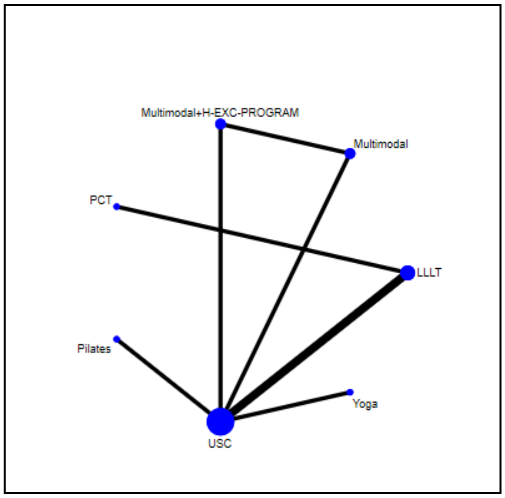

Supplement S12: Absolute effect estimates and certainty of evidence.

8.1. Volume of lymphoedema < 6 months:

COMPARISONS		DIRECT ESTIMATE				INDIRECT ESTIMATE				NETWORK ESTIMATE				
		ABSOLUTE ESTIMATE			GRADE	ABSOLUTE ESTIMATE			GRADE	ABSOLUTE ESTIMATE			GRADE	
TREATMENT 1	TREATMENT 2	Point estimate	CI lower limit	CI upper limit	Final rating	Point estimate	CI lower limit	CI upper limit	Final rating	Point estimate	CI lower limit	CI upper limit	Final rating	**Reason for downgrading**
CB	USC	NA	NA	NA	NA	285.55	91.06	480.05	MODERATE	285.55	91.06	480.05	VERY LOW	RoB, Imprecisionx2
CDT	USC	-88.79	-199.69	22.09	MODERATE	191.00	-5365.03	5747.04	MODERATE	88.68	-22.18	199.56	VERY LOW	RoB, Imprecisionx2
CDT+KT	USC	NA	NA	NA	NA	131.48	-83.02	346.00	LOW	131.48	-83.02	346.00	VERY LOW	RoBx2, Imprecisionx3
CDT+Linfadren	USC	NA	NA	NA	NA	-232,31	-357,74	-106,88	MODERATE	-323,31	-357,74	-106,88	VERY LOW	RoB, Imprecisionx2
CDT/without-MLD	USC	NA	NA	NA	NA	145.85	-52.91	344.63	LOW	145.85	-52.91	344.63	VERY LOW	RoBx2, Imprecisionx3
CG	USC	NA	NA	NA	NA	69.68	-103.14	242.52	MODERATE	69.68	-103.14	242.52	VERY LOW	RoB, Imprecisionx2
KT	USC	NA	NA	NA	NA	113.98	-7.72	235.70	LOW	113.98	-7.72	235.70	VERY LOW	RoBx2, Imprecisionx2
MLD+CB	USC	NA	NA	NA	NA	294.55	-22.11	611.23	MODERATE	294.55	-22.11	611.23	VERY LOW	RoB, Imprecisionx3
SLD+CB	USC	NA	NA	NA	NA	284.55	89.92	479.18	LOW	284.55	89.92	479.18	VERY LOW	RoBx2, Imprecisionx2
Yoga	USC	-10.5	-325.45	304.45	LOW	NA	NA	NA	NA	-10.5	-325.45	304.45	VERY LOW	RoB, Inconsistency, Imprecisionx3
CDT	CB	-197.1	-357.02	-37.17	MODERATE	88.40	-5540.6	5717.41	MODERATE	-196.87	-356.73	-37.00	VERY LOW	RoB, Imprecisionx2
CDT+KT	CB	NA	NA	NA	NA	-154.07	-397.54	89.40	LOW	-154.07	-397.54	89.40	VERY LOW	RoBx2, Imprecisionx2
CDT+Linfadren	CB	NA	NA	NA	NA	-517.87	-688.15	-347.58	LOW	-517.87	-688.15	-347.58	VERY LOW	Imprecisionx2
CDT/without-MLD	CB	NA	NA	NA	NA	-139.70	-369.42	90.02	LOW	-139.70	-369.42	90.02	VERY LOW	Imprecisionx2
CG	CB	NA	NA	NA	NA	-215.87	-423.55	-8.18	MODERATE	-215.87	-423.55	-8.18	VERY LOW	RoB, Imprecisionx2
KT	CB	NA	NA	NA	NA	-171.57	-339.13	-4.00	LOW	-171.57	-339.13	-4.00	VERY LOW	RoBx2, Imprecisionx2
MLD+CB	CB	9.00	-240.91	258.91	MODERATE	-571.52	-506230.3	505087.2	MODERATE	8.99	-240.91	258.91	VERY LOW	RoB, Imprecisionx3
SLD+CB	CB	-1.00	-8.18	6.18	LOW	-571.96	-12144.12	11000.19	LOW	-1.00	-8.18	6.18	VERY LOW	RoBx2, Imprecisionx2
Yoga	CB	NA	NA	NA	NA	-296.05	-666.22	74.10	LOW	-296.05	-666.22	74.10	VERY LOW	RoB, Inconsistency, Imprecisionx3
CDT+KT	CDT	42.79	-140.83	226.43	LOW	-176.33	-365669.9	365317.2	LOW	42.79	-140.83	226.43	VERY LOW	RoBx2, Imprecisionx3
CDT+Linfadren	CDT	-321.0	-379.64	-262.35	MODERATE	-177.37	-145058.8	144704.1	MODERATE	-321	-379.64	-262.35	LOW	RoB, Imprecision
CDT/without-MLD	CDT	57.17	-107.80	222.14	LOW	-178.38	-412912.3	412555.6	LOW	57.16	-107.80	222.14	VERY LOW	RoBx2, Imprecisionx2
CG	CDT	-18.99	-151.58	113.58	MODERATE	161.77	-484713.9	485037.4	MODERATE	-19.00	-151.58	113.58	VERY LOW	RoB, Imprecisionx2
KT	CDT	25.29	-24.90	75.50	LOW	201.27	-95440.18	95842.73	LOW	25.29	-24.90	75.50	VERY LOW	RoBx2, Imprecisionx2
MLD+CB	CDT	NA	NA	NA	NA	205.87	-90.79	502.53	MODERATE	205.87	-90.79	502.53	VERY LOW	RoB, Imprecisionx3
SLD+CB	CDT	NA	NA	NA	NA	195.86	35.84	355.89	LOW	195.86	35.84	355.89	VERY LOW	RoBx2, Imprecisionx2
Yoga	CDT	NA	NA	NA	NA	-99.18	-433.08	234.71	LOW	-99.18	-433.08	234.71	VERY LOW	RoB, Inconsistency, Imprecisionx3
CDT+Linfadren	CDT+KT	NA	NA	NA	NA	-363.79	-556.57	-171.02	MODERATE	-363.79	-556.57	-171.02	VERY LOW	RoB, Imprecisionx2
CDT/without-MLD	CDT+KT	NA	NA	NA	NA	14.37	-232.48	261.22	LOW	14.37	-232.48	261.22	VERY LOW	Imprecisionx2
CG	CDT+KT	NA	NA	NA	NA	-61.79	-288.29	164.69	MODERATE	-61.79	-288.29	164.69	VERY LOW	RoB, Imprecisionx2
KT	CDT+KT	NA	NA	NA	NA	-17.50	-207.87	172.87	LOW	-17.50	-207.87	172.87	VERY LOW	RoBx2, Imprecisionx2
MLD+CB	CDT+KT	NA	NA	NA	NA	163.07	-185.83	511.97	MODERATE	163.07	-185.83	511.97	VERY LOW	RoB, Imprecisionx3
SLD+CB	CDT+KT	NA	NA	NA	NA	153.07	-90.50	396.64	LOW	153.07	-90.50	396.64	VERY LOW	RoBx2, Imprecisionx3
Yoga	CDT+KT	NA	NA	NA	NA	-141.98	-523.05	239.07	LOW	-141.98	-523.05	239.07	VERY LOW	RoB, Inconsistency, Imprecisionx3
CDT/without-MLD vs	CDT+Linfadren	NA	NA	NA	NA	378.16	203.08	553.25	MODERATE	378.16	203.08	553.25	VERY LOW	RoB, Imprecisionx2
CG	CDT+Linfadren	NA	NA	NA	NA	301.99	157.02	446.97	MODERATE	301.99	157.02	446.97	VERY LOW	RoB, Imprecisionx2
KT	CDT+Linfadren	NA	NA	NA	NA	39.38	269.09	423.50	LOW	39.38	269.09	423.50	VERY LOW	RoBx2, Imprecisionx2
MLD+CB	CDT+Linfadren	NA	NA	NA	NA	526.87	224.46	829.27	MODERATE	526.87	224.46	829.27	VERY LOW	RoB, Imprecisionx2
SLD+CB	CDT+Linfadren	NA	NA	NA	NA	516.86	346.43	687.30	LOW	516.86	346.43	687.30	VERY LOW	RoBx2, Imprecisionx2
Yoga	CDT+Linfadren	NA	NA	NA	NA	221.81	-117.20	560.82	LOW	221.81	-117.20	560.82	VERY LOW	RoB, Inconsistency, Imprecisionx2
CG	CDT/without-MLD	NA	NA	NA	NA	-76.16	-287.81	135.47	LOW	-76.16	-287.81	135.47	VERY LOW	RoBx2, Imprecisionx2
KT	CDT/without-MLD	NA	NA	NA	NA	-31.87	-204.31	140.57	LOW	-31.87	-204.31	140.57	VERY LOW	RoBx2, Imprecisionx2
MLD+CB	CDT/without-MLD	NA	NA	NA	NA	148.70	-190.75	488.15	MODERATE	148.70	-190.75	488.15	VERY LOW	RoB, Imprecisionx2
SLD+CB	CDT/without-MLD	NA	NA	NA	NA	138.7	-91.13	368.53	LOW	138.7	-91.13	368.53	VERY LOW	RoBx2, Imprecisionx2
Yoga	CDT/without-MLD	NA	NA	NA	NA	-156.35	-528.79	216.07	LOW	-156.35	-528.79	216.07	VERY LOW	RoBx2, Imprecisionx2
KT	CG	NA	NA	NA	NA	44.29	-97.47	186.07	LOW	44.29	-97.47	186.07	VERY LOW	RoBx2, Imprecisionx2
MLD+CB	CG	NA	NA	NA	NA	224.87	-100.07	549.81	LOW	224.87	-100.07	549.81	VERY LOW	RoBx2, Imprecisionx2
SLD+CB	CG	NA	NA	NA	NA	214.86	07.05	422.68	LOW	214.86	07.05	422.68	VERY LOW	Imprecisionx3
Yoga	CG	NA	NA	NA	NA	-80.18	-439.44	279.07	LOW	-80.18	-439.44	279.07	VERY LOW	RoB, Inconsistency, Imprecisionx3
MLD+CB	KT	NA	NA	NA	NA	180.57	-120.31	481.45	LOW	180.57	-120.31	481.45	VERY LOW	Imprecisionx2
SLD+CB	KT	NA	NA	NA	NA	170.57	2.85	2.85	LOW	170.57	2.85	2.85	LOW	RoBx2
Yoga	KT	NA	NA	NA	NA	-124.48	-462.14	213.16	LOW	-124.48	-462.14	213.16	VERY LOW	Imprecisionx2
SLD+CB	MLD+CB	NA	NA	NA	NA	-10.00	-260.01	240.01	LOW	-10.00	-260.01	240.01	VERY LOW	RoBx2, Imprecisionx2
Yoga	MLD+CB	NA	NA	NA	NA	-305.05	-751.69	141.57	LOW	-305.05	-751.69	141.57	VERY LOW	Imprecisionx2
Yoga	SLD+CB	NA	NA	NA	NA	-295.05	-665.29	75.17	LOW	-295.05	-665.29	75.17	VERY LOW	Imprecisionx2

8.2. Volume reduction < 6 months:

COMPARISONS		DIRECT ESTIMATE				INDIRECT ESTIMATE				NETWORK ESTIMATE				
		ABSOLUTE ESTIMATE			GRADE	ABSOLUTE ESTIMATE			GRADE	ABSOLUTE ESTIMATE			GRADE	
TREATMENT 1	TREATMENT 2	Point estimate	CI lower limit	CI upper limit	Final rating	Point estimate	CI lower limit	CI upper limit	Final rating	Point estimate	CI lower limit	CI upper limit	Final rating	**Reason for downgrading**
LLLT	USC	-126.1	-243,33	-8.87	LOW	NA	NA	NA	NA	NA	NA	NA	NA	NA
MLD/Flexitouch	USC	-260	-377.40	-142.59	MODERATE	NA	NA	NA	NA	NA	NA	NA	NA	NA

8.3. Percentage of lymphoedema reduction < 6 months:

COMPARISONS		DIRECT ESTIMATE				INDIRECT ESTIMATE				NETWORK ESTIMATE				
		ABSOLUTE ESTIMATE			GRADE	ABSOLUTE ESTIMATE			GRADE	ABSOLUTE ESTIMATE			GRADE	
TREATMENT 1	TREATMENT 2	Point estimate	CI lower limit	CI upper limit	Final rating	Point estimate	CI lower limit	CI upper limit	Final rating	Point estimate	CI lower limit	CI upper limit	Final rating	**Reason for downgrading**
CDT	USC	-3.10	-8.13	1.93	VERY LOW	1.85	-5067.33	5071.05	VERY LOW	3.10	-1.93	8.13	VERY LOW	RoBx2, Publication bias, Imprecisionx3
CDT/without-MLD	USC	NA	NA	NA	NA	09.12	´-1.01	19.27	VERY LOW	09.12	´-1.01	19.27	VERY LOW	RoBx2, Publication bias, Imprecisionx2
CG	USC	NA	NA	NA	NA	02.09	-5.31	9.51	VERY LOW	02.09	-5.31	9.51	VERY LOW	RoBx2, Publication bias, Imprecision
MCDT+IPC	USC	NA	NA	NA	NA	12.49	1.92	26.92	VERY LOW	12.49	1.92	26.92	VERY LOW	RoBx2, Publication bias
MLD/Flexitouch	USC	´-2	-7.71	3.71	MODERATE	NA	NA	NA	NA	-2.0	-7.71	3.71	LOW	RoB, Imprecision
SaCDT	USC	NA	NA	NA	NA	09.09	-.86	19.06	VERY LOW	09.09	-.86	19.06	VERY LOW	RoBx2, Publication bias, Imprecision
CDT/without-MLD	CDT	06.03	-2.85	14.91	LOW	-6.30	-15.18	2.57	LOW	06.02	-2.77	14.83	VERY LOW	RoBx2, Imprecisionx2
CG	CDT	-1.00	´-6.21	4.21	MODERATE	-6.30	-18610.61	18598	VERY LOW	-1.00	-6.44	4.44	VERY LOW	RoB, Imprecisionx2
MCDT+IPC	CDT	9.39	-4.13	22.93	MODERATE	-6.11	-19.64	7.42	VERY LOW	9.39	-4.11	22.91	VERY LOW	RoB, Imprecisionx2
MLD/Flexitouch	CDT	NA	NA	NA	NA	-5.09	-12.71	2.51	MODERATE	-5.09	-12.71	2.51	LOW	RoB, Imprecision
SaCDT	CDT	5.99	´-2.88	14.88	HIGH	´-6.05	-14.93	2.83	VERY LOW	5.99	-2.59	14.59	MODERATE	Imprecision
CG	CDT/without-MLD	NA	NA	NA	NA	-7.02	-17.26	3.20	LOW	-7.02	-17.26	3.20	VERY LOW	RoBx2, Imprecision
MLD/Flexitouch	CDT/without-MLD	NA	NA	NA	NA	-11.12	-22.77	0.51	MODERATE	-23	-22.77	0.51	LOW	RoB, Imprecision
SaCDT	CDT/without-MLD	NA	NA	NA	NA	´-.029	-12.33	12.27	LOW	´-.029	-12.33	12.27	VERY LOW	RoBx2, Imprecisionx2
MCDT+IPC	CG	NA	NA	NA	NA	10.4	-4.16	24.96	MODERATE	10.4	-4.16	24.96	VERY LOW	RoB, Imprecisionx2
MLD/Flexitouch	CG	NA	NA	NA	NA	-4.09	-13.32	5.12	MODERATE	-4.09	-13.32	5.12	LOW	RoB, Imprecision
SaCDT	CG	NA	NA	NA	NA	6.99	-3.17	17.17	MODERATE	6.99	-3.17	17.17	VERY LOW	RoB, Imprecisionx2
MLD/Flexitouch	MCDT+IPC	NA	NA	NA	NA	-14.49	-30.01	01.01	VERY LOW	-14.49	-30.01	01.01	VERY LOW	RoB, Imprecisionx2, Intransitivityx2
SaCDT	MCDT+IPC	NA	NA	NA	NA	-3.4	-19.41	12.61	MODERATE	-3.4	-19.41	12.61	VERY LOW	RoB, Imprecisionx2
SaCDT	MLD/Flexitouch	NA	NA	NA	NA	11.09	-0.26	22.46	HIGH	11.09	-0.26	22.46	MODERATE	Imprecision

8.4. Global quality of life < 6 months:

COMPARISONS		DIRECT ESTIMATE				INDIRECT ESTIMATE				NETWORK ESTIMATE				
		ABSOLUTE ESTIMATE			GRADE	ABSOLUTE ESTIMATE			GRADE	ABSOLUTE ESTIMATE			GRADE	
TREATMENT 1	TREATMENT 2	Point estimate	CI lower limit	CI upper limit	Final rating	Point estimate	CI lower limit	CI upper limit	Final rating	Point estimate	CI lower limit	CI upper limit	Final rating	**Reason for downgrading**
ALT	USC	-.60	-1.56	.35	MODERATE	NA	NA	NA	NA	-.60	-1.56	.35	LOW	RoB, Imprecision
LLLT	USC	.17	-.62	.96	MODERATE	NA	NA	NA	NA	.17	-.62	.96	VERY LOW	RoB, Imprecisionx2
MLD_Flexitouch	USC	0.05	-.82	.93	MODERATE	NA	NA	NA	NA	0.05	-.82	.93	VERY LOW	RoB, Imprecisionx2
Multimodal	USC	.18	-.68	01.05	MODERATE	NA	NA	NA	NA	.18	-.68	01.05	VERY LOW	RoB, Imprecisionx2
Multimodal+H-EXC-PROGRAM	USC	.63	-.23	1.49	MODERATE	NA	NA	NA	NA	.63	-.23	1.49	LOW	RoB, Imprecision
Pilates	USC	.01	-.48	.52	MODERATE	NA	NA	NA	NA	.01	-.48	.52	LOW	RoB, Imprecision
Yoga	USC	1.40	.62	2.18	MODERATE	NA	NA	NA	NA	1.40	.62	2.18	MODERATE	RoB
LLLT	ALT	NA	NA	NA	NA	.77	-.46	02.01	MODERATE	.77	-.46	02.01	LOW	RoB, Imprecision
MLD_Flexitouch	ALT	NA	NA	NA	NA	.65	-.63	1.95	MODERATE	.65	-.63	1.95	LOW	RoB, Imprecision
Multimodal	ALT	NA	NA	NA	NA	.79	-.49	02.08	MODERATE	.79	-.49	02.08	LOW	RoB, Imprecision
Multimodal+H-EXC-PROGRAM	ALT	NA	NA	NA	NA	1.23	-.04	2.52	MODERATE	1.23	-.04	2.52	LOW	RoB, Imprecision
Pilates	ALT	NA	NA	NA	NA	.62	-.45	1.70	MODERATE	.62	-.45	1.70	LOW	RoB, Imprecision
Yoga	ALT	NA	NA	NA	NA	2.00	.77	3.24	MODERATE	2.00	.77	3.24	LOW	RoB, Imprecision
MLD_Flexitouch	LLLT	NA	NA	NA	NA	-.11	-1.29	01.06	MODERATE	-.11	-1.29	01.06	LOW	RoB, Imprecision
Multimodal	LLLT	NA	NA	NA	NA	.01	-1.15	1.18	MODERATE	.01	-1.15	1.18	LOW	RoB, Imprecision
Multimodal+H-EXC-PROGRAM	LLLT	NA	NA	NA	NA	.46	-.70	1.63	MODERATE	.46	-.70	1.63	LOW	RoB, Imprecision
Pilates	LLLT	NA	NA	NA	NA	-.15	-1.09	.78	MODERATE	-.15	-1.09	.78	LOW	RoB, Imprecision
Yoga	LLLT	NA	NA	NA	NA	1.23	.12	2.34	MODERATE	1.23	.12	2.34	MODERATE	RoB
Multimodal	MLD_Flexitouch	NA	NA	NA	NA	.13	-1.10	1.36	MODERATE	.13	-1.10	1.36	LOW	RoB, Imprecision
Multimodal+H-EXC-PROGRAM	MLD_Flexitouch	NA	NA	NA	NA	.57	-.65	1.81	MODERATE	.57	-.65	1.81	LOW	RoB, Imprecision
Pilates	MLD_Flexitouch	NA	NA	NA	NA	-.03	-1.04	.97	MODERATE	-.03	-1.04	.97	LOW	RoB, Imprecision
Yoga	MLD_Flexitouch	NA	NA	NA	NA	1.34	.17	2.52	MODERATE	1.34	.17	2.52	MODERATE	RoB
Multimodal+H-EXC-PROGRAM	Multimodal	NA	NA	NA	NA	.44	-.34	1.24	MODERATE	.44	-.34	1.24	LOW	RoB, Imprecision
Pilates	Multimodal	NA	NA	NA	NA	-.16	-1.17	.83	MODERATE	-.16	-1.17	.83	LOW	RoB, Imprecision
Yoga	Multimodal	NA	NA	NA	NA	1.21	.05	2.38	MODERATE	1.21	.05	2.38	MODERATE	RoB
Pilates	Multimodal+H-EXC-PROGRAM	NA	NA	NA	NA	-.61	-1.61	.38	MODERATE	-.61	-1.61	.38	LOW	RoB, Imprecision
Yoga	Multimodal+H-EXC-PROGRAM	NA	NA	NA	NA	.76	-.39	1.93	MODERATE	.76	-.39	1.93	LOW	RoB, Imprecision
Yoga	Pilates	NA	NA	NA	NA	1.38	.45	2.31	MODERATE	1.38	.45	2.31	MODERATE	RoB

8.5. Global quality of life > 6 months:

COMPARISONS		DIRECT ESTIMATE				INDIRECT ESTIMATE				NETWORK ESTIMATE				
		ABSOLUTE ESTIMATE			GRADE	ABSOLUTE ESTIMATE			GRADE	ABSOLUTE ESTIMATE			GRADE	
TREATMENT 1	TREATMENT 2	Point estimate	CI lower limit	CI upper limit	Final rating	Point estimate	CI lower limit	CI upper limit	Final rating	Point estimate	CI lower limit	CI upper limit	Final rating	**Reason for downgrading**
Multimodal	USC	18.2	-5.25	41.65	MODERATE	NA	NA	NA	NA	18.2	-5.25	41.65	VERY LOW	RoB, Imprecisionx2
Multimodal+H-EXC-PROGRAM	USC	13.2	-12.31	38.71	MODERATE	NA	NA	NA	NA	13.2	-12.31	38.71	VERY LOW	RoB, Imprecisionx2
Multimodal+H-EXC-PROGRAM	Multimodal	-5	-27.53	17.53	MODERATE	NA	NA	NA	NA	-5.0	-27.53	17.53	VERY LOW	RoB, Imprecisionx2

8.6. Pain < 6 months:

COMPARISONS		DIRECT ESTIMATE				INDIRECT ESTIMATE				NETWORK ESTIMATE				
		ABSOLUTE ESTIMATE			GRADE	ABSOLUTE ESTIMATE			GRADE	ABSOLUTE ESTIMATE			GRADE	
TREATMENT 1	TREATMENT 2	Point estimate	CI lower limit	CI upper limit	Final rating	Point estimate	CI lower limit	CI upper limit	Final rating	Point estimate	CI lower limit	CI upper limit	Final rating	**Reason for downgrading**
LLLT	USC	0	-.97	.97	LOW	NA	NA	NA	NA	0	-.97	.97	VERY LOW	RoBx2, Imprecisionx2
Pilates	USC	-.16	-.67	.33	MODERATE	NA	NA	NA	NA	-.16	-.67	.33	VERY LOW	RoB, Imprecisionx2
Yoga	USC	.13	-.76	01.04	MODERATE	NA	NA	NA	NA	.13	-.76	01.04	VERY LOW	RoB, Imprecisionx2
Pilates	LLLT	NA	NA	NA	NA	-.16	-1.27	.93	LOW	-.16	-1.27	.93	VERY LOW	RoBx2, Imprecision
Yoga	LLLT	NA	NA	NA	NA	.13	-1.19	1.46	LOW	.13	-1.19	1.46	VERY LOW	RoBx2, Imprecision
Yoga	Pilates	NA	NA	NA	NA	.30	-.72	1.34	MODERATE	.30	-.72	1.34	VERY LOW	RoB, Imprecisionx2

8.7. Shoulder abduction < 6 months:

COMPARISONS		DIRECT ESTIMATE				INDIRECT ESTIMATE				NETWORK ESTIMATE				
		ABSOLUTE ESTIMATE			GRADE	ABSOLUTE ESTIMATE			GRADE	ABSOLUTE ESTIMATE			GRADE	
TREATMENT 1	TREATMENT 2	Point estimate	CI lower limit	CI upper limit	Final rating	Point estimate	CI lower limit	CI upper limit	Final rating	Point estimate	CI lower limit	CI upper limit	Final rating	**Reason for downgrading**
CDT	USC	NA	NA	NA	LOW	6.47	-3359.83	3372.78	LOW	-.40	-4.50	3.70	VERY LOW	RoBx2, Imprecisionx2
CDT+CPM	USC	NA	NA	NA	MODERATE	.19	-22.02	22.41	LOW	8.65	-13.93	31.25	VERY LOW	RoB, Imprecisionx2
Multimodal	USC	NA	NA	NA	NA	11.87	6.62	17.13	LOW	11.87	6.62	17.13	LOW	RoBx2
Multimodal+H-EXC-PROGRAM	USC	NA	NA	NA	NA	18.47	-8.59	45.55	LOW	18.47	-8.59	45.55	VERY LOW	RoBx2, Imprecisionx2
Pilates	USC	NA	NA	NA	MODERATE	NA	NA	NA	NA	3.66	-2.81	10.15	LOW	RoB, Imprecision
Yoga	USC	NA	NA	NA	MODERATE	NA	NA	NA	NA	-16.74	-28.95	-4.52	LOW	RoB, Imprecision
CDT+CPM	CDT	NA	NA	NA	NA	09.05	-13.16	31.28	MODERATE	09.05	-13.16	31.28	LOW	RoB, Imprecision
Multimodal	CDT	NA	NA	NA	MODERATE	.19	-6092.74	6093138	LOW	12.27	8.98	15.57	MODERATE	RoB
Multimodal+H-EXC-PROGRAM	CDT	NA	NA	NA	NA	18.87	-7.87	45.63	MODERATE	18.87	-7.87	45.63	LOW	RoB, Imprecision
Pilates	CDT	NA	NA	NA	NA	04.07	-3.60	11.74	LOW	04.07	-3.60	11.74	VERY LOW	RoBx2, Imprecision
Yoga	CDT	NA	NA	NA	NA	-16.33	-29.22	-3.45	LOW	-16.33	-29.22	-3.45	LOW	RoBx2
Multimodal	CDT+CPM	NA	NA	NA	NA	3.22	11.46	-19.24	MODERATE	3.22	11.46	-19.24	LOW	RoB, Imprecision
Multimodal+H-EXC-PROGRAM	CDT+CPM	NA	NA	NA	NA	9.81	-24.96	44.60	MODERATE	9.81	-24.96	44.60	VERY LOW	RoB, Imprecisionx2
Pilates	CDT+CPM	NA	NA	NA	NA	-4.98	-28.50	18.52	MODERATE	-4.98	-28.50	18.52	VERY LOW	RoB, Imprecisionx2
Yoga	CDT+CPM	NA	NA	NA	NA	-25.39	-51.08	.28	MODERATE	-25.39	-51.08	.28	VERY LOW	RoB, Imprecisionx2
Multimodal+H-EXC-PROGRAM	Multimodal	NA	NA	NA	MODERATE	-24.02	-42423.48	42375.43	MODERATE	6.59	-19.95	33.15	VERY LOW	RoB, Imprecisionx2
Pilates	Multimodal	NA	NA	NA	NA	-8.20	-16.56	.14	LOW	-8.20	-16.56	.14	VERY LOW	RoBx2, Imprecision
Yoga	Multimodal	NA	NA	NA	NA	-28.61	-41.92	-15.31	LOW	-28.61	-41.92	-15.31	VERY LOW	RoBx2, Imprecision
Pilates	Multimodal+H-EXC-PROGRAM	NA	NA	NA	NA	-14.80	-42.64	13.02	MODERATE	-14.80	-42.64	13.02	VERY LOW	RoBx2, Imprecisionx2
Yoga	Multimodal+H-EXC-PROGRAM	NA	NA	NA	NA	-35.21	-64.92	-5.51	MODERATE	-35.21	-64.92	-5.51	VERY LOW	RoBx2, Imprecisionx2
Yoga	Pilates	NA	NA	NA	NA	-20.41	-34.24	-6.57	MODERATE	-20.41	-34.24	-6.57	LOW	RoB, Imprecision

8.8. Shoulder extension < 6 months:

COMPARISONS		DIRECT ESTIMATE				INDIRECT ESTIMATE				NETWORK ESTIMATE				
		ABSOLUTE ESTIMATE			GRADE	ABSOLUTE ESTIMATE			GRADE	ABSOLUTE ESTIMATE			GRADE	
TREATMENT 1	TREATMENT 2	Point estimate	CI lower limit	CI upper limit	Final rating	Point estimate	CI lower limit	CI upper limit	Final rating	Point estimate	CI lower limit	CI upper limit	Final rating	**Reason for downgrading**
Yoga	USC	-11.82	-20.29	-3.34	MODERATE	NA	NA	NA	NA	-11.82	-20.29	-3.34	LOW	RoB, Imprecision

8.9. Shoulder flexion < 6 months:

COMPARISONS		DIRECT ESTIMATE				INDIRECT ESTIMATE				NETWORK ESTIMATE				
		ABSOLUTE ESTIMATE			GRADE	ABSOLUTE ESTIMATE			GRADE	ABSOLUTE ESTIMATE			GRADE	
TREATMENT 1	TREATMENT 2	Point estimate	CI lower limit	CI upper limit	Final rating	Point estimate	CI lower limit	CI upper limit	Final rating	Point estimate	CI lower limit	CI upper limit	Final rating	**Reason for downgrading**
CDT	USC	.15	-4.84	5.14	LOW	2.49	-16.39	21.39	LOW	-.30	-5.13	4.52	VERY LOW	RoBx2, Imprecision
CDT+CPM	USC	NA	NA	NA	NA	8.48	-12.19	29.16	MODERATE	8.48	-12.19	29.16	VERY LOW	RoB, Imprecisionx2
Multimodal	USC	-.99	-19.74	17.74	MODERATE	-3.34	-12.78	06.08	MODERATE	3.16	-2.14	8.46	VERY LOW	RoB, Imprecisionx2
Multimodal+H-EXC-PROGRAM	USC	-7.89	-18.29	2.49	LOW	-12.59	-54.90	29.70	LOW	8.50	-.55	17.56	VERY LOW	RoBx2, Imprecision
Pilates	USC	1.66	-.80	4.14	MODERATE	NA	NA	NA	NA	1.66	-.80	4.14	LOW	RoB, Imprecision
Yoga	USC	-8.94	-22.05	4.17	MODERATE	NA	NA	NA	NA	-8.94	-22.05	4.17	VERY LOW	RoB, Imprecisionx2
CDT+CPM	CDT	8.78	-11.31	28.89	MODERATE	.02	-31619.81	31619.86	MODERATE	8.78	-11.31	28.89	VERY LOW	RoB, Imprecisionx2
Multimodal	CDT	3.49	1.11	5.88	MODERATE	1.15	-18.24	20.54	MODERATE	3.46	01.09	5.83	VERY LOW	RoB, Imprecisionx2
Multimodal+H-EXC-PROGRAM	CDT	NA	NA	NA	NA	8.81	-.80	18.42	LOW	8.81	-.80	18.42	VERY LOW	RoBx2, Imprecision
Pilates	CDT	NA	NA	NA	NA	1.97	-3.45	7.39	MODERATE	1.97	-3.45	7.39	LOW	RoB, Imprecision
Yoga	CDT	NA	NA	NA	NA	-8.63	-22.61	5.34	MODERATE	-8.63	-22.61	5.34	VERY LOW	RoB, Imprecisionx2
Multimodal	CDT+CPM	NA	NA	NA	NA	-5.32	-25.57	14.92	MODERATE	-5.32	-25.57	14.92	VERY LOW	RoB, Imprecisionx2
Multimodal+H-EXC-PROGRAM	CDT+CPM	NA	NA	NA	NA	.02	-22.26	22.30	LOW	.02	-22.26	22.30	VERY LOW	RoBx2, Imprecisionx2
Pilates	CDT+CPM	NA	NA	NA	NA	-6.81	-27.64	14.00	MODERATE	-6.81	-27.64	14.00	VERY LOW	RoB, Imprecisionx2
Yoga	CDT+CPM	NA	NA	NA	NA	-17.42	-41.91	07.06	MODERATE	-17.42	-41.91	07.06	VERY LOW	RoB, Imprecisionx2
Multimodal+H-EXC-PROGRAM	Multimodal	6.89	-9.27	23.07	LOW	2.20	-29.38	33.78	LOW	5.34	-4.37	15.06	VERY LOW	RoBx2, Imprecisionx2
Pilates	Multimodal	NA	NA	NA	NA	-1.49	-7.34	4.36	MODERATE	-1.49	-7.34	4.36	LOW	RoB, Imprecision
Yoga	Multimodal	NA	NA	NA	NA	-12.10	-26.25	02.04	MODERATE	-12.10	-26.25	02.04	LOW	RoB, Imprecision
Pilates	Multimodal+H-EXC-PROGRAM	NA	NA	NA	NA	-6.83	-16.2	2.55	MODERATE	-6.83	-16.2	2.55	LOW	RoB, Imprecision
Yoga	Multimodal+H-EXC-PROGRAM	NA	NA	NA	NA	-17.44	-33.38	-1.50	MODERATE	-17.44	-33.38	-1.50	MODERATE	RoB
Yoga	Pilates	NA	NA	NA	NA	-10.61	-23.96	2.74	MODERATE	-10.61	-23.96	2.74	VERY LOW	RoB, Imprecisionx2

8.12. Internal shoulder rotation < 6 months:

COMPARISONS		DIRECT ESTIMATE				INDIRECT ESTIMATE				NETWORK ESTIMATE				
		ABSOLUTE ESTIMATE			GRADE	ABSOLUTE ESTIMATE			GRADE	ABSOLUTE ESTIMATE			GRADE	
TREATMENT 1	TREATMENT 2	Point estimate	CI lower limit	CI upper limit	Final rating	Point estimate	CI lower limit	CI upper limit	Final rating	Point estimate	CI lower limit	CI upper limit	Final rating	**Reason for downgrading**
Yoga	USC	-4.88	-19.65	9.87	LOW	NA	NA	NA	NA	-4.88	-19.65	9.87	VERY LOW	RoBx2, Imprecisionx2

8.13. External shoulder rotation < 6 months:

COMPARISONS		DIRECT ESTIMATE				INDIRECT ESTIMATE				NETWORK ESTIMATE				
		ABSOLUTE ESTIMATE			GRADE	ABSOLUTE ESTIMATE			GRADE	ABSOLUTE ESTIMATE			GRADE	
TREATMENT 1	TREATMENT 2	Point estimate	CI lower limit	CI upper limit	Final rating	Point estimate	CI lower limit	CI upper limit	Final rating	Point estimate	CI lower limit	CI upper limit	Final rating	**Reason for downgrading**
CDT	USC	.59	-2.74	3.94	LOW	15.68	-.95	32.33	LOW	-1.18	-4.46	02.09	VERY LOW	RoBx2, Imprecisionx2
CDT+CPM	USC	NA	NA	NA	NA	.10	-7.86	08.07	LOW	.10	-7.86	08.07	VERY LOW	RoBx2, Imprecision
LLLT	USC	3	-.78	6.78	LOW	NA	NA	NA	NA	3	-.78	6.78	VERY LOW	RoB, Publication bias, Imprecision
Multimodal	USC	-15	-31.55	1.55	MODERATE	-30.08	-34.44	-25.73	LOW	29.33	25.64	33.03	MODERATE	RoB
Multimodal+H-EXC-PROGRAM	USC	-14.3	-31.47	2.87	MODERATE	-44.47	-65.88	-23.07	MODERATE	27.81	19.90	35.73	MODERATE	RoB
Pilates	USC	3	-1.03	07.03	MODERATE	3	-1.03	07.03	NA	3	-1.03	07.03	LOW	RoB, Imprecision
Yoga	USC	-9.93	-17.57	-2.30	MODERATE	-9.93	-17.57	-2.30	NA	-9.93	-17.57	-2.30	MODERATE	RoB
CDT+CPM	CDT	1.29	-14.46	17.04	MODERATE	8.24	-12557.9	12574.39	LOW	1.29	-5.97	8.55	VERY LOW	RoB, Imprecisionx2
LLLT	CDT	NA	NA	NA	NA	4.18	-.82	9.19	LOW	4.18	-.82	9.19	VERY LOW	RoBx2, Publication bias, Imprecision
Multimodal	CDT	30.69	28.74	32.63	MODERATE	15.59	-2.83	34.03	LOW	30.52	28.75	32.29	VERY LOW	RoB, Imprecisionx2
Multimodal+H-EXC-PROGRAM	CDT	NA	NA	NA	NA	29.00	21.69	36.30	LOW	29.00	21.69	36.30	LOW	RoBx2
Pilates	CDT	NA	NA	NA	NA	4.18	-1.01	9.38	LOW	4.18	-1.01	9.38	VERY LOW	RoBx2, Imprecision
Yoga	CDT	NA	NA	NA	NA	-8.75	-17.06	-.44	LOW	-8.75	-17.06	-.44	LOW	RoBx2
LLLT	CDT+CPM	NA	NA	NA	NA	2.89	-5.92	11.72	LOW	2.89	-5.92	11.72	VERY LOW	RoB, Publication bias, Imprecision
Multimodal	CDT+CPM	NA	NA	NA	NA	29.23	21.75	36.71	MODERATE	29.23	21.75	36.71	MODERATE	RoB
Multimodal+H-EXC-PROGRAM	CDT+CPM	NA	NA	NA	NA	27.71	17.40	38.01	MODERATE	27.71	17.40	38.01	MODERATE	RoB
Pilates	CDT+CPM	NA	NA	NA	NA	2.89	-6.04	11.83	MODERATE	2.89	-6.04	11.83	LOW	RoB, Imprecision
Yoga	CDT+CPM	NA	NA	NA	NA	-10.04	-21.08	.99	MODERATE	-10.04	-21.08	.99	LOW	RoB, Imprecision
Multimodal	LLLT	NA	NA	NA	NA	26.33	21.05	31.62	LOW	26.33	21.05	31.62	LOW	RoB, Publication bias
Multimodal+H-EXC-PROGRAM	LLLT	NA	NA	NA	NA	24.81	16.04	33.59	LOW	24.81	16.04	33.59	LOW	RoB, Publication bias
Pilates	LLLT	NA	NA	NA	NA	-4.44	-5.52	5.52	LOW	-4.44	-5.52	5.52	VERY LOW	RoB, Publication bias, Imprecision
Yoga	LLLT	NA	NA	NA	NA	-12.94	-21.45	-4.42	LOW	-12.94	-21.45	-4.42	LOW	RoB, Publication bias
Multimodal+H-EXC-PROGRAM	Multimodal	-.70	-7.87	6.47	MODERATE	-30.87	-65.03	3.27	MODERATE	-1.52	-8.63	5.59	VERY LOW	RoB, Imprecisionx2
Pilates	Multimodal	NA	NA	NA	NA	-26.33	-31.80	-20.86	MODERATE	-26.33	-31.80	-20.86	MODERATE	RoB
Yoga	Multimodal	NA	NA	NA	NA	-39.27	47.75	-30.80	MODERATE	-39.27	47.75	-30.80	VERY LOW	RoB, Imprecisionx2
Pilates	Multimodal+H-EXC-PROGRAM	NA	NA	NA	NA	-24.81	-33.70	-15.93	MODERATE	-24.81	-33.70	-15.93	MODERATE	RoB
Yoga	Multimodal+H-EXC-PROGRAM	NA	NA	NA	NA	-37.75	-48.75	-26.76	MODERATE	-37.75	-48.75	-26.76	MODERATE	RoB
Yoga	Pilates	NA	NA	NA	NA	-12.94	-21.57	-4.30	MODERATE	-12.94	-21.57	-4.30	MODERATE	RoB

8.14. Grip strength < 6 months:

COMPARISONS		DIRECT ESTIMATE				INDIRECT ESTIMATE				NETWORK ESTIMATE				
		ABSOLUTE ESTIMATE			GRADE	ABSOLUTE ESTIMATE			GRADE	ABSOLUTE ESTIMATE			GRADE	
TREATMENT 1	TREATMENT 2	Point estimate	CI lower limit	CI upper limit	Final rating	Point estimate	CI lower limit	CI upper limit	Final rating	Point estimate	CI lower limit	CI upper limit	Final rating	**Reason for downgrading**
LLLT	USC	-1.45	-6.40	3.49	LOW	.52	-2981.75	2982803	LOW	02.07	-.15	4.31	VERY LOW	RoBx2, Imprecision
Multimodal	USC	2.20	-8.48	12.88	MODERATE	NA	NA	NA	NA	2.20	-8.48	12.88	LOW	RoB, Imprecision
Multimodal+H-EXC-PROGRAM	USC	5	-6.27	16.27	MODERATE	NA	NA	NA	NA	5	-6.27	16.27	LOW	RoB, Imprecision
PCT	USC	NA	NA	NA	NA	03.07	-.46	6.62	LOW	03.07	-.46	6.62	VERY LOW	RoBx2, Imprecision
Pilates	USC	-2.1	-5.02	0.82	MODERATE	NA	NA	NA	NA	-2.1	-5.02	0.82	LOW	RoB, Imprecision
Yoga	USC	3.17	-2.97	9.31	MODERATE	NA	NA	NA	NA	3.17	-2.97	9.31	LOW	RoB, Imprecision
Multimodal	LLLT	NA	NA	NA	NA	0.12	-10.79	11.03	LOW	0.12	-10.79	11.03	VERY LOW	RoBx2, Imprecision
Multimodal+H-EXC-PROGRAM	LLLT	NA	NA	NA	NA	2.92	-8.57	14.41	LOW	2.92	-8.57	14.41	VERY LOW	RoBx2, Imprecision
PCT	LLLT	1	-5.72	7.72	MODERATE	NA	NA	NA	NA	.99	-1.74	3.74	LOW	RoB, Imprecision
Pilates	LLLT	NA	NA	NA	NA	-4.17	-7.86	-.49	LOW	-4.17	-7.86	-.49	LOW	RoBx2
Yoga	LLLT	NA	NA	NA	NA	01.09	-5.44	7.62	LOW	01.09	-5.44	7.62	VERY LOW	RoBx2, Imprecision
Multimodal+H-EXC-PROGRAM	Multimodal	2.79	-6.93	12.53	MODERATE	NA	NA	NA	NA	2.79	-6.93	12.53	LOW	RoB, Imprecision
PCT	Multimodal	NA	NA	NA	NA	.87	-10.37	12.13	MODERATE	.87	-10.37	12.13	LOW	RoB, Imprecision
Pilates	Multimodal	NA	NA	NA	NA	-4.30	-15.37	6.77	MODERATE	-4.30	-15.37	6.77	LOW	RoB, Imprecision
Yoga	Multimodal	NA	NA	NA	NA	.96	-11.35	13.29	MODERATE	.96	-11.35	13.29	LOW	RoB, Imprecision
PCT	Multimodal	NA	NA	NA	AN	.87	-10.37	12.13	MODERATE	.87	-10.37	12.13	LOW	RoB, Imprecision
Pilates	Multimodal+H-EXC-PROGRAM	NA	NA	NA	NA	-7.1	-18.74	4.54	MODERATE	-7.1	-18.74	4.54	LOW	RoB, Imprecision
Yoga	Multimodal+H-EXC-PROGRAM	NA	NA	NA	NA	-1.83	-14.67	11.01	MODERATE	-1.83	-14.67	11.01	LOW	RoB, Imprecision
Pilates	PCT	NA	NA	NA	NA	-5.17	-9.77	-.58	MODERATE	-5.17	-9.77	-.58	MODERATE	RoB
Yoga	PCT	NA	NA	NA	NA	.09	-7.00	7.18	MODERATE	.09	-7.00	7.18	LOW	RoB, Imprecision
Yoga	Pilates	NA	NA	NA	NA	5.27	-1.53	12.07	MODERATE	5.27	-1.53	12.07	LOW	RoB, Imprecision

Supplement S13: Estimators of effect and certainty of evidence for studies not connected to the NMA, by outcome.

**Outcome: Volume of lymphoedema < 6 months**				
**Comparison**	**No. Of studies**	**No. Of participants**	**Effect size (MD IC 95%)**	**GRADE**
Manual lymphatic drainage versus Sequential pneumatic compression	1	24	122.0 (-57.6 – 302.6)	Very Low
Advanced pneumatic compression devices versus Standard pneumatic compression devices	1	36	-99 (-307.8 – 109.8)	Very Low
Kinesiology taping versus Control group	1	28	-92.72 (-248.5 – 63.02)	Very Low
Conventional aquatic exercise versus Low-speed aquatic exercise	1	18	48 (-387.3 – 438.3)	Very Low
Low-frequency low-intensity electrotherapy versus Manual lymphatic drainage	1	32	164.10 (-256.14 – 584.34)	Low
Daytime use of compression sleeve alone versus SC plus nighttime multilayer compression bandaging	1	82	-20 (-155.65 – 115.65)	Very Low
Daytime use of compression sleeve alone versus SC plus NCSG	1	75	18 (-128.21 – 164.21)	Very Low
SC plus nighttime multilayer compression bandaging versus SC plus NCSG	1	79	38 (-90.13 – 166.13)	Very Low
Negative-pressure massage treatment versus Manual lymphatic drainage	1	27	160.32 (-87.33 – 407.97)	Very Low
Multilayer bandage/Mobiderm versus Intermediate-layer bandage	1	49	-125 (-386.4 – 136.4)	Very Low
Complete decongestive therapy with manual lymphatic drainage versus Complete decongestive therapy without manual lymphatic drainage	1	73	14.60 (-77.1 – 101.01)	Low
Manual lymphatic drainage versus Simple lymphatic drainage	1	60	41 (29.33 – 52.67)	Low
Home-based exercise program plus standard lymphoedema self-care versus Standard lymphoedema self-care	1	23	-147.25 (-302.4 – 7.85)	Low
Low-level laser therapy versus Low-level laser therapy sham	1	36	-1.04 (-166.36 – 164.28)	Low
Multilayer bandage versus Simplified multilayer bandage	1	58	-6 (-56.87 – 44.87)	Low
Multilayer bandage versus Cohesive bandage	1	57	-420 (-66.76 – 58.36)	Low
Multilayer bandage versus Adhesive bandage	1	58	58.10 (7.70 – 108.5)	Low
Multilayer bandage versus Kinesiotaping bandage	1	57	101.50 (56.63 – 148.37)	Low
Simplified multilayer bandage versus Cohesive bandage	1	59	1.80 (-46.31 – 49.91)	Very Low
Simplified multilayer bandage versus Adhesive bandage	1	60	64.10 (31.25 – 96.95)	Low
Simplified multilayer bandage versus Kinesiotaping bandage	1	59	107.50 (80.79 – 134.21)	Low
Cohesive bandage versus Adhesive bandage	1	59	62.30 (14.67 – 109.93)	Low
Cohesive bandage versus Kinesiotaping bandage	1	58	105.70 (61.76 – 149.64)	Low
Adhesive bandage versus Kinesiotaping bandage	1	59	43.40 (17.56 – 69.24)	Low
Mobiderm for nighttime use versus Daytime elastic hosiery	1	40	-46.20 (-142.74 – 50.34)	Very Low
**Outcome: Volume of lymphoedema > 6 months**				
Compression bandaging versus Complex decongestive physiotherapy	1	51	115. (-35.57 – 265.77)	Very Low
Self-lymphatic drainage + compression bandaging versus Compression bandaging	1	24	1.10 (-7-71 – 9.91)	Very Low
Daytime use of compression sleeve alone versus SC + nighttime multilayer compression bandaging	1	82	-68 (-209.16 – 73.16)	Very Low
Daytime use of compression sleeve alone versus SC plus NCSG	1	75	-21 (-168.998 – 126.98)	Very Low
SC + nighttime multilayer compression bandaging versus SC + NCSG alone	1	79	47 (-85.67 – 179.67)	Very Low
Complete decongestive therapy with manual lymphatic drainage versus Complete decongestive therapy without manual lymphatic drainage	1	73	28.20 (-63 – 119.87)	Low
Home-based exercise program plus standard lymphoedema self-care versus Standard lymphoedema self-	1	23	-155.58 (-320. – 9.69)	Low

care				
**Outcome: Volume reduction < 6 months**				
Complex decongestive therapy versus Compression therapy (elastic compression garments)	1	95	105 (-79 – 289)	Low
Manual lymphatic drainage massage with multilayer compression bandaging versus Multilayer compression bandaging	1	44	14 (-99.33 – 127.33)	Very Low
Exercising with compression versus Exercising without compression	1	40	34.30 (15.89 – 52.71)	Very Low
Conventional compressive bandaging versus Alginate semi-rigid bandage	1	76	-58 (-128.61 – 12.61)	Very Low
Bandage versus Bandage + kinesiotape	1	27	-130.16 (.313.09 – 52.77)	Very Low
Bandage versus Kinesiotape	1	28	23.65 (-155.85 – 203.15)	Very Low
Bandage + kinesiotape versus Kinesiotape	1	27	153.81 (-29.12 – 336.74)	Very Low
Compression glove and sleeve versus Compression bandages	1	21	47.50 (-62.35 – 157.35)	Very Low
Bandages with low interface pressure (20-30 mm Hg) versus Bandages exerting high interface pressure (44-58 mm Hg).	1	36	110 (-350.91 – 572.91)	Low
Flexitouch (developed to mechanically simulate manual lymphatic drainage) versus Self-administered massage	1	20	-260 (-377 - -142.59)	Very Low
Low-level laser therapy versus No intervention	1	21	-126 (-240.86 - -11.34)	Very Low
**Outcome: Percentage of reduction < 6 months**				
Complex decongestive physiotherapy + intermittent pneumatic compression versus Complex decongestive physiotherapy	1	23	19.30 (2.81 – 35.79)	Very Low
Short-stretch multilayer bandaging versus 3M Coban bandage	1	60	-3.20 (-7.05 – 0.65)	Low
Manual lymphatic drainage massage with multilayer compression bandaging versus Multilayer compression bandaging	1	44	8.90 (-3.23 – 21.03)	Very Low
Advanced pneumatic compression devices versus Standard pneumatic compression devices	1	36	-2.80 (-10.94 – 5.34)	Very Low
Daytime use of compression sleeve alone versus SC plus nighttime multilayer compression bandaging	1	82	-10.60 (-17.96 - -3.24)	Very Low
Daytime use of compression sleeve alone versus SC plus NCSG	1	75	-14.40 (-23.47 - -5.33)	Very Low
SC plus nighttime multilayer compression bandaging versus SC plus NCSG	1	79	-3.80 (-12.20 – 4.60)	Very Low
Complex decongestive therapy versus Complex decongestive therapy + intermittent pneumatic compression	1	76	1.80 (0.60 – 3.00)	Low
Home-based exercise program + standard lymphoedema self-care versus Standard lymphoedema self-care	1	23	-8.33 (-15.54 - -1.12)	Low
Multilayer bandage group versus Simplified multilayer bandage	1	56	-23.20 (-35.73 - -10.67)	Low
Multilayer bandage group versus Cohesive bandage	1	57	-10 (-24.36 – 4.36)	Low
Multilayer bandage group versus Adhesive bandage	1	58	14.60 (3.99 – 25.21)	Low
Multilayer bandage group versus Kinesiotaping bandage	1	57	31.40 (20.67 – 42.13)	Low
Simplified multilayer bandage versus Cohesive bandage	1	57	13.20 (-0.01 – 26.41)	Low
Simplified multilayer bandage versus Adhesive bandage	1	58	37.80 (28.75 – 46.85)	Low
Simplified multilayer bandage versus Kinesiotaping bandage	1	57	54.60 (45.47 – 63.73)	Low
Cohesive bandage versus Adhesive bandage	1	59	24.60 (13.19 – 36.01)	Low
Cohesive bandage versus Kinesiotaping bandage	1	58	41.40 (29.85 – 52.95)	Low
Adhesive bandage versus Kinesiotaping bandage	1	59	16.80 (10.07 – 23.53)	Low
**Outcome: Percentage of reduction > 6 months**				
Self-administered complex decongestive therapy versus Usual care	1	34	2.0 (-7.14 – 11.14)	Low
Home-based exercise program + standard lymphoedema self-care versus Standard lymphoedema self-care	1	23	-8.49 (-16.74 - -0.24)	Low
**Outcome: Global quality of life < 6 months**				

Resistance exercise program versus Complex decongestive therapy + resistance exercise program	1	44	-0.70 (-12.95 – 11.55)	Very Low
Kinesiotaping versus Compression garment	1	59	7.30 (0.18 – 14.42)	Low
Low-frequency low-intensity electrotherapy versus Manual lymphatic drainage	1	32	7.25 (-2.55 – 17.05)	Low
Complete decongestive therapy versus Kinesiology taping	1	36	-2.20 (-7.89 – 3.49)	Very Low
Short-stretch multilayer bandaging versus 3M Coban bandage	1	60	0.70 (-0.16 – 1.56)	Very Low
Manual lymphatic drainage versus Kinesiotaping	1	33	6.80 (-0.69 – 14.29)	Very Low
Manual lymphatic drainage versus Low-level laser therapy	1	30	3.24 (-4.50 – 10.98)	Very Low
Kinesiotaping versus Low-level laser therapy	1	33	-3.56 (-12.61 – 5.49)	Very Low
Decongestive lymphatic therapy + fluoroscopy-guided manual lymphatic drainage versus Decongestive lymphatic therapy + traditional manual lymphatic drainage	1	129	-0.10 (-0.80 – 0.60)	Low
Decongestive lymphatic therapy + fluoroscopy-guided manual lymphatic drainage versus Manual lymphatic drainage	1	130	-0.17 (-0.86 – 0.52)	Low
Decongestive lymphatic therapy + traditional manual lymphatic drainage versus Manual lymphatic drainage	1	129	-0.07 (-0.77 – 0.63)	Low
Aerobic-based exercise versus Resistance-based exercise	1	41	6.60 (-6.64 – 19.84)	Low
Low-level laser therapy versus Manual lymphatic drainage	1	31	7.80 (0.66 – 14.94)	Very Low
Low-level laser therapy versus Combined manual lymphatic drainage + low-level laser therapy	1	30	11.10 (4.43 – 17.77)	Very Low
Manual lymphatic drainage versus Combined manual lymphatic drainage + low-level laser therapy	1	31	3.30 (-2.58 – 9.18)	Very Low
Complete decongestive therapy + continuous passive motion versus Complete decongestive therapy	1	30	13.10 (1.13 – 25.07)	Very Low
Aqua lymphatic therapy (ALT) versus Self-care	1	48	QoL improved in the ALT.	-
Sleeves + resistance training program + aerobic exercises (S+RP+A) versus Sleeves without int (S+W-I)	1	14	Increases in the exercise group: physical functioning, general health, and vitality. Mental health increased, although not significantly, for all subjects.	-
Home-based exercise program plus standard lymphoedema self-care versus Standard lymphoedema self-care	1	23	There was no improvement in QoL.	-
Bandage versus kinesiotaping	1	41	Improvement in emotional function in the bandage group.	-
The Breast Cancer Recovery Program versus continuing with the lymphedema instructions from their medical team (usual self-care)	1	22	Improved physical function, general health, and vitality in the Breast Cancer Recovery Program group.	-
Activity-oriented antiedema proprioceptive therapy (TAPA) treatment versus Control group	1	51	Improved social function (TAPA).	-
Manual lymphatic drainage versus Simple lymphatic drainage	1	31	QoL, in terms of emotional function, dyspnea, and sleep disturbance, and a number of altered sensations, such as pain and heaviness, were also significantly improved by manual lymphatic drainage.	-
**Outcome: Global quality of life > 6 months**				

Multimodal versus Multimodal+H-EXC-PROGRAM	1	25	5.0 (-17.76 – 27.76)	Very Low
Multimodal versus Usual care	1	21	18.20 (-4.82 – 41.22)	Very Low
Multimodal+H-EXC-PROGRAM versus Usual care	1	22	13.20 (-12.90 – 39.30)	Very Low
Decongestive lymphatic therapy + fluoroscopy-guided manual lymphatic drainage versus Decongestive lymphatic therapy + traditional manual lymphatic drainage	1	129	0.27 (-0.45 – 0.99)	Low
Decongestive lymphatic therapy + fluoroscopy-guided manual lymphatic drainage versus Manual lymphatic drainage	1	126	0.15 (-0.57 – 0.87)	Low
Decongestive lymphatic therapy + traditional manual lymphatic drainage versus Manual lymphatic drainage	1	125	-0.12 (-0.84 – 0.60)	Low
**Outcome: Pain < 6 months**				
High-load resistance exercise versus Low-load resistance exercise	1	36	-0.10 (-0.9 – 0.71)	Very Low
High-load resistance exercise versus Own taping	1	41	0.14 (-0.30 – 0.58)	Very Low
Low-load resistance exercise versus Own taping	1	37	0.24 (-0.48 – 0.96)	Very Low
Xbox Kinect versus Resistance exercise	1	37	0.24 (-0.48 – 0.96)	Low
Aqua therapy resistance exercise program versus Land-based exercise therapy	1	50	-2.52 (-3.09 - -1.95)	Low
Kinesiotaping versus Compression garment	1	59	-19.20 (-24.36 - -14.04)	Low
Kinesiotaping versus Compression garment + education + preventive measures + exercises	1	35	0.37 (-0.41 – 1.15)	Low
Extracorporeal shockwaves versus Complex decongestive therapy	1	30	0.03 (-0.05 – 0.61)	Very Low
Manual lymphatic drainage, low elastic compression garment, gleno-humeral mobilization, and deep-breathing exercises versus Complete decongestive therapy	1	60	1.50 (1.27 – 1.73)	Low
Complex decongestive therapy versus Self-administered complex decongestive therapy	1	40	1.90 (0.20 – 3.60)	Low
Bandage versus Kinesiotape	1	28	-1.15 (-1.57 - - 0.73)	Very Low
Bandage versus Bandage + kinesiotape	1	27	-0.42 (-0.86 – 0.02)	Very Low
Kinesiotape versus Bandage + kinesiotape	1	27	0.73 (0.29 – 1.17)	Very Low
Aerobic exercise + resistance exercise/strength versus Complete decongestive therapy	1	63	-10.99 (-13.68 - -8.30)	Very Low
Pneumatic compression therapy versus Low-level laser therapy	1	47	5.50 (-4.13 – 15.13)	Very Low
Manual lymphatic drainage versus Without manual lymphatic drainage	1	57	0.37 (-2.10 – 1.36)	Very Low
Aqua lymphatic therapy (ALT) + home land-based exercise program versus Home land-based exercise program alone	1	18	0.50 (0.38 – 0.62)	Very Low
Complex decongestive physical therapy versus Complex decongestive physical therapy + intermittent pneumatic compression pump	1	30	0.0 (-0.79 – 0.79)	Low
Pneumatic compression group versus Skin care + compression bandage + exercise therapy + manual lymphatic drainage	1	25	-10.0 (-17.90 - -2.10)	Very Low
Complete decongestive therapy with manual lymphatic drainage versus Complete decongestive therapy without manual lymphatic drainage	1	73	-0.10 (-0.38 – 0.18)	Low
Low-intensity and extremely-low-frequency electrostatic fields (Deep Oscillation®) versus Manual lymphatic drainage	1	21	-2.60 (-4.81 - -0.39)	Low
Complex decongestive therapy versus Complex decongestive therapy + intermittent pneumatic compression	1	76	0.00 (-0.53 – 0.53)	Low
Intermittent pneumatic compression + compression bandage + home exercise program versus Manual lymphatic drainage + compression bandage + home exercise program.	1	27	Pain reduction in all groups.	
Resistance exercises versus Resistance exercises + compression garments	1	19	No significant improvements.	-
Proprioceptive neuromuscular facilitation versus Proprioceptive neuromuscular facilitation (PNF) + manual lymphatic drainage (MLD)	1	35	Pain reduction.	-
Proprioceptive neuromuscular facilitation versus Manual lymphatic drainage	1	37	Pain reduction.	-

Proprioceptive neuromuscular facilitation + manual lymphatic drainage versus Manual lymphatic drainage	1	38	Pain reduction.	-
**Outcome: Pain > 6 months**				
Complete decongestive therapy versus Usual care	1	34	1.70 (-0.29 – 3.69)	Low
Pneumatic compression therapy versus Low-level laser therapy	1	47	5.10 (-5.59 – 15.79)	Very Low
Complete decongestive therapy with manual lymphatic drainage versus Complete decongestive therapy without manual lymphatic drainage	1	73	-0.20 (-0.48 – 0.08)	Low
**Outcome: Joint range elbow extension < 6 months**				
High-load resistance exercise versus Low-load resistance exercise	1	43	-1.90 (-4.66 – 0.86)	Very Low
High-load resistance exercise versus Own taping	1	41	-2.00 (-4.63 – 0.63)	Very Low
Low-load resistance exercise versus Own taping	1	40	0.10 (-2.41 – 2.61)	Very Low
**Outcome: Joint range elbow flexion < 6 months**				
High-load resistance exercise versus Low-load resistance exercise	1	43	1.80 (-1.97 – 5.57)	Very Low
High-load resistance exercise versus Control group	1	41	-0.60 (-4.17 – 2.97)	Very Low
Low-load resistance exercise versus Control group	1	40	2.40 (-1.20 – 6.00)	Very Low
Kinesiotaping + pneumatic compression + manual lymphatic drainage versus Quasi-KT + pneumatic compression + manual lymphatic drainage	1	45	1.76 (-1.16 – 4.68)	Very Low
Kinesiotaping + pneumatic compression + manual lymphatic drainage versus Pneumatic + manual lymphatic drainage + multilayer bandaging	1	47	-6.89 (-8.98 - -4.80)	Very Low
Quasi-KT + pneumatic compression + manual lymphatic drainage versus Pneumatic + manual lymphatic drainage + multilayer bandaging	1	48	8.65 (6.64 – 10.66)	Very Low
Manual lymphatic drainage versus Sequential pneumatic compression	1	24	No change in elbow flexion.	-
Control group versus Pneumatic compression group	1	25	No significant differences between the groups.	-
Own taping versus Traditional kinesiotaping	1	44	No significant differences between the groups.	-
**Outcome: Joint range wrist extension < 6 months**				
High-load resistance exercise versus Low-load resistance exercise	1	43	-4.30 (-9.28 – 0.68)	Very Low
High-load resistance exercise versus Control group	1	41	-0.80 (-6.52 – 4.92)	Very Low
Low-load resistance exercise versus Control group	1	40	-3.50 (-8.93 – 1.93)	Very Low
Own taping versus Traditional kinesiotaping	1	44	No significant differences between the groups.	-
Kinesiotaping versus Compression garment	1	30	After applying kinesiotaping, there was a post-intervention increase in the range of motion.	-
Control group versus Pneumatic compression group	1	25	No significant differences between the groups.	-
**Outcome: Joint range wrist flexion < 6 months**				
Own taping versus Traditional kinesiotaping	1	44	No significant differences between the groups.	-
Control group versus Pneumatic compression group	1	25	No significant differences between the groups.	-
**Outcome: Joint range shoulder abduction < 6 months**				
Low-intensity resistance exercises + wearing compression garment versus Low-intensity resistance training	1	54	4.10 (-2.34 – 10.54)	Very Low

Kinesiotaping versus Compression garment	1	35	-7.89 (-21.54 – 5.76)	Low
Kinesiotaping + pneumatic compression + manual lymphatic drainage versus Quasi-KT + pneumatic compression + manual lymphatic drainage	1	45	-0.81 (-5.26 – 3.64)	Very Low
Kinesiotaping + pneumatic compression + manual lymphatic drainage versus Pneumatic + manual lymphatic drainage + multilayer bandaging	1	47	-9.92 (-14.71 - -5.13)	Very Low
Quasi-KT + pneumatic compression + manual lymphatic drainage versus Pneumatic + manual lymphatic drainage + multilayer bandaging	1	47	-9.11 (-13.64 - -4.58)	Very Low
Intermittent pneumatic compression (IPC) + compression bandage versus Manual lymphatic drainage (mld) + compression bandage (cb)	1	46	2.99 (-6.25 – 12.23)	Very Low
Water-based exercise versus Pilates	1	68	6.87 (3.03 – 10.71)	Low
Water-based exercise versus Instructions to continue exercises	1	25	-13.00 (-44.09 – 18.09)	Very Low
Pneumatic compression versus Skin care + compression bandage + exercise therapy + manual lymphatic drainage	1	25	-5.00 (-18.73 – 8.73)	Very Low
High-load resistance exercise versus Low-load resistance exercise	1	43	-8.90 (-59.51 – 41.71)	Very Low
High-load resistance exercise versus Control group	1	41	-8.30 (-65.17 – 48.67)	Very Low
Low-load resistance exercise versus Control group	1	40	0.60 (-38.82 – 40.02)	Very Low
**Outcome: Joint range shoulder abduction < 6 months**				
Kinesiotaping versus Compression garment	1	35	0.86 (-2.96 – 4.68)	Low
Complex exercise versus Conventional decongestive therapy	1	63	2.50 (0.90 – 4.10)	Very Low
Intermittent pneumatic compression + compression bandage versus Manual lymphatic drainage + compression bandage	1	46	1.81 (-2.30 – 5.92)	Very Low
Home-based exercise program + standard lymphoedema self-care versus Standard lymphoedema self-care	1	23	There was no improvement in ROM.	-
**Outcome: Joint range shoulder extension < 6 months**				
High-load resistance exercise versus Low-load resistance exercise	1	43	-8.60 (-39.80 – 22.60)	Very Low
High-load resistance exercise versus Control group	1	41	-6.00 (-37.52 – 25.52)	Very Low
Low-load resistance exercise versus Control group	1	40	2.60 (-30.20 – 35.40)	Very Low
Yoga versus Usual care	1	19	-11.82 (-20.16 - -3.48)	Very Low
Kinesiotaping versus Compression garment	1	35	0.86 (-2.96 – 4.68)	Low
Aerobic exercise + resistance exercise/strength versus Complete decongestive therapy	1	63	2.50 (0.90 – 4.10)	Very Low
Intermittent pneumatic compression + compression bandage + home exercise program versus Manual lymphatic drainage + compression bandage + home exercise program.	1	46	1.81 (-2.30 – 5.92)	Very Low
Water-based exercise versus Pilates	1	68	0.67 (-1.71 – 3.05)	Low
Home-based exercise program plus standard lymphoedema self-care versus Standard lymphoedema self-care	1	23	There was no improvement in ROM.	-
Kinesiotaping versus Traditional taping	1	44	Improvement of 20° in the kinesiotaping group.	-
**Outcome: Joint range shoulder flexion < 6 months**				
High-load resistance exercise versus Low-load resistance exercise	1	43	-6.80 (-48.87 – 35.27)	Very Low
High-load resistance exercise versus Control group	1	41	-1.40 (-49.01 – 46.21)	Very Low
Low-load resistance exercise versus Control group	1	40	5.40 (-31.57 – 42.37)	Very Low
Xbox Kinect versus Resistance exercise	1	60	13.00 (8.42 – 17.58)	Low
Kinesiotaping versus Compression garment	1	35	-2.89 (-14.18 – 8.40)	Low

Kinesiotaping + pneumatic compression + manual lymphatic drainage versus Quasi-KT + pneumatic compression + manual lymphatic drainage	1	45	-1.19 (-10.21 – 7.83)	Very Low
Kinesiotaping + pneumatic compression + manual lymphatic drainage versus Pneumatic + manual lymphatic drainage + multilayer bandaging	1	47	-10.21 (-16-89 - -3.53)	Very Low
Quasi-KT + pneumatic compression + manual lymphatic drainage versus Pneumatic + manual lymphatic drainage + multilayer bandaging	1	47	-9.02 (-16.49 - -1.55)	Very Low
Intermittent pneumatic compression + compression bandage + home exercise program versus Manual lymphatic drainage + compression bandage + home exercise program.	1	46	3.40 (-6.14 – 12.94)	Very Low
Water-based exercise versus Pilates	1	68	8.73 (3.54 – 13.92)	Low
Manual lymphatic drainage versus Sequential pneumatic compression	1	25	-6.00 (-20.90 – 8.90)	Very Low
Pneumatic compression versus Usual care	1	25	10.00 (-5.08 – 25.08)	Very Low
Kinesiotaping versus Compression garment	1	30	After applying kinesiotaping, there was a post-intervention increase in the range of motion.	-
Kinesiotaping versus Traditional taping	1	44	A 20° improvement was reported in the kinesiotaping group.	-
Manual lymphatic drainage + proprioceptive neuromuscular facilitation versus Manual lymphatic drainage	1	35	Increase in ROM.	-
Manual lymphatic drainage + proprioceptive neuromuscular facilitation versus Proprioceptive neuromuscular facilitation	1	37	Increase in ROM.	-
Manual lymphatic drainage versus Proprioceptive neuromuscular facilitation	1	38	Increase in ROM.	-
Water-Based Exercise versus Instructions to continue exercise	1	24	No change in ROM.	-
Low-level laser therapy versus Low-level laser therapy sham	1	61	No report of any consistent effect on ROM.	-
**Outcome: Joint range external shoulder rotation < 6 months**				
Xbox Kinect versus Resistance exercise	1	60	8.93 (6.19 – 11.67)	Low
Kinesiotaping versus Compression garment	1	35	-5.56 (-11.09 – 0.03)	Low
Intermittent pneumatic compression + compression bandage versus Manual lymphatic drainage + compression bandage	1	46	5.07 (-2.41 – 12.55)	Low
Pneumatic compression versus Usual care	1	25	0.00 (-9.18 – 9.18)	Very Low
Water-based exercise versus Pilates	1	68	0.67 (-3.18 – 4.52)	Low
Manual lymphatic drainage versus Sequential pneumatic compression	1	81	10.00 (-17.05 – 37.05)	Very Low
Water-based Exercise versus Instructions to continue exercise	1	24	No change in ROM	-
**Outcome: Joint range internal shoulder rotation < 6 months**				
Kinesiotaping versus Compression garment	1	19	-4.89 (-19.97 – 10.19)	Low
Intermittent pneumatic compression + compression bandage versus Manual lymphatic drainage + compression bandage	1	46	3.29 (-0.26 – 6.84)	Very Low
Manual lymphatic drainage versus Sequential pneumatic compression	1	23	10.0 (0.08 – 19.92)	Very Low
Yoga versus Usual care	1	19	-4.89 (-19.97 – 10.19)	Low
Water-based exercise versus Pilates	1	68	1.93 (-1.05 – 4.91)	Low
Water-based Exercise versus Instructions to continue exercise	1	24	No change in ROM.	-
**Outcome: Grip strength < 6 months**				
High-load resistance exercise versus Low-load resistance exercise	1	43	0.60 (-17.75 – 18.95)	Very Low

High-load resistance exercise versus Control	1	43	0.60 (-17.75 – 18.95)	Very Low
Low-load resistance exercise versus Control	1	54	0.00 (-16.34 – 16.34)	Very Low
Xbox Kinect versus Resistance exercise	1	60	-2.77 (-4.12 - -1.42)	Low
Aqua lymphatic therapy (ALT) + home land-based exercise program versus Home land-based exercise program alone	1	18	-0.10 (-6.61 – 6.41)	Very Low
Kinesiotaping versus Compression garment	1	59	8.10 (5.21 – 10.99)	Low
Extracorporeal shockwave therapy versus Intermittent pneumatic compression therapy	1	43	0.86 (-0.14 – 1.86)	Low
	1	20	3.83 (0.99 – 6.67)	Very Low
Complete decongestive therapy versus Kinesiology taping	1	36	1.00 (-1.19 – 3.19)	Very Low
Complex decongestive therapy versus Complex decongestive therapy + intermittent pneumatic compression	1	76	-1.50 (-1.78 - -1.22)	Low
Own taping versus Traditional taping	1	44	The grip strength improved by 8 kg in the study group after the applied therapy and by 5 kg in the clinical control group.	-
KT + pneumatic + manual lymphatic drainage versus Quasi-KT + pneumatic + manual lymphatic drainage	1	55	Grip strength increased significantly.	-
KT + pneumatic + manual lymphatic drainage versus Standard procedure—pneumatic + manual lymphatic drainage + multilayer bandaging	1	47	Grip strength increased significantly	-
Quasi-KT + pneumatic + manual lymphatic drainage versus Standard procedure—pneumatic + manual lymphatic drainage + multilayer bandaging	1	48	Grip strength increased significantly	-
Manual lymphatic drainage versus Sequential pneumatic compression	1	24	No significant changes in grip strength.	-
**Outcome: Pressure strength > 6 months**				
Complete decongestive therapy versus Kinesiology taping	1	36	1.50 (-0.53 – 3.53)	Very Low
Pneumatic compression therapy versus Low-level laser therapy	1	47	-1.20 (-4.12 – 1.72)	Very Low

Supplement S14: Network meta-analysis plots.

Volume of lymphoedema < 6 months

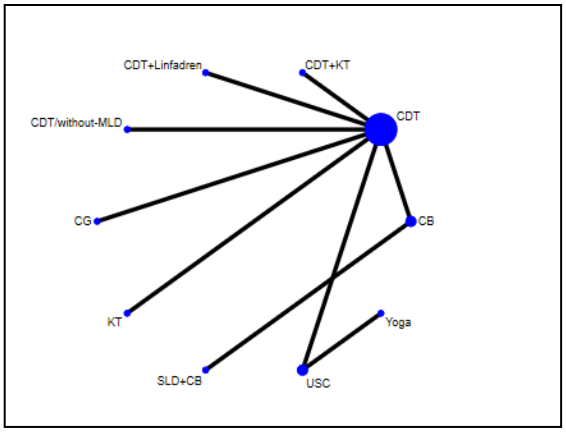

Percentage of reduction < 6 months

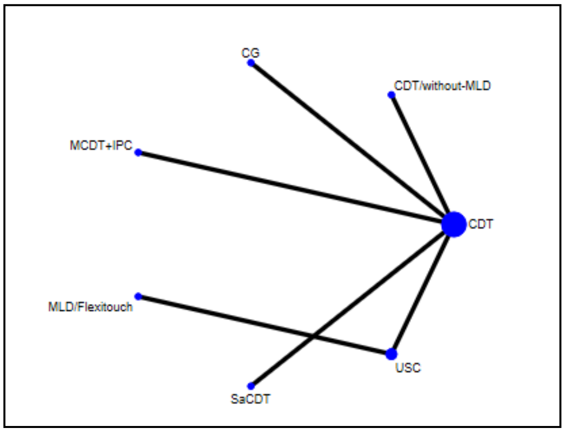

Supplement S15. Absolute effect estimates and certainty of evidence.

12. 1. Volume of lymphoedema < 6 months:

COMPARISONS		DIRECT ESTIMATE				INDIRECT ESTIMATE				NETWORK ESTIMATE				
		ABSOLUTE ESTIMATE			GRADE	ABSOLUTE ESTIMATE			GRADE	ABSOLUTE ESTIMATE			GRADE	
TREATMENT 1	TREATMENT 2	Point estimate	CI lower limit	CI upper limit	Final rating	Point estimate	CI lower limit	CI upper limit	Final rating	Point estimate	CI lower limit	CI upper limit	Final rating	**Reason for downgrading**
CB	CDT	-197.10	-357.03	-37.16	MODERATE	-.41	-5.621.378	5.620.545	MODERATE	196.94	37.07	356.80	VERY LOW	RoB, Imprecisionx2
CDT+KT	CDT	42.79	-140.83	226.43	LOW	NA	NA	NA	NA	42.79	-140.83	226.43	VERY LOW	RoBx2, Imprecisionx2
CDT+Linfadren	CDT	-321	-379.64	-262.35	MODERATE	NA	NA	NA	AN	-321	-379.64	-262.35	MODERATE	RoB
CDT/without-MLD	CDT	57.17	-107.80	222.14	LOW	NA	NA	NA	NA	57.17	-107.80	222.14	VERY LOW	RoBx2, Imprecisionx2
CG	CDT	-19	-151.58	113.58	MODERATE	NA	NA	NA	NA	-19	-151.58	113.58	VERY LOW	RoB, Imprecisionx2
KT	CDT	25.3	-24.90	75.50	LOW	NA	NA	NA	NA	25.3	-24.90	75.50	VERY LOW	RoBx2, Imprecision
SLD+CB	CDT	NA	NA	NA	NA	195.94	35.91	355.96	LOW	195.94	35.91	355.96	VERY LOW	RoBx2, Imprecisionx2
USC	CDT	-88.8	-199.69	22.09	MODERATE	4.66	-106.23	115.56	MODERATE	-88.79	-199.69	22.09	VERY LOW	RoB, Imprecisionx2
Yoga	CDT	NA	NA	NA	NA	-99.29	-433.20	234.60	MODERATE	-99.29	-433.20	234.60	VERY LOW	RoB, Imprecisionx2
CDT+KT	CB	NA	NA	NA	NA	-154.14	-397.61	89.32	LOW	-154.14	-397.61	89.32	VERY LOW	RoBx2, Imprecisionx2
CDT+Linfadren	CB	NA	NA	NA	NA	86.87	-347.66	-688.22	MODERATE	86.87	-347.66	-688.22	VERY LOW	RoB, Imprecisionx2
CDT/without-MLD	CB	NA	NA	NA	NA	-139.77	-369.49	89.95	LOW	-139.77	-369.49	89.95	VERY LOW	RoBx2, Imprecisionx2
CG	CB	NA	NA	NA	NA	-215.94	423.63	-8.25	MODERATE	-215.94	423.63	-8.25	VERY LOW	RoB, Imprecisionx2
KT	CB	NA	NA	NA	NA	-171.64	-339.20	-4.08	LOW	-171.64	-339.20	-4.08	VERY LOW	RoBx2, Imprecision
SLD+CB	CB	-1	-8.44	6.44	LOW	-394.36	-11031.17	10242.44	LOW	-1.00	-8.18	6.18	VERY LOW	RoBx2, Imprecision
USC	CB	NA	NA	NA	NA	-285.74	-480.30	-91.17	MODERATE	-285.74	-480.30	-91.17	VERY LOW	RoB, Imprecisionx2
Yoga	CB	NA	NA	NA	NA	-296.24	-666.44	73.96	MODERATE	-296.24	-666.44	73.96	VERY LOW	RoB, Imprecisionx2
CDT+Linfadren	CDT+KT	NA	NA	NA	NA	-363.8	-556.57	-171.02	MODERATE	-363.8	-556.57	-171.02	VERY LOW	RoB, Imprecisionx2
CDT/without-MLD	CDT+KT	NA	NA	NA	NA	14.37	-232.48	261.22	LOW	14.37	-232.48	261.22	VERY LOW	RoBx2, Imprecisionx2
CG	CDT+KT	NA	NA	NA	NA	-61.79	-288.29	164.69	MODERATE	-61.79	-288.29	164.69	VERY LOW	RoB, Imprecisionx2
KT	CDT+KT	NA	NA	NA	NA	-17.49	-207.87	172.87	LOW	-17.49	-207.87	172.87	VERY LOW	RoBx2, Imprecisionx2
SLD+CB	CDT+KT	NA	NA	NA	NA	15.314	-90.43	396.71	LOW	15.314	-90.43	396.71	VERY LOW	RoBx2, Imprecisionx2
USC	CDT+KT	NA	NA	NA	NA	-131.6	-346.12	82.92	MODERATE	-131.6	-346.12	82.92	VERY LOW	RoB, Imprecisionx2
Yoga	CDT+KT	NA	NA	NA	NA	-142.09	-523.17	238.97	LOW	-142.09	-523.17	238.97	VERY LOW	RoBx2, Imprecisionx2
CDT/without-MLD	CDT+Linfadren	NA	NA	NA	NA	378.17	203.08	553.25	LOW	378.17	203.08	553.25	VERY LOW	RoBx2, Imprecisionx2
CG	CDT+Linfadren	NA	NA	NA	NA	302	157.02	446.97	MODERATE	302	157.02	446.97	LOW	RoB, Imprecision
KT	CDT+Linfadren	NA	NA	NA	NA	346.3	269.09	423.50	LOW	346.3	269.09	423.50	VERY LOW	RoBx2, Imprecision
SLD+CB	CDT+Linfadren	NA	NA	NA	NA	516.94	346.50	68.737	MODERATE	516.94	346.50	68.737	LOW	RoB, Imprecision
USC	CDT+Linfadren	NA	NA	NA	NA	232.2	106.74	357.65	MODERATE	232.2	106.74	357.65	LOW	RoB, Imprecision
Yoga	CDT+Linfadren	NA	NA	NA	NA	221.70	-11.731	560.71	MODERATE	221.70	-11.731	560.71	VERY LOW	RoB, Imprecisionx2
CG	CDT/without-MLD	NA	NA	NA	NA	-76.17	-287.81	135.47	LOW	-76.17	-287.81	135.47	VERY LOW	RoBx2, Imprecisionx2
KT	CDT/without-MLD	NA	NA	NA	NA	-31.87	-204.31	140.57	LOW	-31.87	-204.31	140.57	VERY LOW	RoBx2, Imprecisionx2
SLD+CB	CDT/without-MLD	NA	NA	NA	NA	38.77	-91.06	368.60	LOW	38.77	-91.06	368.60	VERY LOW	RoBx2, Imprecisionx2
USC	CDT/without-MLD	NA	NA	NA	NA	-145.97	-344.75	52.81	LOW	-145.97	-344.75	52.81	VERY LOW	RoBx2, Imprecisionx2
Yoga	CDT/without-MLD	NA	NA	NA	NA	-156.46	-528.90	215.96	LOW	-156.46	-528.90	215.96	VERY LOW	RoBx2, Imprecisionx2
KT	CG	NA	NA	NA	NA	44.3	-97.47	186.07	LOW	44.3	-97.47	186.07	VERY LOW	RoBx2, Imprecisionx2
SLD+CB	CG	NA	NA	NA	NA	214.94	7.12	422.75	MODERATE	214.94	7.12	422.75	VERY LOW	RoB, Imprecisionx2
USC	CG	NA	NA	NA	NA	-69.79	-242.64	103.04	MODERATE	-69.79	-242.64	103.04	VERY LOW	RoB, Imprecisionx2
Yoga	CG	NA	NA	NA	NA	-80.29	-439.56	278.96	MODERATE	-80.29	-439.56	278.96	VERY LOW	RoB, Imprecisionx2
SLD+CB	KT	NA	NA	NA	NA	170.64	2.92	338.35	LOW	170.64	2.92	338.35	VERY LOW	RoBx2, Imprecisionx2
USC	KT	NA	NA	NA	NA	-114.1	-235.83	7.63	LOW	-114.1	-235.83	7.63	VERY LOW	RoBx2, Imprecisionx2
Yoga	KT	NA	NA	NA	NA	-124.59	-462.25	213.05	LOW	-124.59	-462.25	213.05	VERY LOW	RoBx2, Imprecisionx2
USC	SLD+CB	NA	NA	NA	NA	-284.74	-47.943	-90.04	MODERATE	-284.74	-47.943	-90.04	VERY LOW	RoB, Imprecisionx2
Yoga	SLD+CB	NA	NA	NA	NA	-295.24	-665.51	75.03	LOW	-295.24	-665.51	75.03	VERY LOW	RoBx2, Imprecisionx2
Yoga	USC	-10.5	-325.45	304.45	MODERATE	176.43	-510191.7	510544.6	MODERATE	-10.49	-325.45	304.45	VERY LOW	RoB, Imprecisionx2

12.2. Percentage of reduction < 6 months:

TREATMENT 1	TREATMENT 2	Point estimate	CI lower limit	CI upper limit	Final rating	Point estimate	CI lower limit	CI upper limit	Final rating	Point estimate	CI lower limit	CI upper limit	Final rating	**Reason for downgrading**
CDT/without-MLD	CDT	06.02	-2.77	14.83	LOW	NA	NA	NA	NA	06.02	-2.77	14.83	VERY LOW	RoBx2, Imprecision
CG	CDT	-1	-6.21	4.21	MODERATE	NA	NA	NA	NA	-1	-6.21	4.21	LOW	RoB, Imprecision
MCDT+IPC	CDT	9.4	-4.11	22.91	MODERATE	NA	NA	NA	NA	9.4	-4.11	22.91	LOW	RoB, Imprecision
MLD/Flexitouch	CDT	NA	NA	NA	NA	-5.09	-12.71	2.51	MODERATE	-5.09	-12.71	2.51	LOW	RoB, Imprecision
SaCDT	CDT	6	-2.44	14.44	HIGH	6	-2.44	14.44	NA	6	-2.44	14.44	MODERATE	Imprecision
USC	CDT	-3.09	-8.47	2.27	MODERATE	NA	NA	NA	NA	-3.09	-8.13	1.93	LOW	RoB, Imprecision
CG	CDT/without-MLD	NA	NA	NA	NA	-7.02	-17.26	3.20	MODERATE	-7.02	-17.26	3.20	LOW	RoB, Imprecision
MCDT+IPC	CDT/without-MLD	NA	NA	NA	NA	3.37	-12.76	19.50	MODERATE	3.37	-12.76	19.50	LOW	RoB, Imprecision
MLD/Flexitouch	CDT/without-MLD	NA	NA	NA	NA	-11.12	-22.77	.51	HIGH	-11.12	-22.77	.51	MODERATE	Imprecision
SaCDT	CDT/without-MLD	NA	NA	NA	NA	-.02	-12.23	12.17	HIGH	-.02	-12.23	12.17	MODERATE	Imprecision
USC	CDT/without-MLD	NA	NA	NA	NA	-9.12	-19.27	01.01	MODERATE	-9.12	-19.27	01.01	LOW	RoB, Imprecision
MCDT+IPC	CG	NA	NA	NA	NA	10.4	-4.08	24.88	MODERATE	10.4	-4.08	24.88	LOW	RoB, Imprecision
MLD/Flexitouch	CG	NA	NA	NA	NA	-4.09	-13.32	5.12	MODERATE	-4.09	-13.32	5.12	LOW	RoB, Imprecision
SaCDT	CG	NA	NA	NA	NA	7	-2.92	16.92	MODERATE	7	-2.92	16.92	LOW	RoB, Imprecision
USC	CG	NA	NA	NA	NA	-2.09	-9.34	5.14	MODERATE	-2.09	-9.34	5.14	LOW	RoB, Imprecision
MLD/Flexitouch	MCDT+IPC	NA	NA	NA	NA	-14.49	-30.01	01.01	MODERATE	-14.49	-30.01	01.01	LOW	RoB, Imprecision
SaCDT	MCDT+IPC	NA	NA	NA	NA	-3.4	-19.33	12.53	MODERATE	-3.4	-19.33	12.53	LOW	RoB, Imprecision
USC	MCDT+IPC	NA	NA	NA	NA	-12.49	-26.92	1.92	MODERATE	-12.49	-26.92	1.92	LOW	RoB, Imprecision
SaCDT	MLD/Flexitouch	NA	NA	NA	NA	11.09	-.26	22.46	HIGH	11.09	-.26	22.46	MODERATE	Imprecision
USC	MLD/Flexitouch	2	-3.79	7.79	MODERATE	NA	NA	NA	NA	1.99	-3.71	7.71	LOW	RoB, Imprecision
USC	SaCDT	NA	NA	NA	NA	-9.09	-18.92	.72	HIGH	-9.09	-18.92	.72	MODERATE	Imprecision

Supplement S16. Summary of effects compared to CDT.

Intervention	Volume of lymphoedema < 6 months DM (IC 95)	Percentage of reduction in lymphoedema < 6 months DM (IC 95)
CB	196.94 (37.07 a 356.80)	
CDT+KT	42.79 (-140.83 a 226.43)	
CDT+Linfadren	-321,00 (-379.64 a -262.35)	
CDT/without MLD	57.17 (-107.80 a 222.14)	6.02 (-2.77 a 14.83)
CG	-19 (-151.58 a 113.58)	-1,00 (-6.21 a 4.21)
KT	25.3 (-24.90 a 75.50)	
SLD+CB	195.94 (35.91 a 355.96)	
USC	-88.79 (-199.69 a 22.09)	-3.09 (-8.13 a 1.93)
Yoga	-99.29 (-433.20 a 234.60)	
MCDT+IPC		9.4 (-4.11 a 22.91)
MLD/Flexitouch		-5.09 (-12.71 a 2.51)
SaCDT		6,00 (-2.44 a 14.44)

	Among the most effective	Intermediate benefit	Among the least effective
High- or Moderate-Certainty Evidence	Better than usual care and some alternatives	Better than usual care but not better than any other alternative	Not better than usual care
Low-Certainty Evidence	May be better than usual care and some alternatives	May be better than usual care but not better than any other alternative	May not be better than usual care
Very-Low-Certainty Evidence	Uncertainty about whether the intervention is better or worse than usual care.		
Without Evidence			

The numbers in the colored cells are the estimated mean difference with their 95% confidence interval for each intervention compared to CDT. Empty cells indicate that there was no evidence for the specific intervention. The bold text represents statistical significance.
